# Accessible chromatin reveals regulatory mechanisms underlying cell fate decisions during early embryogenesis

**DOI:** 10.1038/s41598-021-86919-3

**Published:** 2021-04-12

**Authors:** Tongqiang Fan, Youjun Huang

**Affiliations:** State Key Laboratory of Subtropical Silviculture, Zhejiang A&F University, Lin’an, Hangzhou, 311300 People’s Republic of China

**Keywords:** Embryogenesis, Stem cells

## Abstract

This study was conducted to investigate epigenetic landscape across multiple species and identify transcription factors (TFs) and their roles in controlling cell fate decision events during early embryogenesis. We made a comprehensively joint-research of chromatin accessibility of five species during embryogenesis by integration of ATAC-seq and RNA-seq datasets. Regulatory roles of candidate early embryonic TFs were investigated. Widespread accessible chromatin in early embryos overlapped with putative *cis*-regulatory sequences. Sets of cell-fate-determining TFs were identified. *YOX1*, a key cell cycle regulator, were found to homologous to clusters of TFs that are involved in neuron and epidermal cell-fate determination. Our research provides an intriguing insight into evolution of cell-fate decision during early embryogenesis among organisms.

## Introduction

An outstanding challenge of developmental biology is to explain how multi-cell organisms originate from a single cell that mature through complex dynamic processes. In complex organs, the generation of a single lineage usually involves multiple steps of cell fate decisions. Comprehensively, understanding the pathways of cell lineage differentiation during in vivo development, especially transcriptional regulatory strategies at point of cell lineage segregation, and the common characteristics of multiple organisms, in critical for directing stem cell differentiation into desired cell types and the discovery of common ground of eukaryotes. Early embryo development of the metazoans and plants comprise a sequence of cell fate decisions in which cells are guided along a pathway of restricted potential and increasing specialisation.

During mammalian early embryo development, stem cell will undergo some cell fate determined phases, including ZGA (zygote genome activation), 1-, 2-, 4-, 8-, 16-, 32-cell, early blastocyst, and late blastocyst stages, in which 8–32-cell stages exhibit first cell fate decision, and early to late blastocyst stages occur second cell fate decision^1–3^. The embryonic cell lineage of *C. elegans* has been traced from zygote to newly hatched larva^4^, in which 1-, 2-, 4-, 8-, and 16-cell stage embryos can represent early embryo cell lineage^5^. The control of the oocyte-to-embryo transition in Drosophila parallels that of other animals, however, in early *Drosophila* embryos, the master checkpoint laid on nuclear cycle from cycle 10 to 14^6^.

Compared to mammals, plants are ancient organisms, which require many centuries to obtain new organs. Shoot, root, flowers, fruits are continuously augmented to build sophisticated post-embryonic tissues. This process requires coordination of cell divisions, cell fate determination, and cell communication. Starting from fertilization, embryogenesis produces the first stem cell, and by the end of embryogenesis the zygote has transformed into a mature embryo that comprises the basic tissue types identical to any post-embryonic plant^7^. The processes of plant early embryo development include multiple stages: two cell, eight cell, 16 cell, globular, heart, torpedo, and bent stages^8^.

TFs (transcription factors) play an irreplaceable role in cell fate determination during embryogenesis^9^. The identities of cell types are also influenced by environmental signal transduction, in turn, results in the activation or inhibition of TFs^10^. Much progress has been made in understanding how core regulators such as *OCT4*, *NANOG*, and *SOX2* as well as transcriptional effectors of signaling pathways, such as *SMAD1*/*2/3/4*, and *TCF3*, control the regulatory circuity that control earliest stage of embryonic development^11^. During early embryogenesis, inner cells develop a stable regulation circuit, in which *OCT4*, *SOX2*, and *NANOG* are assigned to promote pluripotency to determine ICM (inner cell mass) cell fate^12^. In contrary, outer cells upregulate TFs such as *CDX2* and *EOMES* to promote the formation of trophectoderm-destined cells^13,14^. Regulation of trophectoderm targets by *OCT4*, *SOX2* and *NANOG* in the pluripotent lineage^15^, accompanied by the autoregulatory properties of *OCT4* and *CDX2*^16^, ensure the maintenance of lineage segregation. *SALL4*, which establishes and maintains ICM integrity by upregulating *Oct4* and Nanog expression^17^; *TEAD4*, which acts upstream regulator of *CDX2* during trophectoderm development^18^; and episomal expression of the early trophoblast TFs such as *TCFAP2C*, *GATA3*, *ELF5* or downregulation of the pluripotency factor *OCT4* can induce trophoblast cell fate determination in ES cells^19–21^; are all important supplements to the regulatory networks of the first cell fate decision.

Generally, TFs bind to open chromatin of *cis*-regulatory regions, such as promoters, and enhancers, however, a subset called ‘pioneer factor’ are dominant in their ability to engage silent, unmarked chromatin and initiate the recruitment of other factors, thereby creating a permissive state for gene activation^22,23^.

The development of a multicellular organism with its organs and tissues is a reproducible event that shows high coordination between the increase of cell mass and the diversification of cell population. These reproducibility programmes underly species cell-fate decision events^24,25^, which are implemented by gene regulatory networks (GRNs), which are basic units of molecular activity that establish and maintain functional tissues by promoting sequential, irreversible gene expression that link genes to cell lineages^26,27^. The regulation mechanisms of cell-fate decisions lack of systematic research during early embryonic development at the chromatin level, especially with regard to cross-species joint research. Here, we focused on the functions of TFs and GRNs associated with cell-fate decision events during early embryogenesis. We used publicly available ATAC-seq^28^ datasets of six species, human, mouse, *Drosophila*, *C. elegans*, *Arabidopsis*, and yeast (we termed cell cycle stage as early embryogenesis, as lifespan of yeast is short and cell-fate decision events may occur as early as cell cycle) to question whether there are common traits of TFs during early embryonic development and to investigate underlying transcriptional regulatory mechanism of cell fate control. We found many TFs match their functions of various cell fate determination by investigation of the dynamic chromatin changes and gene expression patterns of various species during different development stages. Furthermore, we quantified TFs abundance from different cells during early embryonic development. Finally, we investigated the evolutionary mechanisms underlying cell-fate determination. After data mining, we focused on *YOX1*, a key cell-cycle regulator in yeast, combined with homology, transcriptomes, and regulatory networks, the conserved roles of the homologs were found to be crucial for cell fate determination.

## Results and discussion

### Dynamic chromatin changes over developmental stages

**Figure 1:**
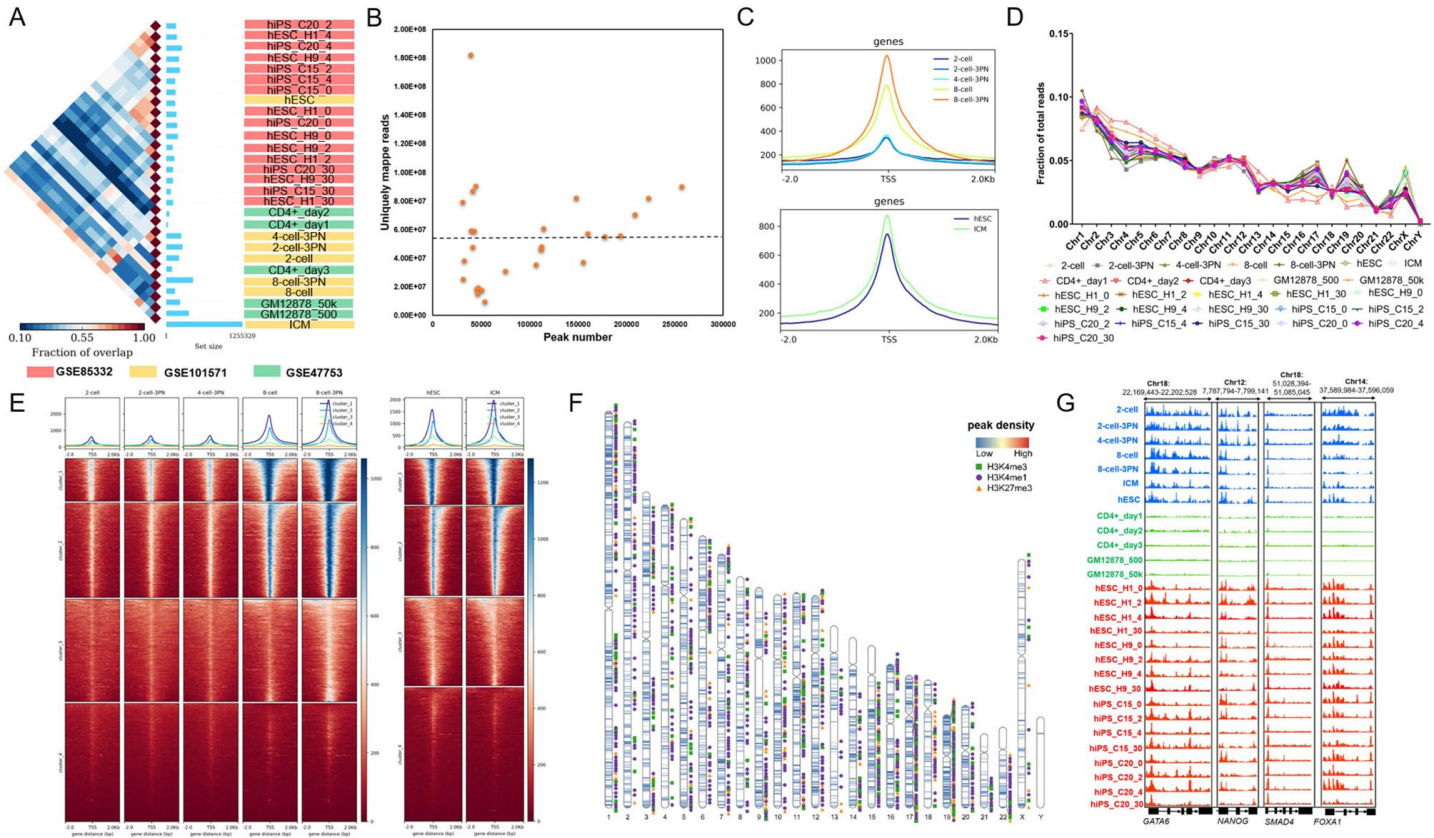
Accessible chromatin demonstrates the epigenetic dynamics across different developmental stages. (**A**) Pairwise analysis of peaks presented in ATAC-seq samples. Left, heatmap demonstrates the overlapping rate between peaks in each sample, right, histogram showing peak number identified in each sample. (**B**) Called peak counts for 28 human ATAC-seq datasets as a function of the number of uniquely mapped reads used for peak calling. (**C**) The average ATAC-seq enrichment of active genes around TSS region. The center of accessible regions was used to produce the distribution plots. The upstream and downstream regions (2 kb) of TSS are mappable. Only part of human samples was presented. Full of the enrichment plots were presented in Fig. S7. (**D**) Distribution of reads mapped to genome of human samples. (**E**) Average plots and heatmaps of ATAC-seq signals at ATAC-seq transposase hypersensitive sites (THSs). The regions in the heatmaps are ranked from highest ATAC-seq signal (top) to lowest (bottom). The cluster manually set to 4. (**F**) Distribution of peaks and DNA methylation marks in chromosomes. Peak density was calculated by average peak counts divided by peak length (kb). Only human plot was showed, the plots of other species were presented in Fig. S8 (**G**) The IGV views showing the ATAC-seq enrichment near key cell-fate-determined TFs during early embryogenesis.

Epigenome mapping is a powerful method for cataloging functional elements throughout the genome^29^, and it can provide insights into the regulatory mechanisms that underlie changes of cell fate^30^. To investigate the mechanisms underlying cell fate determination, we applied ATAC-seq datasets and standard data analysis pipeline (Figure S1) of six species (*A. thaliana*, *C. elegans*, *H. sapiens*, *M. musculus*, *D. melanogaster*, *S. cerevisiae*) as they have emerged as most appreciated models for system biological research. The detailed information about the ATAC-seq samples that we used were listed in Table S1 and S2. Firstly, we checked quality of all the raw materials, and the results showed that the insert size distribution of each ATAC-seq library displays a stereotypical 150 bp periodicity that consistent with the expected nucleosome occupancy of chromatin. However, the nucleosome occupancy of *Arabidopsis* was not so obvious, as plants have mitochondrial and chloroplast genomes, which are completely accessible to Tn5, and likely depletes Tn5 activity from the nuclear genome^31^ (Figure S2). Then, we checked the number of reads mapped to each chromosome (Fig. 1D, Fig. S5). The result showed highly similar reads distribution pattern, indicating of high sample quality. We designed a stringent computational framework to integrate all the samples from different species with unified parameters, resulting in the identification of 25,000–65,000 high-confidence, accessible peaks for *Arabidopsis*, 30,000–54,000 for *Drosophila*, 28,000–1,250,000 for human, 8000–650,000 for mouse, 2000–3500 for yeast, and 15,000–28,000 for nematode (Fig. 1A, Fig. S3). Examination of peak signals versus uniquely mapped reads revealed that the signal enrichments consistently plateau at greater sequencing depths (Fig. 1B, Fig. S4).

To investigate dynamics chromatin changes over different developmental stages of each species, we used deepTools2 software^32^. Visualization of all the ATAC-seq datasets revealed that with the developmental stages proceeding, most peaks were in promoter-TSS (transcription start site) region (Fig. 1C,E, Figs. S6, S7) indicating these binding sites were predominantly located around TSSs indicating these regions are critical for TF binding and transcription regulating. Histone modifications, function as a prerequisite for dynamic chromatin state changes allow perpetual diversification of epigenome^33^. We found that H3K4me3 and H3K27ac modifications were associated with relatively higher peak density, compared to H3K4me1 and H3K27me3 with low density (Fig. 1F, Fig. S8). Because previous studies have demonstrated that H3K4me3 and H3K27ac were commonly associated with the activation of transcription^34^ and mark spot of active enhancers^35^, respectively, however, H3K4me1 and H3K27me3 were associated with transcriptional silencing and downregulation of nearby genes^36,37^.

Taken together, these findings showed comparable open chromatin landscapes in early embryos, as early embryo samples tend to enrich more accessible signals compared to mature tissue samples.

### Chromatin accessibility extends the dictionary of *cis*-regulatory elements

**Figure 2:**
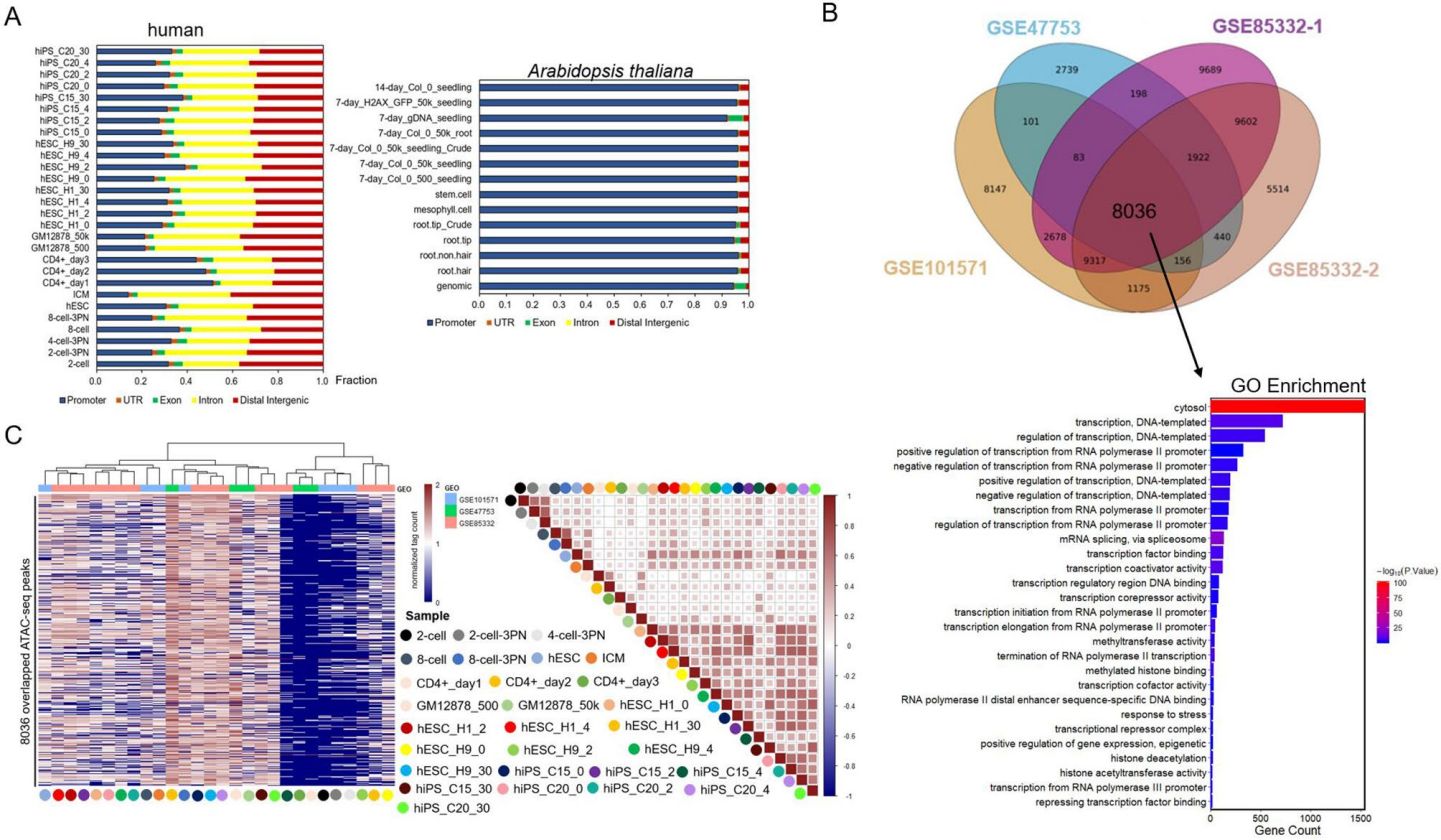
Genomic and functional annotation of accessible regions. (**A**) Genomic distributions of enriched accessible regions identified in ATAC-seq samples. THS peaks within TSS ± 3 kb are considered as promoter THS, and those not located in promoters, exons, introns, or UTRs are labeled as distal intergenic. (**B**) GO functional enrichment analysis of overlapped peaks. Upper part integrates Upset and Venn method to identify overlapping peaks across all human samples. Bottom part is a bar plot of GO enrichment of overlapping peaks. (**C**) Heatmaps showing the ATAC-seq enrichment (RPKM) (left) and the comparison of ATAC-seq signal within consensus ATAC-seq peaks by Pearson’s Coefficient Correlation algorithm. The colored bubbles represent different samples. The ATAC-seq enrichment signals were normalized by log2(FPKM + 1).

In a comparison of open chromatin among epigenomes of human, mouse, *Drosophila*, worm, *Arabidopsis*, and yeast, we found the genomic distribution of THSs in each were highly similar, as majority of the peaks were enriched in promoter regions, except for human and mouse samples (Fig. 2A, Fig. S9). However, more than 90% of THSs lie outside of transcribed regions, and the majority of these THSs were found within 3 kb upstream of TSS in all species but for human, mouse, and fruit fly. The differences in reads distribution between advanced organisms and relatively lower livings may due to the fact that transcriptional regulatory elements (TREs) in plants and microbes are generally less numerous and closer to the genes they regulate than those of advanced genomes. For example, the median distance the enhancer and the TSSs of their target genes in fruit fly was reported to be 10 kb^38^. It was also uncovered that in human T cells, 91% of the enhancers, with a median distance of 130 kb between promoters, far greater than the distance across plant and fungi genome^39^. Interestingly, compared to embryonic stem cells, more peaks were enriched in the CD4^+^T cell promoter regions. Previous research has demonstrated that more promoters marked by H3K4me3 during early phase of CD4 T cell activation, which could enhance accessible chromatin status and reinforce activation-induced upregulation of gene expression^40^. Overall, it is clear that in all species the majority of THSs are located in promoter regions, suggesting that accessible chromatin overlaps extensively with putative *cis*-regulatory sequences.

While assessment of open chromatin plays an important role in predicting regulatory element in the genome^29,41^, it does not provide direct information of functional activity. To address this issue, we asked whether shared open chromatin regions exist within all the datasets in each species, and what the role of regulatory elements that bound to these shared regions played. The results showed that there were 1082 shared peaks for *A. thaliana*, 7519 for *C. elegans*, 7970 for *D. melanogaster*, 1995 for *S. cerevisiae*, 8036 for *H. sapiens*, and 4151 for *M. musculus*. Functional enrichment annotation of these overlapped peaks shows that they were almost enriched in transcription and regulation of transcription (Fig. 2B, Fig. S10), indicating that majority of TFs that bound to accessible regions are involved in basal transcriptional activity. Then, we evaluated the ATAC-seq enrichment profiles of all samples in each species, the results showed differential ATAC-seq signals between different tissues/strains or developmental stages, indicating tissue or developmental heterogeneity (Fig. 2C, Fig. S11), except for *C. elegans*, which showed comparable signals in different developmental stages. And the high correlation of ATAC-seq signal between each of the sample (Fig. 2C, Fig. S11) demonstrates the high reproducibility.

Collectively, these results suggest that TREs tend to be focused near the promoter rather than at more distal regions. The hypothesis implicit that open chromatin site near a TSS reflect TREs that regulate TSS rather than more distal regions, and that promoter-binding upstream elements contribute the majority of regulatory effects. And the TREs bound to promoters generally are in basal transcription and regulation of transcription function. Interestingly, these assumptions were previously validated in wet lab showing that an upstream fragment of several kilobases is capable of recapitulating native transcription patterns^42–44^, which are consistent to our findings that upstream THSs are the most abundant category of accessible chromatin sites.

Taken together, these data indicated that THSs in animal and plant genomes showed largely comparable landscapes and *cis*-regulatory elements that bound to open chromatin regions mainly play roles in transcription and regulation of transcription.

### Identification of cell-fate TFs during early embryogenesis

**Figure 3:**
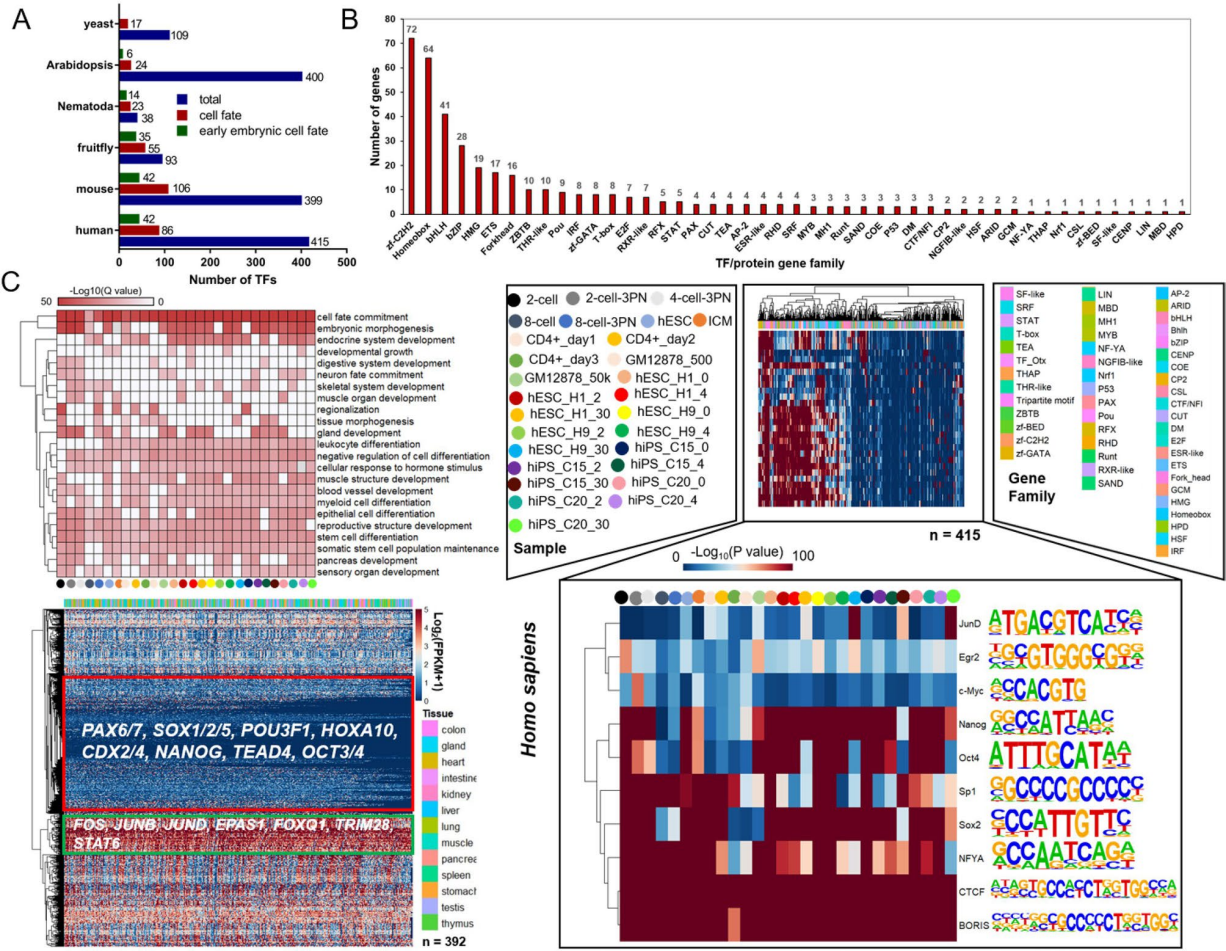
Classification and functional annotation of TFs identified from ATAC-seq samples. (**A**) Classification of TFs that identified from all ATAC-seq samples. (**B**) Gene family classification of the identified TFs in human. The gene family information were collected from JASPAR (http://jaspar.genereg.net/). The information of other species were listed in Fig. S12. (**C**) Functional and expression pattern analysis of TFs identified from ATAC-seq samples. Right part, annotation of all TFs and randomly selected 10 for motif analysis. Top left, heatmaps showing the top 20 enriched GO terms of all TFs using Metascape enrichment. Bottom left, expression patterns of all TFs across different tissues. The expression profiles were obtained from ENCODE database (https://www.encodeproject.org/). And the raw expression matrices were normalized by log2(FPKM + 1).

We applied HOMER *findmotif* to determine what TFs that bound to these open chromatin regions. And 400 for *A. thaliana*, 38 for *C. elegans*, 93 for *D. melanogaster*, 109 for *S. cerevisiae*, 414 for *H. sapiens*, and 398 for *M. musculus* were identified (Fig. 3A, Table S3). Gene family classifications showed that majority of the identified TFs were enriched in Homeobox and C2H2 zinc finger family (Fig. 3B, Fig. S12). Motif discovery indicated that, *PIF4*, *PCF*, *BIM1*, and *JKD* genes were highly enriched for root and seedlings in *Arabidopsis*, *elf-1*, *hlh-30*, *dpl-1*, *eor-1*, *pha-4*, and *pqm-1* were highly enriched during larva development in *C. elegans*, *zld*, *Dref*, and *Trl* for *D. melanogaster* during nuclear cycle period, *ABF1*, *REB1*, *AZF1*, *OPI1*, and *RSC3* for different strains of *S. cerevisiae*, *CTCF*, *BORIS*, *SOX2*, *NFYA*, *SP1*, *OCT4*, and *NANOG* for *H. sapiens* during embryonic stem cell development, and *JunB*, *Batf*, *Nanog*, and *AP-1* for *M. musculus* during induced pluripotent stem cell development (Fig. 3C, Fig. S13). To investigate the functions of these TFs, we performed functional GO (gene ontology) analysis. The results showed that these TFs were almost involved in transcription and regulation of transcription. Interestingly, we also found that some TFs were TFs involved in cell fate decisions (cell fate commitment, and cell fate specification) (Fig. 3C, Fig. S13).

To investigate the expression patterns of these TFs across different tissues or strains, we collected expression profiles from public databases. For *Arabidopsis*, the TFs such as *GATA1*, *TCP3*, *CDF3*, *PIF4*, *CCA1*, *LHY*, *SPL1*, and *MYB38* were highly expressed in different mature tissues, and *ABI5*, *WUS*, *HB5*, *WIP5*, and *SHP1* were lowly expressed even unexpressed (Figure S13), as these genes are involved in nuclear cycle or early embryonic development^8,45–48^. For *C. elegans*, all identified TFs were deemed to differentially expressed across multiple strains (Figure S13). For *Drosophila*, some TFs such as *Kr*, *bcd*, *zen*, *cad*, and *twi* hardly expressed in various mature tissues, as these TFs previously supposed to play major role in early embryonic development of *Drosophila*^49–53^. For *S. cerevisiae*, it is obvious that the identified TFs were differentially expressed across all yeast strains and were higher in strain w303a than other strains (Figure S13). For *H. sapiens*, we found some TFs, *PAX6*, *SOX2*, *POU3F1*, *HOXA10*, *CDX2*, *NANOG*, *TEAD4*, and *OCT4* were scarcely expressed across mature tissues, because they function in early stem cell development^54–57^. For *M. musculus*, we also found some TFs, *Cdx2*, *Oct4*, *Eomes*, *Esrrb*, *Gsc*, and *Nanog*, were scarcely expressed in mature tissues, as these TFs constitute an important reservoir for early embryonic development^57–59^.

Additionally, we found a set of TF complexes which were pertinent to cell proliferation (*Oct4*::*Sox17*, promotes cell development and differentiation^60^, and *OCT4*-*SOX2*-*TCF*-*NANOG*, forms core regulatory circuitry of ES cells, critical for pluripotency and self-renewal^61^), cell differentiation (RAR/RXR, triggers pluripotent cell differentiation^62^, *NF1*::*FOXA1*, mediate gene expression and cell differentiation in prostate^63^), tumorigenesis, and immunogenesis (Figure S14), demonstrating that even in early embryos, these TFs that involved in oncogenesis, and tumor suppress are also expressed to maintain normal cell divisions and differentiation of early embryo.

To further validate some key TFs that function in early embryos, we visualized the ATAC-seq signal enrichment near them across all samples in each species using IGV (Integrative Genomics Viewer). The results showed that for the developmentally regulated genes, such as *GATA6*, *NANOG*, *SMAD4*, and *FOXA1* in human, were found elevated ATAC-seq enrichment at annotated or putative enhancers and promoters during embryonic development instead of in mature cells (Fig. 1G). For TFs such as, *Oct4*, *Sox4*, *Eomes*, and *Gata4*, we also observed increased signals during mouse embryonic development (Figure S15), which comparable to *Su(H)*, *zen*, *Abd-B*, and *twi* during *Drosophila* nuclear cycle (Fig. S15). However, for *WUS*, *ATML1*, *JKD*, and *KAN* in *Arabidopsis*, they showed distinct signal intensity over different tissues or under different treatments (Figure S15). Nevertheless, *FKH1*, *STE12*, *MSN2*, and *DIG1* in *S. cerevisiae* showed comparable signal intensity over different strains (Figure S15).

Overall, by integrating the information of cell-fate-determined TFs and the transcriptomes, we delineated that these cell-type-specific TFs showed high tissue or developmental heterogeneity.

### Regulatory networks of cell-fate decision in early embryo

**Figure 4:**
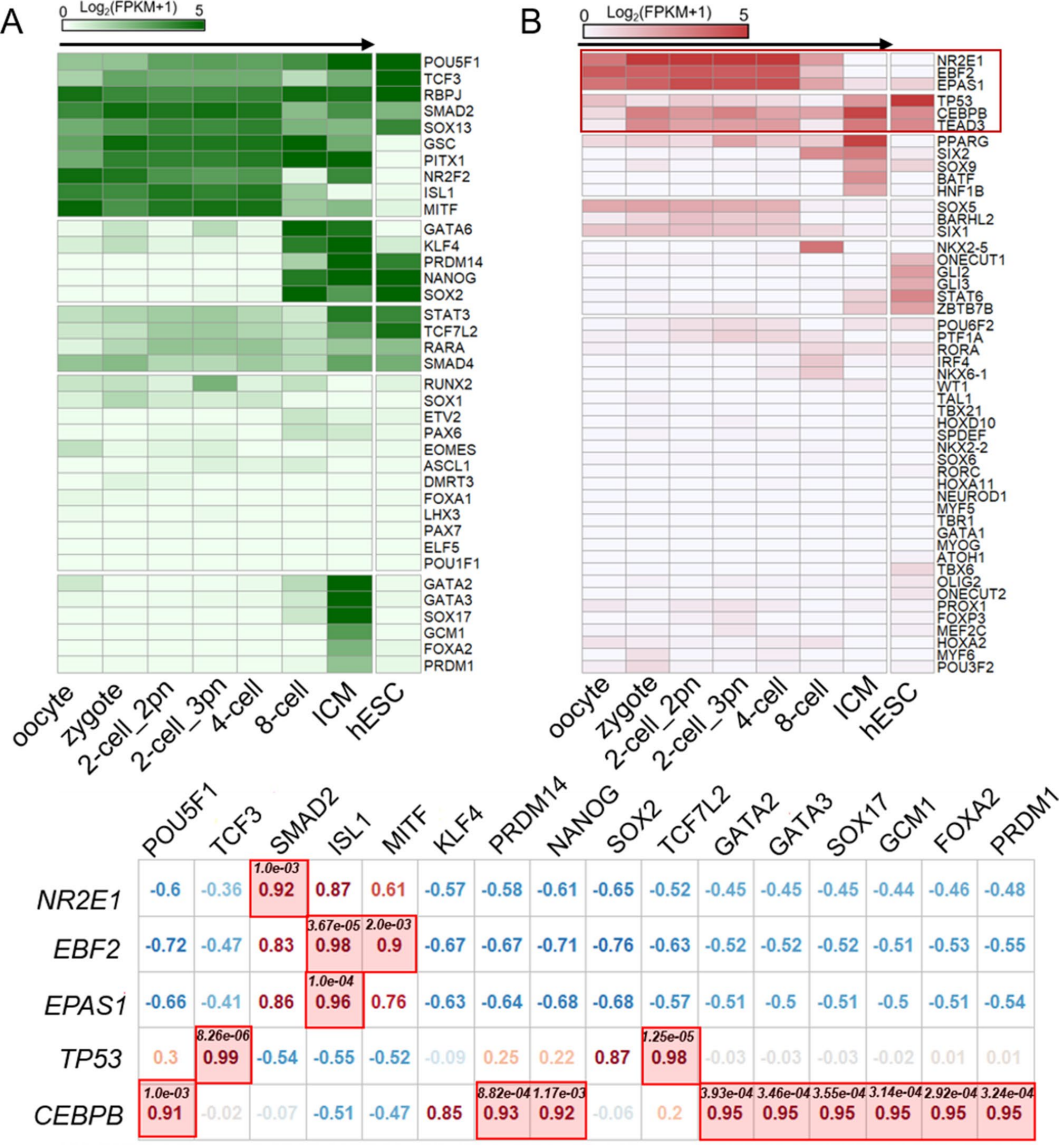
Expression profiles of TFs involved in early cell fate determination. (**A**) The expression patterns of stat-of-the-art early cell fate determined TFs during early embryonic development. (**B**) Exhibiting the expression profiles of cell-fate TFs during early embryogenesis. Bottom, showing the correlations of expression pattern between public-accepted TFs of early cell-fate determination and some that we identified in this research. The normalized expression matrices were collected from NCBI database from GSE101571 accession.

Cell fate decisions play a key role in crucial processes such as tissue repair, immune response, or embryonic development^64–66^. Here, we identified numerous TFs that are involved in cell fate control (Fig. 4, Fig. S16). For each species, using public expression profiles of early embryonic development, we have not only verified high expression values of some previously widely accepted early embryonic TFs (Fig. 4A), but also found some cell-fate determining TFs that were highly expressed that previously unreported during early embryonic development (Fig. 4B, Fig. S16), indicating they may play roles in early embryos. However, we also found some TFs previously reported to play major roles during early embryogenesis in *Drosophila*, had a low expression pattern during early embryonic stage, such as *pnr*^67^, *vnd*^68^, and *Ubx*^69^ (Figure S16). The expression profiles of some previously unreported TFs that function in early embryogenesis were also have high correlations with some early embryonic TFs, such as *Jra*, *Blimp-1*, *hth*, and *Tk* in *Drosophila*, *NR2E1*, *EBF2*, *EPAS1*, *TP53*, and *CEBPB* in human.

To comprehensively resolve the mystery of regulatory mechanisms of cell fate control during early embryogenesis, we combined cell-fate TFs of six species to construct TF regulatory network to predict the regulatory circuit based on their homology relationships (Figure S17). And, we investigated some homolog TFs in other five species for human TFs (Table S4). Surprisingly, these homolog TFs are also previously reported to be involved in cell fate control during early embryonic development.

**Figure 5:**
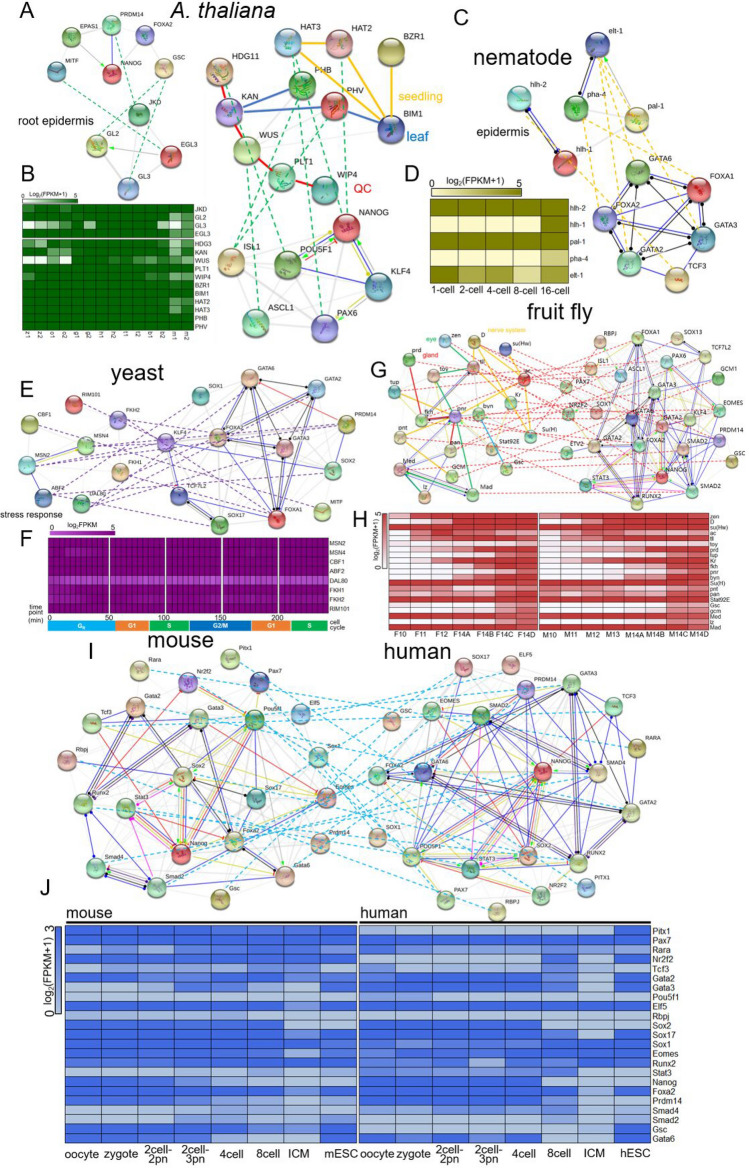
Transcriptional regulatory networks underlying early embryonic cell fate determinations. (**A**) TFs mainly play roles in *Arabidopsis* root epidermis, seedling, leaf, and QC (quiescent center) development, orange, blue, and red lines indicated interactions that control seedling, leaf, and QC cell fates, respectively. Green dashed lines represent homology relationship between *Arabidopsis* TFs and human TFs. (**B**) Expression profiles of these *Arabidopsis* TFs during early embryogenesis. The x-axis represents different stages of *Arabidopsis* early embryonic development, which were annotated in Figure S16. (**C**) TFs determine the cell fate of *C. elegans* epidermis. Orange dashed lines represent homology relationship between nematode TFs and human TFs. (**D**) Expression patterns of these five TFs during *C. elegans* early embryogenesis. (**E**) TFs involved in pattern formation of stress responses in yeast. Purple dashed lines represent homology relationship between yeast TFs and human TFs. (**F**) Expression patterns of these eight TFs during *S. cerevisiae* cell cycle. (**G**) TFs participate in cell fate determination of eye, gland, and nerve system in fruit fly. Orange lines demonstrate TF interactions that control nerve system, red lines demonstrate TF interactions that control gland development, green lines demonstrate TF interactions that control eye cell fate. The red dashed lines represent homology relationship between fruit fly TFs and human TFs. (**H**) Expression patterns of these 21 TFs during *D. melanogaster* cell cycle. (**I**) Regulatory circuits determine mouse and human early embryonic cell fate. (**J**) Expression patterns of these TFs during early embryogenesis of mouse and human. The interaction relationships were all predicted by STRING database. The normalized expression profiles were collected from NCBI database accessions of GSE101571 (human), GSE66582 (mouse), GSE25180 (fruit fly), GSE77944 (nematode), GSE123010 (*Arabidopsis*), and GSE104904 (yeast).

For *Arabidopsis*, we analyzed several key TFs, which play key roles during root epidermis patterning, seeding, leaf, and QC (quiescent center) development in details (Fig. 5A). Four cell-fate-determining TFs, *JKD*, *GL2*, *GL3*, and *EGL3*, which are homolog to *PRDM14*, *GSC*, and *MITF* in human, respectively, are indispensable for controlling the patterns of epidermis in the *Arabidopsis* root meristem^70^. *HAT3*, homologs to *PAX6* and *NANOG*, combined with *HAT2*, *BZR1*, and *BIM1* to promote seedling development^70–72^. We assume that *BIM1* may play a role as a signal integrator to integrate signals from *HAT2*, *HAT3*, and *BZR1* to promote seedling development (Fig. 5A). Another cluster of TFs, *KAN*, *PHB*, *PHV*, and *BIM1*, in which *PHB* and *PHV* are homolog to *ISL1*, contribute to promote *Arabidopsis* leaf development and leaf adaxial polarity^73,74^. We hypothesize that *BIM1* may function as downstream target genes of *PHV* to regulate leaf development (Fig. 5A). The last cluster TFs that we found have homologs of human early embryonic TFs are *HDG11*, *KAN*, *WUS*, *PLT1*, and *WIP4*, in which *HDG11* and *WUS* are homolog to *POU5F1* and *ASCL1*, respectively. Previous studies have demonstrated that *WUS*, *PLT1*, and *WIP4* all contribute to the cell-fate determination of QC^47,75,76^. Therefore, we conject that *HDG11* and *KAN* may function as upstream target genes of *WUS* to form the *HDG11*-*KAN*-*WUS*-*PLT1*-*WIP4* complex to control the cell-fate determination of QC. The expression value of all cell-fate-determined TFs mentioned above all keep high levels during *Arabidopsis* early embryonic development (Fig. 5B), indicating they may play major roles in *Arabidopsis* early embryos.

For *C. elegans*, we identified several early embryonic TFs, *hlh-2*, *pha-4*, *elt-1*, *hlh-1*, and *pal-1*, in which the former three are homolog to *TCF3*, *FOXA1/2*, and *GATA2/3/6*, respectively (Fig. 5C). Previous demonstrated *elt-1* and *pal-1* are critical for the specification of epidermal cell fates^77,78^, furthermore, in our study, we presumed that *hlh-2* and *hlh-1* may act as upstream target TFs of *pal-1*, and *pha-4* functions as binding protein of *elt-1*, and these five TFs function together to control epidermal cell fate. The expression patterns of these five TFs showed highest levels at 16-cell stage (Fig. 5D), indicating 16-cell stage may be a critical timepoint for epidermal cell fate determination.

As the propagating method of *S. cerevisiae* is budding reproduction without embryo development, we analogously regarded the cell cycle period as embryonic development stage. We identified several TFs, *RIM101*, *FKH1*, *FKH2*, *MSN2*, *MSN4*, *ABF2*, *DAL80*, and *CBF1*, which homolog to *FOXA1*, *FOXA2*, *KLF4*, *PRDM14*, *SOX2*, *SOX17*, *GATA2/3/6*, and *MITF*, respectively (Fig. 5E). Previous studies showed that these TFs were all involved in stress responses^79–81^. So, we proposed a regulatory circuit that regulate the progressive process of stress responses based on their interaction relationships (Fig. 5E). And, we noticed these TFs kept high expression values during the full stages of cell cycle (Fig. 5F), indicating that yeast is susceptible to external or internal damages, TFs that regulate the defense systems need to be constantly functioning.

For *D. melanogaster*, we identified several clusters of TFs that involved in eye, gland, and nerve system cell fate determination (Fig. 5G). Firstly, a cluster of TFs, *zen*, *tll*, *toy*, *pnr*, *Mad*, *Med*, and *lz*, which homolog to *NANOG*, *NR2F2*, *PAX6*, *GATA2/3/6*, *SMAD2*, and *SMAD4*, respectively, were previously reported to be involved in pattern formation of eye cell fate^82–84^, we proposed the model for eye cell fate decisions, *lz*-*Med*-*Mad*-*pnr*-*toy*-*tll*-*zen*, in which *lz* bound to *Med*, and *Med* bound to *Mad*, to promote the expression of *Mad*, as a research have shown that *lz* encodes a TF involved in prepatterning photoreceptor precursors in the *Drosophila* eye^85^. Then, *prd*, *fkh*, *pnr*, and *pan*, in which the latter three are homolog to *FOXA1/2*, *GATA2/3/6*, *TCF7L2*, and *SOX13*, respectively, were demonstrated to be indispensable for gland cell fate determination^86–90^, we proposed a regulatory model, *pan*-*pnr*-*fkh*-*prd*, in which pnr served as a binding protein, which bound to *fkh*, and prd may function as terminal target gene. Overall, these TFs function together to promote gland cell fate determination and cell development. Thirdly, *su(Hw)*, *ac*, *Su(H)*, *Kr*, *tll*, *D*, *pnr*, *pnt*, and *gcm*, homolog to *PRDM14*, *ASCL1*, *RBPJ*, *KLF4*, *NR2F2*, and *SOX1/2*, were reported to constitute important reservoirs for nervous system cell fate decisions^91–93^. Based on the interactions of these TFs, we proposed a regulatory circuit, which is required for the cell fate control and development of nervous system (Fig. 5G). Expression profiles of these TFs showed that they started to play functions from nuclear cycle stage 14, regardless of some had high expression values from the very beginning of the nuclear cycle (Fig. 5H).

We have identified 24 human homologue TFs in mouse (Fig. 5I). Similar to humans, these TFs also function to determine cell fate or promote cell development during early embryonic developmental stage. Furthermore, the interactions of TFs were almost the same, and the expression patterns of these TFs were slightly different (Fig. 5J).

Taking together, by integrating the information about *cis*-regulatory elements and the transcriptomes, we scratched the surface of cell-fate-determining regulatory networks during early embryonic development that is orchestrated by a set of TFs and their targets.

### Evolution of cell fate decision in early embryos

**Figure 6:**
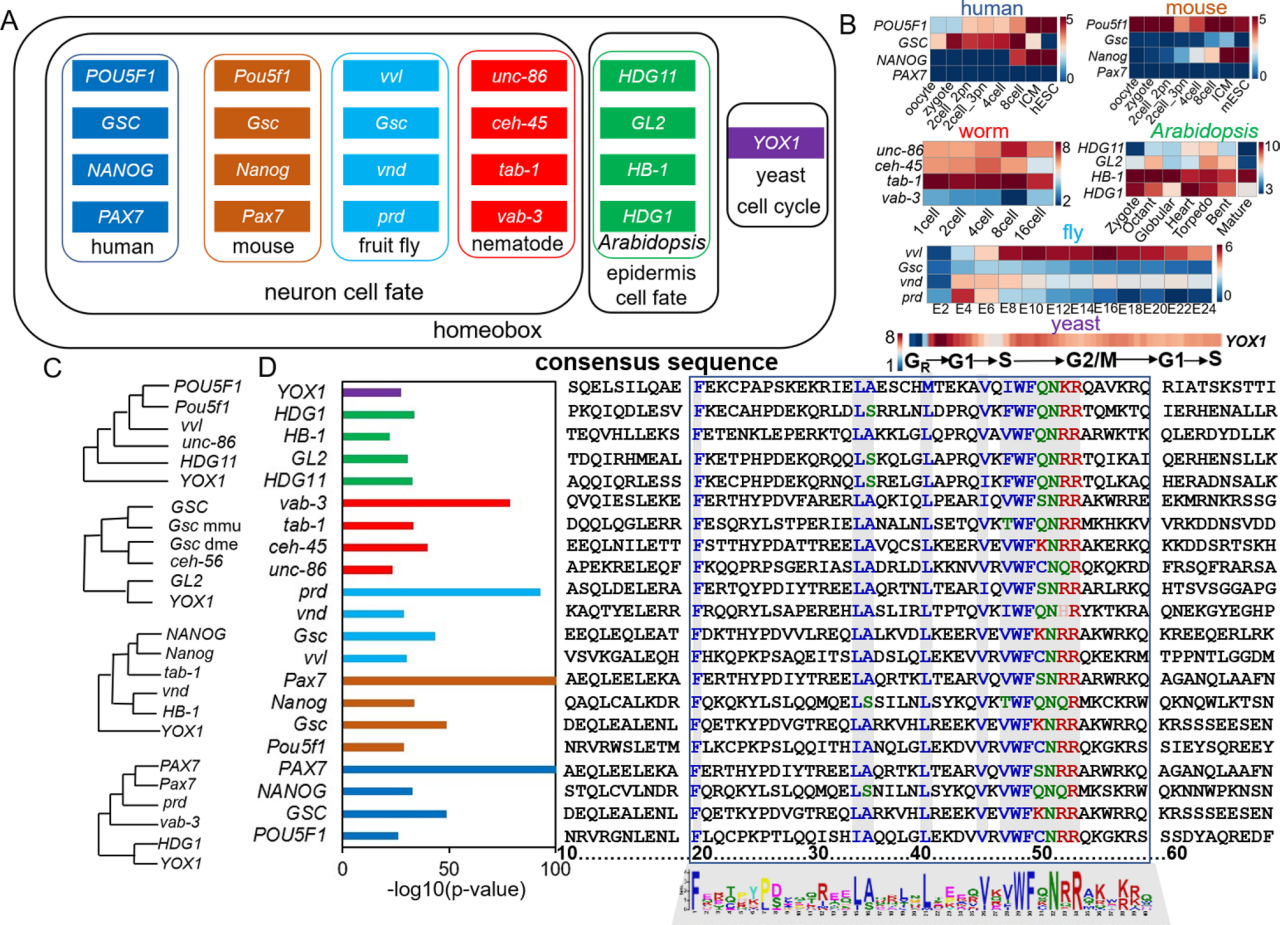
Evolution basis of members of homeobox TFs and their roles in cell fate decisions. (**A**) Key cell cycle transition TFs, YOX1, and its orthologs in five other species play roles in cell fate decision. Homologous TFs of YOX1 in human, mouse, fruit fly and nematode are involved in neuron cell fate determination, while in Arabidopsis, control epidermis cell fate. All of the TFs are members of homeobox family. (**B**) Heat maps showing the expression patterns of the cell-fate TFs during early embryogenesis. E2–24 representing 2–24 embryo stages. The normalized expression matrices were collected from NCBI GSE101571 (human), GSE66582 (mouse), GSE77944 (nematode), GSE123010 (*Arabidopsis*), modENCODE (fruitfly), and GSE104904 (yeast). (**C**) Phylogenetic trees of the homeobox TFs across species. Neighbor joining and 500 bootstrap runs were carried out using the protein sequence. (**D**) Multiple alignment of homeobox TFs across species. The left bar showing the adjusted p value of motif corresponding to each TFs. The right part representing the consensus sequences alignment of the corresponding TFs. The standard numbering of a typical HD (homeodomain) with 60 residues starting from 10 (the upstream 9 residues were not shown) is given at the bottom, and the blue-lined box denote the conserved regions from 20 to 59 of HDs.

To further investigate the evolution characteristics of cell fate decisions, we focused on *YOX1*, a key G1/S transition regulator in yeast. We discovered a cluster of homeobox TFs in human, mouse, fruit fly, nematode, and *Arabidopsis* that orthologous to *YOX1*. The orthologous TFs in former four species are involved in neuron cell fate determination, while in *Arabidopsis* promote epidermis cell fate decision (Fig. 6A). Expression profiles of these TFs showed relatively high expression values during early stage of embryonic development, indicating cell fate decision events may occur during early embryogenesis. While distinct expression patterns across species during embryonic development might be a cue for the differences in the determination of different cell fates (Fig. 6B). Phylogenetic analysis of protein sequence of these homologous TFs indicated that TFs in yeast and Arabidopsis are more ancient in evolution, compared to that of advanced organisms (human, mouse, fruit fly, and worm) (Fig. 6C). Because yeast and Arabidopsis have experienced an ancient whole-genome duplication event^94^. We further investigated whether consensus sequence of these TFs shared. And the result showed a motif in homeobox domain from residues 20 to 59 was conserved, indicating the critical functions in cell fate decision events (Fig. 6D).

**Figure 7:**
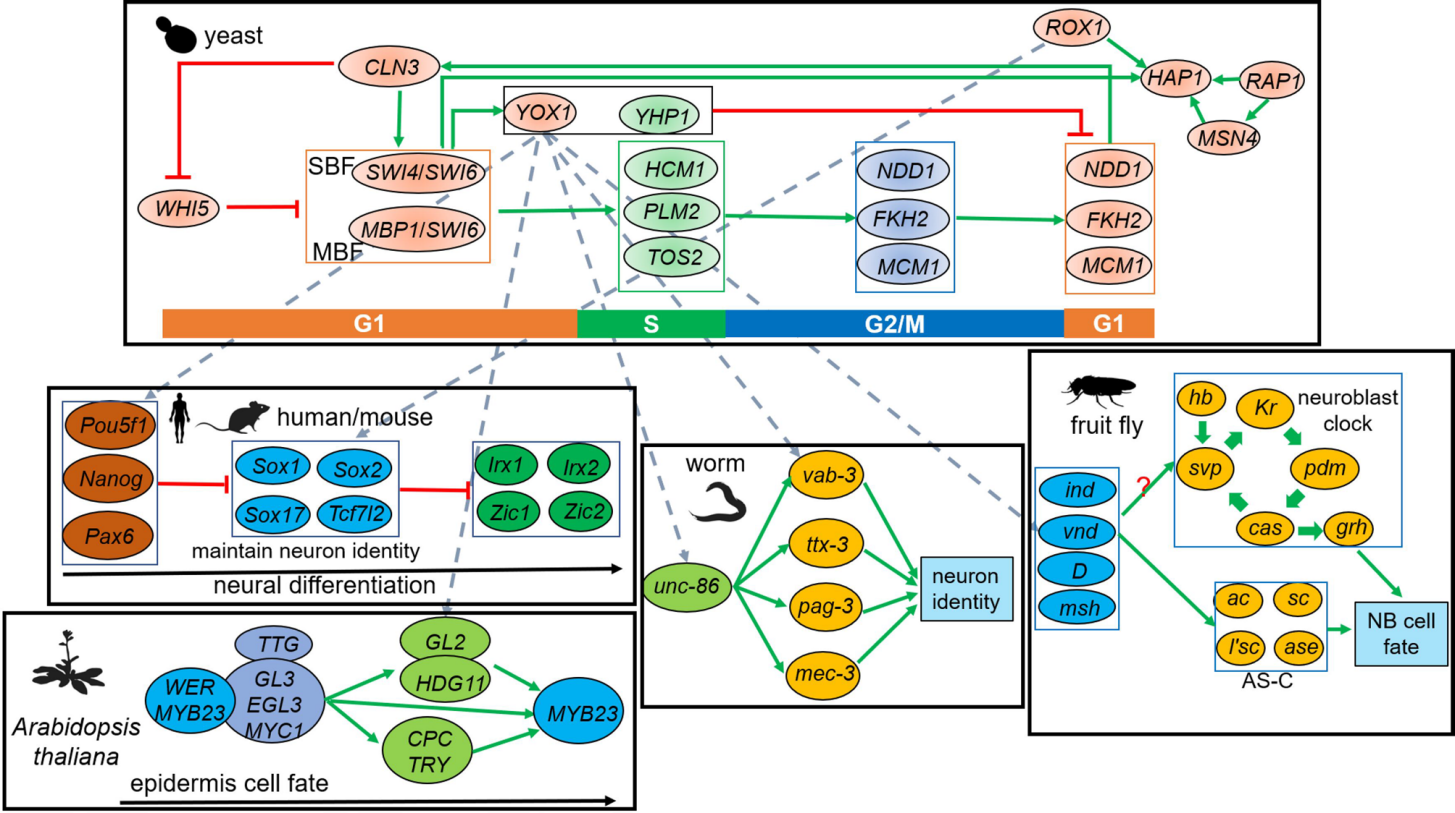
An integrated regulatory network model for the control of cell fate decision events during early stage of embryogenesis/cell cycle. This diagram depicts the regulatory interactions of cell fate determination occurring early embryogenesis/cell cycle. networks in black lined boxes represent regulatory interactions corresponding to each species. Transcription factors are denoted by ovals. Dashed grey lines represent orthologs. Green solid lines represent positive regulation and red lines represent negative regulation. Genes in colored boxes represent gene complexes/interactions. The question mark represents the regulatory relationship was unknown. *AS-C* achaete–scute complex.

Then, we constructed an integrated network to investigate the transcriptional regulatory functions of above TFs in cell fate decision events (Fig. 7). *YOX1*, a TF expressed in mid-G1 through early S phage, interplays with S-specific TF—*YHP1*, function as transcriptional repressor to negatively regulate *MCM1*-*FKH2*-*NDD1*-mediated G2/M-G1 transition during cell cycle progression^95^. Recently, a research has reported that *ROX1* is in promotion of *RAP1*-*HAP1*-*MSN4* module, which is an important branch for G2/M to G1 phase transition in yeast^96^. By homology analysis, we found *YOX1* was homologous to three TFs (*Pou5f1*, *Nanog*, and *Pax6*) in mouse, which were previously reported to be involved in restriction of a cluster of neuron identity maintainers (*Sox1*, *Sox2*, *Sox17*, and *Tcf7l2*), which were homologous to *ROX1*^97,98^. These maintainers, in turn, suppress the expression of neural differentiation effectors, including *Irx1*, *Irx2*, *Zic1*, and *Zic2*^99–101^. Nevertheless, the ortholog of *YOX1* in worm, *unc-86* were involved in activation of and interplay with several TFs, including *vab-3*, *ttx-3*, and *mec-3*, to define neuron identity^102,103^. For fruit fly, *YOX1* was orthologous to *vnd*, which were reported to interplay with *ind*, *D* (*Dichaete*), and *msh* to regulate neuroblast cell fate^104^. There are two models were proposed to regulate neuroblast cell fate determination, achaete–scute complex^105^ and ‘neuroblast clock’^106^, in which former one acts as proneural cluster and was activated by *vnd* to promote neuroblast formation^107^. We hypothesized that interactions of *vnd*, *ind*, *D*, and *msh* may also positive regulate neuroblast clock model in the manner as achaete–scute complex. Meanwhile, homologs of *YOX1* in *Arabidopsis*, *GL2* and *HDG11*, interact with each other, play an intermediate role of a positive feedback loop to promote epidermis cell fate determination^108^. *GL2* was positively regulated by upstream complex, called *WER*-*GL3*/*EGL3*-*TTG* transcriptional complex^109,110^, in turn, leading to the activation of downstream target gene *MYB23*^109^. Then, *MYB23* interact with *WER*-*GL3*/*EGL3*-*TTG* complex to form a positively regulatory loop^110^.

Above all, different (epidermis) or similar (neuron) cell fate decision events among different species not only depend on sequence characteristics and expression patterns of TFs but also the roles they played in regulatory networks. And conserved motifs may contribute to their conserved functions in different species.

## Methods

### ATAC-seq data acquisition

The raw ATAC-seq datasets were collected from NCBI Gene Expression Omnibus (GEO). We selected data from *Arabidopsis thaliana*, *Drosophila melanogaster*, *Homo sapiens*, *Mus musculus*, *Saccharomyces cerevisiae*, and *Caenorhabditis elegans*, as these species are well-annotated models for transcriptional regulation researches. For *Arabidopsis* samples, ATAC-seq data were collected from GSE101940^111^, GSE89346^112^, GSE101482^113^, and GSE85203^31^. For *Drosophila* samples, ATAC-seq data were collected from GSE104957^114^ and GSE83851^115^. For human samples, ATAC-seq data were collected from GSE101571^116^, GSE85332^117^, and GSE47753^28^. For mouse samples, ATAC-seq data were collected from GSE110264^118^, GSE79230^119^, GSE82010^120^, and GSE67298^121^. For yeast samples, ATAC-seq data were collected from GSE111815^122^, GSE101290^123^, and GSE66386^124^. For worm samples, ATAC-seq data were collected from GSE114439^125^ and GSE98758^126^.

### ATAC-seq data analysis

The raw ATAC-seq datasets from six difference species (human, mouse, *A. thaliana*, fruitfly, *C. elegans*, and yeast) were trimmed via trim-galore (http://www.bioinformatics.babraham.ac.uk/projects/trim_galore/), with parameters − q 20 − phred33 − nextera − length 20 − e 0.1 − stringency 3. Then the clean reads were quality-controlled by FastQC (v0.11.7, https://www.bioinformatics.babraham.ac.uk/projects/fastqc/) and MultiQC v 1.5^127^. After quality control, the clean reads from all samples were mapped to corresponding reference genome (hg38, mm10, TAIR10, BDGP6, IRGSP-1.0, and R64-1-1). For paired-end reads longer than 50 bp, bowtie2 v 2.3.4.2^128^ was applied with parameter—very-sensitive—maxins 2000, conversely, single-end reads were set to—very-sensitive. Paired-ends short or equal to 50 bp, we adopt bowtie v 1.2.2^129^ with − X 2000 − m 1 parameters to allow up to 2 kb fragments to align and only uniquely mapped reads to retain^28^. All unmapped, MAPQ < 30, and PCR duplicates were removed (samtools − F 0 × 4; samtools view q 30; sambamba markdup). Bam files were then converted to bed files and shifted using a custom shell script to reflect a 4 bp increase on the plus strand and a 5 bp decrease on the minus strand as recommended by^28^. Replicate samples were merged by samtools merge^130^.

### Genomic tracks generation

For normalization and visualization, the sorted, filtered and merged .bam files from each sample were converted to bigwig format using bamCoverage utility in deepTools v3.3.0^32^ with parameters –binSize 1 –ignoreDuplicates –skipNonCoveredRegions –normalizeUsing RPKM. The normalized ATAC-seq signal for a scaled region representing each of the genes in our gene subsets plus/minus 2 kb were compiled and plotted using the computeMatrix and plotHeatmap programs from deepTools package. All genomic track visualization was performed using Integrative Genomics Viewer (IGV) v2.4.16^131^.

### Peak calling

Peak calling on ATAC-seq data was performed using MACS2^132^ *callpeak* with parameters -g (tair10: 1.1e8, dm6: 1.4e8, hg38: 2.8e9, IRGSP-1.0: 3.7e8, mm10: 2.5e9, sacCer3: 1.2e7) − q 0.05 − extsize 200 − nomodel − shift − 100 − nolambda − keep-dup all. These parameters set a smoothing window of 200 bp between peaks before they are merged into a single peak and allow identification of variable length peaks, respectively.

### Expression and correlation of overlapped accessible regions

After peak calling, we summarized the peaks called from each species by Intervene^133^. We counted the number of reads that were enriched in overlapped peak regions by using featureCounts^134^. Peak counts were normalized to log_10_(FPKM + 1). Heatmaps of the expression of overlapped peaks were plotted to show differentially expressed peaks in all samples of each species. The count matrix of all the ATAC samples in six species was used to calculate and visualize the Spearman correlation for every sample pair by corrplot^135^ package in R.

### Peak distribution and functional enrichment annotation

We randomly selected 10,000 peaks and histone modification sites (H3K4me1, H3K4me2, H3K4me3, H3K27ac, H3K27me3, H3K36me3) in all samples of each species to show the distribution patterns of peaks in chromosomes by RIdeogram^136^. The UCSC genomic annotation was used to associate peaks with different genomic regions. Then we called the annotatePeak function from the R/Bioconductor ChIPseeker^137^ package for genomic annotation. Promoters were considered to be ± 3 kb from TSS and all the regions that did not fall within exons, introns, UTRs or promoters were classified as distal intergenic regions. The annotated peaks from ChIPseeker above were functionally enriched by compareCluster function from clusterProfiler^138^ package with default parameters.

### Transcript factor motif discovery and gene ontology

The peaks generated from ATAC-seq datasets were used for de novo motif analysis using HOMER v4.10^139^ against the JASPAR, DMMPMM, Yeast, AthaMap, and Homer databases with parameters − size 400 − len 8,10,12. De novo motifs were retained if the p value < 0.01 and (< percent of target >/< percent of background >) > 1.0. Gene Ontology enrichment for these motifs/transcription factors was performed using Metascape^140^. Those GO terms had a false discovery rate (FDR) of 0.05 or less were considered significant.

### Transcriptional regulatory network construction

To explore the transcriptional regulatory basis of six species, we used BLAST to find ortholog genes of human TFs that play cell-fate-choice function, after which we used Cytoscape to construct a comprehensive network of six species. The regulatory relationships of different TFs were predicted based on STRING and TF2Network databases.

## Conclusion

Study of the cell-fate decision across multiple species is still a long way to go, and epigenomic research seems to contribute to some extent. The findings in this study proposed possible molecules for further research of cell-fate determination. We speculate that both the TFs and motifs identified in the integration analysis of this study can be further investigated. Furthermore, the findings presented herein can be correlated with single-cell strategies, such as single-cell RNA-seq and single-cell ATAC-seq in order to uncover the mysterious veil of the evolutionary basis of cell-fate decision events.

## Supplementary Information

Supplementary Information
