# Supplementary material for: Accessible chromatin reveals regulatory mechanisms underlying cell fate decisions during early embryogenesis
Source: Sci Rep. 2021 Apr 12;11:7896. doi: 10.1038/s41598-021-86919-3 (PMC8042068; doi:10.1038/s41598-021-86919-3)
Supplement: Supplementary file 1 — Supplementary Information [file 41598_2021_86919_MOESM1_ESM.pdf]

Title: Accessible chromatin reveals regulatory mechanisms underlying cell fate decisions during early embryogenesis

Tongqiang Fan[1], Youjun Huang[1,*]

Supplementary Tables

Supplementary Table S1. Basic information of ATAC-seq samples

human

GEO	Sample	Characteristics
**GSE85332**	hiPSC_C15_0_rep1	cell type: hiPS cell; cell line: C15; differentiation time (days): 0
	hiPSC_C15_0_rep2	cell type: hiPS cell; cell line: C15; differentiation time (days): 0
	hiPSC_C15_2_rep1	cell type: hiPS-derived cells; cell line: C15; differentiation time (days): 2
	hiPSC_C15_2_rep2	cell type: hiPS-derived cells; cell line: C15; differentiation time (days): 2
	hiPSC_C15_4_rep1	cell type: hiPS-derived cells; cell line: C15; differentiation time (days): 4
	hiPSC_C15_4_rep2	cell type: hiPS-derived cells; cell line: C15; differentiation time (days): 4
	hiPSC_C15_30_rep1	cell type: hiPS-derived cells; cell line: C15; differentiation time (days): 30
	hiPSC_C15_30_rep2	cell type: hiPS-derived cells; cell line: C15; differentiation time (days): 30
	hiPSC_C20_0_rep1	cell type: hiPS cell; cell line: C20; differentiation time (days): 0
	hiPSC_C20_0_rep2	cell type: hiPS cell; cell line: C20; differentiation time (days): 0
	hiPSC_C20_2_rep1	cell type: hiPS-derived cells; cell line: C20; differentiation time (days): 2
	hiPSC_C20_2_rep2	cell type: hiPS-derived cells; cell line: C20; differentiation time (days): 2
	hiPSC_C20_4_rep1	cell type: hiPS-derived cells; cell line: C20; differentiation time (days): 4
	hiPSC_C20_4_rep2	cell type: hiPS-derived cells; cell line: C20; differentiation time (days): 4
	hiPSC_C20_30_rep1	cell type: hiPS-derived cells; cell line: C20; differentiation time (days): 30
	hiPSC_C20_30_rep2	cell type: hiPS-derived cells; cell line: C20; differentiation time (days): 30
	hESC_H1_0_rep1	cell type: hESC cell; cell line: H1; differentiation time (days): 0
	hESC_H1_0_rep2	cell type: hESC cell; cell line: H1; differentiation time (days): 0
	hESC_H1_2_rep1	cell type: hESC-derived cells; cell line: H1; differentiation time (days): 2
	hESC_H1_2_rep2	cell type: hESC-derived cells; cell line: H1; differentiation time (days): 2
	hESC_H1_4_rep2	cell type: hESC-derived cells; cell line: H1; differentiation time (days): 4
	hESC_H1_30_rep1	cell type: hESC-derived cells; cell line: H1; differentiation time (days): 30
	hESC_H1_30_rep2	cell type: hESC-derived cells; cell line: H1; differentiation time (days): 30
	hESC_H9_0_rep1	cell type: hESC cell; cell line: H9; differentiation time (days): 0
	hESC_H9_0_rep2	cell type: hESC cell; cell line: H9; differentiation time (days): 0
	hESC_H9_2_rep1	cell type: hESC-derived cells; cell line: H9; differentiation time (days): 2
	hESC_H9_2_rep2	cell type: hESC-derived cells; cell line: H9; differentiation time (days): 2
	hESC_H9_4_rep1	cell type: hESC-derived cells; cell line: H9; differentiation time (days): 4
	hESC_H9_4_rep2	cell type: hESC-derived cells; cell line: H9; differentiation time (days): 4
	hESC_H9_30_rep1	cell type: hESC-derived cells; cell line: H9; differentiation time (days): 30
	hESC_H9_30_rep2	cell type: hESC-derived cells; cell line: H9; differentiation time (days): 30
**GSE47753**	GM12878_50k_rep1	cell line: GM12878; cell type: EBV-transformed; lymphoblastoid cell line; number of cells: 50K
	GM12878_50k_rep2	cell line: GM12878; cell type: EBV-transformed; lymphoblastoid cell line; number of cells: 50K
	GM12878_50k_rep3	cell line: GM12878; cell type: EBV-transformed; lymphoblastoid cell line; number of cells: 50K
	GM12878_50k_rep4	cell line: GM12878; cell type: EBV-transformed; lymphoblastoid cell line; number of cells: 50K

	GM12878_500_rep1	cell line: GM12878; cell type: EBV-transformed; lymphoblastoid cell line; number of cells: 500
	GM12878_500_rep2	cell line: GM12878; cell type: EBV-transformed; lymphoblastoid cell line; number of cells: 500
	GM12878_500_rep3	cell line: GM12878; cell type: EBV-transformed; lymphoblastoid cell line; number of cells: 500
	CD4+_Day1_rep1	cell type: CD4+ T-cells purified using negative selection; day: 1
	CD4+_Day1_rep2	cell type: CD4+ T-cells purified using negative selection; day: 1
	CD4+_Day2_rep1	cell type: CD4+ T-cells purified using negative selection; day: 2
	CD4+_Day2_rep2	cell type: CD4+ T-cells purified using negative selection; day: 2
	CD4+_Day3_rep1	cell type: CD4+ T-cells purified using negative selection; day: 3
	CD4+_Day3_rep2	cell type: CD4+ T-cells purified using negative selection; day: 3
**GSE101571**	2cell_rep1	developmental stage: 2-cell
	2cell_rep2	developmental stage: 2-cell
	2cell_3PN_rep1	developmental stage: 2-cell
	2cell_3PN_rep2	developmental stage: 2-cell
	4cell_3PN_rep1	developmental stage: 4-cell
	4cell_3PN_rep2	developmental stage: 4-cell
	8cell_rep1	developmental stage: 8-cell
	8cell_rep2	developmental stage: 8-cell
	8cell_3PN_rep1	developmental stage: 8-cell
	8cell_3PN_rep2	developmental stage: 8-cell
	ICM_rep1	developmental stage: ICM
	ICM_rep2	developmental stage: ICM
	hESC_rep1	developmental stage: Embryonic stem cell
	hESC_rep2	developmental stage: Embryonic stem cell
**mouse**		
**GSE110261**	CiPS_A_D6_S1	cell type: reprogramming cells; day of reprogramming: day 6; treatment: all chemical
	CiPS_A_D12_S2	cell type: reprogramming cells; day of reprogramming: day 12; treatment: all chemical
	CiPS_A_D18_S3	cell type: reprogramming cells; day of reprogramming: day 18; treatment: all chemical
	CiPS_A_D22_S4	cell type: reprogramming cells; day of reprogramming: day 22; treatment: all chemical
	CiPS_A_D30_S5	cell type: reprogramming cells; day of reprogramming: day 30; treatment: all chemical
	CiPS_A_D36_S6	cell type: reprogramming cells; day of reprogramming: day 36; treatment: all chemical
	CiPS_A_D40_S7	cell type: reprogramming cells; day of reprogramming: day 40; treatment: all chemical
	CiPS_B_D6_S1	cell type: reprogramming cells; day of reprogramming: day 6; treatment: minus Brdu

	CiPS_B_D12_S2	cell type: reprogramming cells; day of reprogramming: day 12; treatment: minus Brdu
	CiPS_B_D18_S3	cell type: reprogramming cells; day of reprogramming: day 18; treatment: minus Brdu
	CiPS_B_D22_S4	cell type: reprogramming cells; day of reprogramming: day 22; treatment: minus Brdu
	CiPS_B_D30_S5	cell type: reprogramming cells; day of reprogramming: day 30; treatment: minus Brdu
	CiPS_B_D36_S6	cell type: reprogramming cells; day of reprogramming: day 36; treatment: minus Brdu
	CiPS_B_D40_S7	cell type: reprogramming cells; day of reprogramming: day 40; treatment: minus Brdu
**GSE79230**	Sperm_rep1	developmental stage: Sperm
	Sperm_rep2	developmental stage: Sperm
	Sperm_rep3	developmental stage: Sperm
**GSE82010**	E0_rep1	strain: C57BL/6; tissue: dentate gyrus; developmental stage: adult 8 wks old
	E0_rep2	strain: C57BL/6; tissue: dentate gyrus; developmental stage: adult 8 wks old
	E0_rep3	strain: C57BL/6; tissue: dentate gyrus; developmental stage: adult 8 wks old
	E0_rep4	strain: C57BL/6; tissue: dentate gyrus; developmental stage: adult 8 wks old
	E1_rep1	strain: C57BL/6; tissue: dentate gyrus; developmental stage: adult 8 wks old
	E1_rep2	strain: C57BL/6; tissue: dentate gyrus; developmental stage: adult 8 wks old
	E1_rep3	strain: C57BL/6; tissue: dentate gyrus; developmental stage: adult 8 wks old
	E1_rep4	strain: C57BL/6; tissue: dentate gyrus; developmental stage: adult 8 wks old
	E4_rep1	strain: C57BL/6; tissue: dentate gyrus; developmental stage: adult 8 wks old
	E4_rep2	strain: C57BL/6; tissue: dentate gyrus; developmental stage: adult 8 wks old
	E4_rep3	strain: C57BL/6; tissue: dentate gyrus; developmental stage: adult 8 wks old
	E4_rep4	strain: C57BL/6; tissue: dentate gyrus; developmental stage: adult 8 wks old
**GSE67298**	MEFs	mouse embryonic fibroblasts
	OSKM-TD sorted day 6	day 6 of OSKM-trans differentiation to Neural Stem Cells
	iPSCs	induced pluripotent stem cells
	ESCs	mouse embryonic stem cells
**fruit fly**		
**GSE104957**	NC14_Anterior_rep1	developmental stage: Embryonic Stage 5
	NC14_Anterior_rep2	developmental stage: Embryonic Stage 5
	NC14_Postrior_rep1	developmental stage: Embryonic Stage 5
	NC14_Postrior_rep2	developmental stage: Embryonic Stage 5
	NC14_Whole_rep1	developmental stage: Embryonic Stage 5
	NC14_Whole_rep2	developmental stage: Embryonic Stage 5
**GSE83851**	NC11_3min_rep1	His2Av-GFP; genotype: Wild type (diploid); timepoint: NC11_03
	NC11_3min_rep2	His2Av-GFP; genotype: Wild type (diploid); timepoint: NC11_03
	NC11_3min_rep3	His2Av-GFP; genotype: Wild type (diploid); timepoint: NC11_03
	NC11_6min_rep1	His2Av-GFP; genotype: Wild type (diploid); timepoint: NC11_06
	NC11_6min_rep2	His2Av-GFP; genotype: Wild type (diploid); timepoint: NC11_06

	NC11_6min_rep3	His2Av-GFP; genotype: Wild type (diploid); timepoint: NC11_06
	NC11_9min_rep1	His2Av-GFP; genotype: Wild type (diploid); timepoint: NC11_09
	NC11_9min_rep2	His2Av-GFP; genotype: Wild type (diploid); timepoint: NC11_09
	NC11_9min_rep3	His2Av-GFP; genotype: Wild type (diploid); timepoint: NC11_09
	NC12_3min_rep1	His2Av-GFP; genotype: Wild type (diploid); timepoint: NC12_03
	NC12_3min_rep2	His2Av-GFP; genotype: Wild type (diploid); timepoint: NC12_03
	NC12_3min_rep3	His2Av-GFP; genotype: Wild type (diploid); timepoint: NC12_03
	NC12_6min_rep1	His2Av-GFP; genotype: Wild type (diploid); timepoint: NC12_06
	NC12_6min_rep2	His2Av-GFP; genotype: Wild type (diploid); timepoint: NC12_06
	NC12_6min_rep3	His2Av-GFP; genotype: Wild type (diploid); timepoint: NC12_06
	NC12_9min_rep1	His2Av-GFP; genotype: Wild type (diploid); timepoint: NC12_09
	NC12_9min_rep2	His2Av-GFP; genotype: Wild type (diploid); timepoint: NC12_09
	NC12_9min_rep3	His2Av-GFP; genotype: Wild type (diploid); timepoint: NC12_09
	NC12_12min_rep1	His2Av-GFP; genotype: Wild type (diploid); timepoint: NC12_12
	NC12_12min_rep2	His2Av-GFP; genotype: Wild type (diploid); timepoint: NC12_12
	NC12_12min_rep3	His2Av-GFP; genotype: Wild type (diploid); timepoint: NC12_12
	NC12_12min_rep4	His2Av-GFP; genotype: Wild type (diploid); timepoint: NC12_12
	NC13_3min_rep1	His2Av-GFP; genotype: Wild type (diploid); timepoint: NC13_03
	NC13_3min_rep2	His2Av-GFP; genotype: Wild type (diploid); timepoint: NC13_03
	NC13_3min_rep3	His2Av-GFP; genotype: Wild type (diploid); timepoint: NC13_03
	NC13_6min_rep1	His2Av-GFP; genotype: Wild type (diploid); timepoint: NC13_06
	NC13_6min_rep2	His2Av-GFP; genotype: Wild type (diploid); timepoint: NC13_06
	NC13_6min_rep3	His2Av-GFP; genotype: Wild type (diploid); timepoint: NC13_06
	NC13_9min_rep1	His2Av-GFP; genotype: Wild type (diploid); timepoint: NC13_09
	NC13_9min_rep2	His2Av-GFP; genotype: Wild type (diploid); timepoint: NC13_09
	NC13_9min_rep3	His2Av-GFP; genotype: Wild type (diploid); timepoint: NC13_09
	NC13_12min_rep1	His2Av-GFP; genotype: Wild type (diploid); timepoint: NC13_12
	NC13_12min_rep2	His2Av-GFP; genotype: Wild type (diploid); timepoint: NC13_12
	NC13_12min_rep3	His2Av-GFP; genotype: Wild type (diploid); timepoint: NC13_12
	NC13_15min_rep1	His2Av-GFP; genotype: Wild type (diploid); timepoint: NC13_15
	NC13_15min_rep2	His2Av-GFP; genotype: Wild type (diploid); timepoint: NC13_15
	NC13_15min_rep3	His2Av-GFP; genotype: Wild type (diploid); timepoint: NC13_15
	NC13_18min_rep1	His2Av-GFP; genotype: Wild type (diploid); timepoint: NC13_18
	NC13_18min_rep2	His2Av-GFP; genotype: Wild type (diploid); timepoint: NC13_18
	NC13_18min_rep3	His2Av-GFP; genotype: Wild type (diploid); timepoint: NC13_18
**yeast**		
**GSE101290**	CEN.PK_WT1_rep1	genotype: CEN.PK; developmental stage: RC start
	CEN.PK_WT1_rep2	genotype: CEN.PK; developmental stage: RC start
	CEN.PK_WT2_rep1	genotype: CEN.PK; developmental stage: RC mid
	CEN.PK_WT2_rep2	genotype: CEN.PK; developmental stage: RC mid
	CEK.PK_WT3_rep1	genotype: CEN.PK; developmental stage: OX mid
	CEK.PK_WT3_rep2	genotype: CEN.PK; developmental stage: OX mid
	CEK.PK_WT4_rep1	genotype: CEN.PK; developmental stage: OX late
	CEK.PK_WT4_rep2	genotype: CEN.PK; developmental stage: OX late

	CEK.PK_WT5_rep1	genotype: CEN.PK; developmental stage: RB start
	CEK.PK_WT5_rep2	genotype: CEN.PK; developmental stage: RB start
	CEK.PK_WT6_rep1	genotype: CEN.PK; developmental stage: RB late
	CEK.PK_WT6_rep2	genotype: CEN.PK; developmental stage: RB late
**GSE66386**	GSY147_lin0_rep1	strain: GSY147; developmental stage: mid-log; population: lin0
	GSY147_lin0_rep2	strain: GSY147; developmental stage: mid-log; population: lin0
	GSY147_lin2_rep1	strain: GSY147; developmental stage: mid-log; population: lin2
	GSY147_lin2_rep2	strain: GSY147; developmental stage: mid-log; population: lin2
	GSY147_lin2_rep3	strain: GSY147; developmental stage: mid-log; population: lin2
	GSY147_lin3_rep1	strain: GSY147; developmental stage: mid-log; population: lin3
	GSY147_lin3_rep2	strain: GSY147; developmental stage: mid-log; population: lin3
	GSY147_lin3_rep3	strain: GSY147; developmental stage: mid-log; population: lin3
	GSY147_lin6_rep1	strain: GSY147; developmental stage: mid-log; population: lin6
	GSY147_lin6_rep2	strain: GSY147; developmental stage: mid-log; population: lin6
	GSY147_lin6_rep3	strain: GSY147; developmental stage: mid-log; population: lin6
**GSE111815**	S288C_wt_rep1	strain: S288C genotype: WT
	S288C_wt_rep2	strain: S288C genotype: WT
	S288C_wt_rep3	strain: S288C genotype: WT
	S288C_Tsh2Δ_rep1	strain: S288C genotype: tSH2delta
	S288C_Tsh2Δ_rep2	strain: S288C genotype: tSH2delta
	S288C_Tsh2Δ_rep3	strain: S288C genotype: tSH2delta
**nematode**		
**GSE114439**	wt_emb_rep1	developmental stage: mixed embryos; strain: wild-type N2
	wt_emb_rep2	developmental stage: mixed embryos; strain: wild-type N2
	wt_L1_rep1	developmental stage: L1 larvae; strain: wild-type N2
	wt_L1_rep2	developmental stage: L1 larvae; strain: wild-type N2
	wt_L2_rep1	developmental stage: L2 larvae; strain: wild-type N2
	wt_L2_rep2	developmental stage: L2 larvae strain: wild-type N2
	wt_L2_rep2	developmental stage: L2 larvae; strain: wild-type N2
	wt_L3_rep1	developmental stage: L3 larvae; strain: wild-type N2
	wt_L3_rep1	developmental stage: L3 larvae; strain: wild-type N2
	wt_L3_rep1	developmental stage: L3 larvae; strain: wild-type N2
	wt_L3_rep2	developmental stage: L3 larvae; strain: wild-type N2
	wt_L3_rep2	developmental stage: L3 larvae; strain: wild-type N2
	wt_L3_rep2	developmental stage: L3 larvae; strain: wild-type N2
	wt_L4_rep1	developmental stage: L4 larvae; strain: wild-type N2
	wt_L4_rep1	developmental stage: L4 larvae; strain: wild-type N2
	wt_L4_rep2	developmental stage: L4 larvae; strain: wild-type N2
	wt_L4_rep2	developmental stage: L4 larvae; strain: wild-type N2
	wt_YA_rep1	developmental stage: young adults; strain: wild-type N2
	wt_YA_rep1	developmental stage: young adults; strain: wild-type N2
	wt_YA_rep1	developmental stage: young adults; strain: wild-type N2
	wt_YA_rep2	developmental stage: young adults; strain: wild-type N2
	glp-1_YA_rep1	developmental stage: day 1 / young adults; strain: glp-1(e2144)

	glp-1_YA_rep1	developmental stage: day 1 / young adults; strain: glp-1(e2144)
	glp-1_YA_rep1	developmental stage: day 1 / young adults; strain: glp-1(e2144)
	glp-1_YA_rep2	developmental stage: day 1 / young adults; strain: glp-1(e2144)
	glp-1_YA_rep2	developmental stage: day 1 / young adults; strain: glp-1(e2144)
**GSE98758**	Rluc rep1	developmental stage: L4; treatment: DNA targeting Renilla luciferase
	Rluc rep2	developmental stage: L4; treatment: DNA targeting Renilla luciferase
	Rluc rep3	developmental stage: L4; treatment: DNA targeting Renilla luciferase
	hmg-3 rep1	developmental stage: L4; knockdown: hmg-3
	hmg-3 rep2	developmental stage: L4; knockdown: hmg-4
	hmg-3 rep3	developmental stage: L4; knockdown: hmg-5
	spt-16 rep1	developmental stage: L4; knockdown: spt-16
	spt-16 rep2	developmental stage: L4; knockdown: spt-17
	spt-16 rep3	developmental stage: L4; knockdown: spt-18
	hmg-4 rep1	developmental stage: L4; knockdown: hmg-4
	hmg-4 rep2	developmental stage: L4; knockdown: hmg-5
	hmg-4 rep3	developmental stage: L4; knockdown: hmg-6

Supplementary Table S2. The mapping information for ATAC-seq samples

human

GEO	Sample	Total Reads	Unique Reads	Unique Rate	Mt. Reads	Mt. Rate
**GSE85332**	hiPSC_C15_0_rep1	99,916,802	21,186,786	21.27%	71,450,478	71.74%
	hiPSC_C15_0_rep2	70,373,802	15,686,915	22.36%	50,147,729	71.49%
	hiPSC_C15_2_rep1	82,567,698	41,705,425	50.78%	34,267,800	41.73%
	hiPSC_C15_2_rep2	93,595,606	39,964,973	42.88%	45,788,710	49.13%
	hiPSC_C15_4_rep1	106,478,372	57,293,517	54.18%	37,512,344	35.48%
	hiPSC_C15_4_rep2	89,956,112	37,622,522	42.09%	43,043,815	48.16%
	hiPSC_C15_30_rep1	99,914,896	16,097,846	16.18%	74,440,021	74.84%
	hiPSC_C15_30_rep2	77,472,370	14,620,522	18.94%	55,854,516	72.36%
	hiPSC_C20_0_rep1	64,966,186	20,216,687	31.22%	39,523,395	61.04%
	hiPSC_C20_0_rep2	90,087,266	34,630,928	38.59%	48,316,836	53.83%
	hiPSC_C20_2_rep1	107,859,924	34,708,127	32.36%	63,003,176	58.74%
	hiPSC_C20_2_rep2	75,205,954	22,230,337	29.68%	46,595,118	62.21%
	hiPSC_C20_4_rep1	98,471,190	34,334,684	35.05%	56,469,564	57.64%
	hiPSC_C20_4_rep2	179,332,572	55,500,185	31.10%	109,518,485	61.36%
	hiPSC_C20_30_rep1	99,405,994	27,310,526	27.58%	63,357,080	63.98%
	hiPSC_C20_30_rep2	72,770,936	20,465,357	28.26%	45,434,178	62.74%
	hESC_H1_0_rep1	71,721,874	21,188,734	29.64%	45,537,744	63.71%
	hESC_H1_0_rep2	87,538,268	26,193,399	30.01%	54,691,099	62.65%
	hESC_H1_2_rep1	62,913,270	19,671,039	31.42%	39,229,257	62.67%
	hESC_H1_2_rep2	66,053,200	18,535,342	28.19%	43,597,767	66.31%
	hESC_H1_4_rep1	88,524,290	37,830,813	42.94%	43,318,441	49.17%
	hESC_H1_4_rep2	125,134,604	41,045,413	32.98%	72,768,862	58.47%

	hESC_H1_30_rep1	75,506,006	41,851,893	55.95%	24,217,279	32.37%
	hESC_H1_30_rep2	36,022,224	18,527,317	51.90%	13,201,907	36.98%
	hESC_H9_0_rep1	105,201,672	33,676,909	32.13%	62,700,892	59.82%
	hESC_H9_0_rep2	71,603,318	21,801,146	30.56%	44,298,009	62.09%
	hESC_H9_2_rep1	61,135,780	14,525,326	23.88%	41,649,629	68.47%
	hESC_H9_2_rep2	75,029,914	20,610,914	27.60%	47,536,100	63.66%
	hESC_H9_4_rep1	123,241,202	44,165,947	36.13%	66,841,108	54.67%
	hESC_H9_4_rep2	93,650,702	26,210,751	28.11%	59,835,041	64.17%
	hESC_H9_30_rep1	75,601,476	21,196,150	28.13%	47,599,057	63.17%
	hESC_H9_30_rep2	72,742,916	24,459,832	33.77%	41,312,023	57.04%
**GSE47753**	GM12878_50k_rep1	381,346,046	209,109,164	54.83%	98,349,374	47.03%
	GM12878_50k_rep2	111,511,322	60,948,652	54.66%	32,907,372	53.99%
	GM12878_50k_rep3	167,991,688	82,394,422	49.05%	58,084,948	70.50%
	GM12878_50k_rep4	123,665,388	61,857,296	50.02%	42,971,270	69.47%
	GM12878_500_rep1	45,716,944	32,427,682	70.93%	5,917,278	18.25%
	GM12878_500_rep2	45,015,880	30,440,524	67.62%	8,697,122	28.57%
	GM12878_500_rep3	46,686,754	26,077,672	55.86%	15,441,184	59.21%
	CD4+_Day1_rep1	16,016,616	8,135,438	50.79%	4,266,936	52.45%
	CD4+_Day1_rep2	22,290,612	11,332,734	50.84%	6,002,548	52.97%
	CD4+_Day2_rep1	21,453,348	11,396,036	53.12%	2,200,726	19.31%
	CD4+_Day2_rep2	18,085,584	9,958,718	55.06%	4,284,656	43.02%
	CD4+_Day3_rep1	51,939,082	29,988,188	57.74%	994,992	3.32%
	CD4+_Day3_rep2	59,288,748	33,292,358	56.15%	3,740,148	11.23%
**GSE101571**	2cell_rep1	111,683,240	8,111,527	8.06%	64,016,994	63.58%
	2cell_rep2	419,555,092	5,547,687	1.42%	328,775,678	83.92%
	2cell_3PN_rep1	179,101,680	8,100,193	4.71%	130,070,672	75.64%
	2cell_3PN_rep2	434,441,264	17,109,328	4.19%	294,970,708	72.16%
	4cell_3PN_rep1	94,017,604	10,115,122	11.46%	49,419,716	55.98%
	4cell_3PN_rep2	208,528,358	9,218,333	4.88%	130,025,002	68.86%
	8cell_rep1	130,574,124	9,995,209	8.41%	62,862,312	52.92%
	8cell_rep2	141,696,270	7,348,360	5.84%	73,600,170	58.47%
	8cell_3PN_rep1	398,852,222	31,795,431	8.96%	236,462,698	66.64%
	8cell_3PN_rep2	665,125,864	54,816,373	8.72%	469,740,058	74.76%
	ICM_rep1	507,241,948	52,568,241	19.42%	133,952,354	49.47%
	ICM_rep2	414,158,866	37,632,517	17.05%	103,978,268	47.10%
	hESC_rep1	37,066,914	5,814,990	18.03%	15,004,184	46.51%
	hESC_rep2	83,764,902	12,422,873	15.64%	36,899,772	46.46%
**mouse**						
**GSE110261**	CiPS_A_D6_S1	26,157,844	11,440,358	44.92%	8,797,111	34.54%
	CiPS_A_D12_S2	34,725,592	17,529,331	51.72%	9,465,325	27.92%
	CiPS_A_D18_S3	14,895,548	7,595,632	52.15%	4,159,674	28.56%
	CiPS_A_D22_S4	22,473,408	9,648,198	43.85%	7,915,581	35.98%
	CiPS_A_D30_S5	18,696,576	9,527,037	52.12%	5,052,448	27.64%
	CiPS_A_D36_S6	18,399,102	6,293,603	34.93%	8,181,454	45.41%

	CiPS_A_D40_S7	17,388,774	7,059,223	41.36%	6,754,559	39.58%
	CiPS_B_D6_S1	39,411,600	16,539,432	43.23%	13,833,292	36.16%
	CiPS_B_D12_S2	61,055,894	30,612,963	51.54%	16,610,353	27.96%
	CiPS_B_D18_S3	37,613,222	22,446,292	61.00%	7,421,807	20.17%
	CiPS_B_D22_S4	40,416,090	19,215,813	48.43%	13,194,514	33.26%
	CiPS_B_D30_S5	24,952,536	13,701,285	56.33%	4,569,624	18.79%
	CiPS_B_D36_S6	23,745,394	14,902,132	64.62%	2,665,552	11.56%
	CiPS_B_D40_S7	28,085,848	18,315,348	67.25%	2,436,139	8.95%
**GSE79230**	Sperm_rep1	118,936,748	84,484,278	71.03%	2,325,922	2.75%
	Sperm_rep2	305,894,370	218,429,660	71.41%	11,735,552	5.37%
	Sperm_rep3	375,906,738	249,640,094	66.41%	6,311,194	2.53%
**GSE82010**	E0_rep1	14,326,266	9,770,906	68.20%	739,662	7.57%
	E0_rep2	19,247,784	13,229,250	68.73%	809,020	6.12%
	E0_rep3	30,865,616	14,195,274	45.99%	9,806,820	69.09%
	E0_rep4	42,869,262	19,626,574	45.78%	13,799,972	70.31%
	E1_rep1	19,860,444	12,731,560	64.11%	1,331,878	10.46%
	E1_rep2	18,001,874	12,003,958	66.68%	857,894	7.15%
	E1_rep3	24,322,776	11,885,320	48.86%	7,996,742	67.28%
	E1_rep4	31,416,342	14,683,418	46.74%	11,067,716	75.38%
	E4_rep1	37,023,628	25,287,130	68.30%	1,313,346	5.19%
	E4_rep2	8,282,672	5,107,056	61.66%	410,506	8.04%
	E4_rep3	15,302,474	7,507,344	49.06%	4,101,608	54.63%
	E4_rep4	17,317,136	8,095,590	46.75%	5,401,060	66.72%
**GSE67298**	MEFs	115,655,582	63,967,813	58.71%	27,406,592	25.15%
	OSKM-TD sorted day 6	116,786,562	60,902,759	53.33%	36,313,924	31.80%
	iPSCs	129,307,976	45,087,349	35.27%	60,119,449	47.03%
	ESCs	102,863,886	42,907,174	42.14%	42,298,673	41.54%
**fruit fly**						
**GSE104957**	NC14_Anterior_rep1	73,850,572	20,175,354	29.72%	39,481,588	58.17%
	NC14_Anterior_rep2	100,052,084	51,736,585	55.06%	23,865,256	25.40%
	NC14_Postrior_rep1	38,698,110	11,389,565	30.67%	21,144,289	56.94%
	NC14_Postrior_rep2	68,817,046	10,773,782	52.39%	5,575,472	27.11%
	NC14_Whole_rep1	94,324,024	32,942,911	36.04%	47,345,376	51.80%
	NC14_Whole_rep2	87,256,114	40,233,835	48.09%	31,361,641	37.49%
**GSE83851**	NC11_3min_rep1	32,363,834	3,732,440	11.67%	26,555,264	83.02%
	NC11_3min_rep2	36,331,092	4,701,894	13.13%	29,117,951	81.29%
	NC11_3min_rep3	47,642,060	4,954,439	10.50%	40,054,900	84.86%
	NC11_6min_rep1	26,118,294	4,721,453	18.30%	19,104,677	74.06%
	NC11_6min_rep2	26,340,594	4,839,380	18.57%	18,903,887	72.55%
	NC11_6min_rep3	29,850,260	6,665,191	22.69%	19,625,727	66.81%
	NC11_9min_rep1	38,071,344	5,402,747	14.38%	29,922,890	79.63%
	NC11_9min_rep2	30,297,624	4,459,380	14.91%	23,544,485	78.70%
	NC11_9min_rep3	51,272,612	8,795,747	17.35%	38,226,307	75.41%

	NC12_3min_rep1	47,392,128	12,304,251	26.37%	28,455,936	60.98%
	NC12_3min_rep2	42,285,844	10,284,337	24.70%	27,025,795	64.91%
	NC12_3min_rep3	31,704,640	8,010,173	25.61%	19,550,243	62.51%
	NC12_6min_rep1	43,017,826	11,109,260	26.23%	25,766,963	60.84%
	NC12_6min_rep2	46,199,026	15,429,200	33.86%	24,023,911	52.72%
	NC12_6min_rep3	36,404,360	10,086,213	28.10%	20,914,338	58.26%
	NC12_9min_rep1	42,381,654	14,538,451	34.74%	21,556,880	51.51%
	NC12_9min_rep2	37,615,846	10,523,949	28.36%	21,675,849	58.42%
	NC12_9min_rep3	38,561,258	9,344,744	24.48%	25,662,138	67.23%
	NC12_12min_rep1	41,435,358	13,356,975	32.91%	21,230,850	52.32%
	NC12_12min_rep2	36,305,922	10,466,837	31.62%	18,606,291	56.21%
	NC12_12min_rep3	39,126,302	9,865,848	25.56%	24,463,464	63.38%
	NC12_12min_rep4	50,508,290	11,139,172	22.32%	34,042,982	68.21%
	NC13_3min_rep1	45,734,214	13,929,083	30.88%	25,064,554	55.56%
	NC13_3min_rep2	36,875,106	10,851,531	29.82%	20,688,120	56.85%
	NC13_3min_rep3	27,863,110	10,692,115	38.98%	12,026,590	43.84%
	NC13_6min_rep1	43,163,640	19,802,548	46.53%	15,389,550	36.16%
	NC13_6min_rep2	30,380,970	14,848,046	49.75%	8,721,817	29.23%
	NC13_6min_rep3	48,321,822	19,287,629	40.52%	20,480,553	43.03%
	NC13_9min_rep1	46,612,874	19,670,195	42.83%	18,759,865	40.85%
	NC13_9min_rep2	29,070,086	12,346,035	43.38%	11,335,075	39.82%
	NC13_9min_rep3	36,908,342	15,967,715	43.91%	14,788,243	40.66%
	NC13_12min_rep1	30,445,290	12,949,962	43.20%	12,020,873	40.10%
	NC13_12min_rep2	32,137,024	13,425,177	42.36%	13,472,642	42.51%
	NC13_12min_rep3	39,094,394	18,916,880	49.21%	11,688,621	30.41%
	NC13_15min_rep1	35,565,706	15,516,015	44.25%	14,769,861	42.12%
	NC13_15min_rep2	27,678,174	12,004,916	44.09%	10,410,834	38.24%
	NC13_15min_rep3	25,805,092	12,515,150	49.22%	8,366,290	32.91%
	NC13_18min_rep1	49,936,014	17,783,730	36.14%	24,654,826	50.11%
	NC13_18min_rep2	31,657,548	17,930,769	57.56%	6,484,910	20.82%
	NC13_18min_rep3	27,438,036	12,469,752	46.10%	9,782,906	36.16%
**yeast**						
**GSE101290**	CEN.PK_WT1_rep1	12,539,154	6,857,232	58.34%	291,257	2.48%
	CEN.PK_WT1_rep2	13,724,364	7,487,593	57.81%	240,802	1.86%
	CEN.PK_WT2_rep1	12,556,386	7,055,542	60.02%	153,880	1.31%
	CEN.PK_WT2_rep2	17,246,168	10,364,693	63.65%	260,551	1.60%
	CEK.PK_WT3_rep1	10,371,790	6,061,063	62.21%	138,006	1.42%
	CEK.PK_WT3_rep2	11,157,896	6,357,019	61.13%	139,887	1.35%
	CEK.PK_WT4_rep1	10,357,488	5,613,733	57.43%	246,613	2.52%
	CEK.PK_WT4_rep2	10,536,432	5,492,220	55.53%	214,632	2.17%
	CEK.PK_WT5_rep1	11,263,756	6,062,441	57.20%	398,867	3.76%
	CEK.PK_WT5_rep2	9,929,528	5,233,447	56.52%	411,708	4.45%
	CEK.PK_WT6_rep1	13,489,170	7,276,779	57.67%	401,254	3.18%
	CEK.PK_WT6_rep2	12,678,328	7,108,999	59.80%	327,250	2.75%

**GSE66386**	GSY147_lin0_rep1	21,923,082	11,883,552	54.21%	612,636	5.16%
	GSY147_lin0_rep2	34,073,336	18,773,138	55.10%	577,336	3.08%
	GSY147_lin2_rep1	17,696,926	9,385,724	53.04%	397,530	4.24%
	GSY147_lin2_rep2	38,094,746	20,746,876	54.46%	687,830	3.32%
	GSY147_lin2_rep3	25,779,538	13,317,038	51.66%	573,302	4.31%
	GSY147_lin3_rep1	11,528,808	5,579,054	48.39%	201,140	3.61%
	GSY147_lin3_rep2	26,542,054	12,867,398	48.48%	508,088	3.95%
	GSY147_lin3_rep3	18,832,474	9,080,664	48.22%	397,666	4.38%
	GSY147_lin6_rep1	13,521,810	6,792,240	50.23%	370,582	5.46%
	GSY147_lin6_rep2	38,538,338	19,632,436	50.94%	643,360	3.28%
	GSY147_lin6_rep3	52,181,678	27,586,904	52.87%	2,608,042	9.45%
**GSE111815**	S288C_wt_rep1	63,823,882	37,687,734	59.05%	16,860,612	44.74%
	S288C_wt_rep2	56,667,058	30,645,650	54.08%	13,001,888	42.43%
	S288C_wt_rep3	80,381,080	44,857,980	55.81%	17,974,764	40.07%
	S288C_Tsh2Δ_rep1	47,255,876	23,722,680	50.20%	8,988,880	37.89%
	S288C_Tsh2Δ_rep2	64,457,116	34,721,998	53.87%	17,142,846	49.37%
	S288C_Tsh2Δ_rep3	51,920,356	25,896,120	49.88%	8,807,280	34.01%
**nematode**						
**GSE114439**	wt_emb_ATAC-seq_rep1	115,245,456	100,874,094	87.53%	32,407,830	32.13%
	wt_emb_ATAC-seq_rep2	87,726,090	78,098,764	89.03%	29,415,062	37.66%
	wt_L1_ATAC-seq_rep1	64,099,078	49,327,652	76.96%	8,775,516	17.79%
	wt_L1_ATAC-seq_rep2	73,860,568	59,430,666	80.46%	16,084,314	27.06%
	wt_L2_ATAC-seq_rep1	58,368,970	52,032,714	89.14%	12,421,406	23.87%
	wt_L2_ATAC-seq_rep2	46,100,430	42,046,482	91.21%	12,842,132	30.54%
	wt_L2_ATAC-seq_rep2	91,578,486	83,194,962	90.85%	25,933,374	31.17%
	wt_L3_ATAC-seq_rep1	20,732,048	16,057,288	77.45%	5,393,556	33.59%
	wt_L3_ATAC-seq_rep1	13,831,394	11,748,232	84.94%	3,979,038	33.87%
	wt_L3_ATAC-seq_rep1	57,857,658	49,772,952	86.03%	16,361,720	32.87%
	wt_L3_ATAC-seq_rep2	42,129,426	33,737,744	80.08%	13,079,876	38.77%
	wt_L3_ATAC-seq_rep2	16,439,308	14,284,908	86.89%	5,580,494	39.07%
	wt_L3_ATAC-seq_rep2	66,200,696	58,166,872	87.86%	22,091,916	37.98%
	wt_L4_ATAC-seq_rep1	57,795,816	47,138,212	81.56%	16,269,714	34.51%
	wt_L4_ATAC-seq_rep1	75,146,682	66,731,322	88.80%	22,698,660	34.02%
	wt_L4_ATAC-seq_rep2	70,529,738	62,759,938	88.98%	26,992,936	43.01%
	wt_L4_ATAC-seq_rep2	77,845,006	69,881,940	89.77%	29,404,298	42.08%
	wt_YA_ATAC-seq_rep1	68,230,284	58,245,014	85.37%	35,035,806	60.15%
	wt_YA_ATAC-seq_rep1	54,535,732	50,017,968	91.72%	38,777,806	77.53%
	wt_YA_ATAC-seq_rep1	76,800,972	70,232,100	91.45%	54,761,738	77.97%
	wt_YA_ATAC-seq_rep2	156,412,458	131,702,822	84.20%	50,156,000	38.08%
	glp-1_YA_ATAC-seq_rep1	28,763,789	21,978,428	76.41%	1,189,960	5.41%
	glp-1_YA_ATAC-seq_rep1	4,907,245	3,708,904	75.58%	200,470	5.41%
	glp-1_YA_ATAC-seq_rep1	2,821,831	2,161,056	76.58%	116,914	5.41%
	glp-1_YA_ATAC-seq_rep2	12,819,956	9,459,017	73.78%	434,061	4.59%
	glp-1_YA_ATAC-seq_rep2	42,522,055	31,570,284	74.24%	1,410,453	4.47%

**GSE98758**	Rluc rep1	122,225,996	82,758,985	75.91%	8,867,339	8.13%
	Rluc rep2	109,859,054	74,784,253	75.54%	8,584,860	8.67%
	Rluc rep3	86,797,024	57,578,793	73.70%	8,515,740	10.90%
	hmg-3 rep1	54,048,084	27,038,510	66.37%	8,057,888	19.78%
	hmg-3 rep2	133,922,524	62,498,591	65.09%	20,064,439	20.90%
	hmg-3 rep3	131,108,090	66,808,153	72.64%	8,820,596	9.59%
	spt-16 rep1	99,103,686	65,983,369	74.67%	8,692,909	9.84%
	spt-16 rep2	100,925,820	63,481,144	71.96%	12,096,774	13.71%
	spt-16 rep3	49,360,446	30,641,021	68.69%	7,052,195	15.81%
	hmg-4 rep1	129,248,160	91,621,321	77.26%	8,374,530	7.06%
	hmg-4 rep2	106,042,726	73,683,026	75.21%	8,457,616	8.63%
	hmg-4 rep3	82,634,064	57,145,020	74.98%	7,022,858	9.21%

Arabidopsis thaliana

**GEO**	**Sample**	**Total Reads**	**Unique Reads**	**Unique Rate**	**Mt. Reads**	**Mt. Rate**	**Cp. Reads**	**Cp. Rate**
**GSE101940**	stem.cell_rep1	80,854,120	54,578,228	67.50%	1,454,444	2.66%	22,007,036	40.32%
	stem.cell_rep2	95,127,376	67,722,354	71.19%	1,562,544	2.31%	23,077,104	34.08%
	stem.cell_rep3	144,826,386	95,201,324	65.73%	2,834,132	2.98%	41,359,516	43.44%
	mesophyll.cell_rep1	107,997,822	57,352,908	53.11%	1,779,038	3.10%	41,525,500	72.40%
	mesophyll.cell_rep2	95,847,940	54,890,028	57.27%	1,776,566	3.24%	32,243,990	58.74%
	mesophyll.cell_rep3	95,895,784	52,410,942	54.65%	1,719,934	3.28%	36,339,100	69.33%
**GSE89346**	14-day_Col-0_seedling	60356262	26135178	43.30%	854,700	3.27%	19,039,596	72.85%
**GSE101482**	root.tip_rep1	43,012,610	33,453,200	77.78%	769,650	2.30%	1,606,962	4.80%
	root.tip_rep2	107,155,128	82,412,726	76.91%	1,282,202	1.56%	2,462,578	2.99%
	root.tip_Crude_rep1	128,458,992	68,775,522	53.54%	14,491,578	21.07%	18,237,524	26.52%
	root.tip_Crude_rep2	116,596,664	61,634,658	52.86%	13,592,458	22.05%	16,231,800	26.34%
	root.non.hair_rep1	89,601,162	75,359,116	84.11%	2,174,198	2.89%	2,087,822	2.77%
	root.non.hair_rep2	22,651,690	17,494,116	77.23%	711,368	4.07%	1,090,864	6.24%
	root.hair_rep1	102,248,110	83,066,444	81.24%	2,295,864	2.76%	3,282,000	3.95%
	root.hair_rep2	27,413,70	21,774,51	79.43	791,710	3.64%	1,673,71	7.69%

	8	8	%			0	
genomic	389,748,3	274,279,7	70.37	3,272,30	1.19%	45,883,0	16.73%
	10	56	%	4		54	

Supplementary Table S3. Transcription factors identified from all samples.

yeast	fruit fly	nematode	*A. thaliana*	human	mouse
YBR049C	FBgn0259789	WBGene00004013	AT3G27010	ENSG00000185591	ENSMUSG00000001280
YBR049C	FBgn0000286	WBGene00023497	AT2G36270	ENSG00000001167	ENSMUSG00000075304
YKL038W	FBgn0000120	WBGene00001161	AT5G53210	ENSG00000204335	ENSMUSG00000029178
YDR303C	FBgn0085432	WBGene00001061	AT1G49720	ENSG00000109787	ENSMUSG00000018678
YNL216W	FBgn0000625	WBGene00001948	AT1G32150	ENSG00000167182	ENSMUSG00000023994
YHL020C	FBgn0003448	WBGene00003024	AT2G18160	ENSG00000158711	ENSMUSG00000005148
YGL162W	FBgn0011278	WBGene00001174	AT1G69010	ENSG00000120690	ENSMUSG00000000078
YLR182W	FBgn0003507	WBGene00004096	AT3G10800	ENSG00000126767	ENSMUSG00000033863
YKL062W	FBgn0004053	WBGene00001251	AT1G45249	ENSG00000102554	ENSMUSG00000073209
YLR403W	FBgn0003117	WBGene00001324	AT3G56850	ENSG00000067082	ENSMUSG00000058440
YHR006W	FBgn0004198	WBGene00003847	AT2G35530	ENSG00000119138	ENSMUSG00000003032
YKL185W	FBgn0004595	WBGene00006873	AT2G04038	ENSG00000151702	ENSMUSG00000026436
YGL237C	FBgn0003870	WBGene00004804	AT4G30410	ENSG00000175832	ENSMUSG00000009406
YBL054W	FBgn0004837	WBGene00003180	AT3G62420	ENSG00000154727	ENSMUSG00000036442
YLL054C	FBgn0037976	WBGene00001086	AT4G34590	ENSG00000266265	ENSMUSG00000036461
YOR344C	FBgn0000576	WBGene00006818	AT3G23210	ENSG00000006468	ENSMUSG00000037465
YPL075W	FBgn0000567	WBGene00001434	AT5G15830	ENSG00000102034	ENSMUSG00000095276
YOR380W	FBgn0000015	WBGene00000908	AT4G36780	ENSG00000168286	ENSMUSG00000016087
YOR358W	FBgn0001235	WBGene00020930	AT2G43010	ENSG00000106459	ENSMUSG00000008976
YGL254W	FBgn0003720	WBGene00002987	AT2G46270	ENSG00000134954	ENSMUSG00000030678
YDL106C	FBgn0004050	WBGene00001949	AT1G78700	ENSG00000136826	ENSMUSG00000017724
YPR199C	FBgn0011723	WBGene00000435	AT3G59060	ENSG00000261949	ENSMUSG00000004151
YDR253C	FBgn0013799	WBGene00001950	AT4G24470	ENSG00000157554	ENSMUSG00000031103
YOL028C	FBgn0002985	WBGene00006654	AT4G15090	ENSG00000105672	ENSMUSG00000023027
YDL020C	FBgn0013263	WBGene00003003	AT3G21175	ENSG00000102974	ENSMUSG00000032035
YNL139C	FBgn0031232	WBGene00004858	AT5G61270	ENSG00000100811	ENSMUSG00000000134
YJR060W	FBgn0000166	WBGene00001568	AT5G45300	ENSG00000135373	ENSMUSG00000058239
YFR034C	FBgn0000251	WBGene00003912	AT4G18890	ENSG00000155090	ENSMUSG00000099083
YOR113W	FBgn0037621	WBGene00022518	AT5G01380	ENSG00000135374	ENSMUSG00000006311
YDL056W	FBgn0015664	WBGene00015934	AT4G38170	ENSG00000103495	ENSMUSG00000027104
YPL038W	FBgn0033748	WBGene00000483	AT1G10120	ENSG00000118260	ENSMUSG00000006705
YER169W	FBgn0283451	WBGene00000445	AT5G11260	ENSG00000205250	ENSMUSG00000014859
YBR089C	FBgn0263864	WBGene00006796	AT5G59990	ENSG00000124664	ENSMUSG00000035158
YBL005W	FBgn0004110	WBGene00000469	AT5G23280	ENSG00000124092	ENSMUSG00000030256
YPR052C	FBgn0026309	WBGene00001953	AT3G22170	ENSG00000118513	ENSMUSG00000026641
YEL009C	FBgn0010323	WBGene00001954	AT5G02320	ENSG00000068323	ENSMUSG00000040732
YDR043C	FBgn0000504	WBGene00001951	AT2G01930	ENSG00000087903	ENSMUSG00000054191

YPL133C	FBgn0002723	WBGene00000468	AT3G45150	ENSG00000078399	ENSMUSG00000038227
YER068W	FBgn0003028		AT5G07100	ENSG00000130522	ENSMUSG00000038718
YDR207C	FBgn0000492		AT4G16150	ENSG00000163435	ENSMUSG00000056749
YPR104C	FBgn0004860		AT5G38860	ENSG00000185697	ENSMUSG00000071076
YGL209W	FBgn0284220		AT5G08130	ENSG00000165030	ENSMUSG00000030103
YPL139C	FBgn0003900		AT5G42520	ENSG00000143390	ENSMUSG00000012350
YER148W	FBgn0004870		AT5G67300	ENSG00000101057	ENSMUSG00000027186
YHR206W	FBgn0003944		AT1G68640	ENSG00000167081	ENSMUSG00000029238
YKL043W	FBgn0027364		AT1G01720	ENSG00000160199	ENSMUSG00000019230
YKL015W	FBgn0039915		AT5G65310	ENSG00000112242	ENSMUSG00000021264
YLR013W	FBgn0001325		AT5G08330	ENSG00000123268	ENSMUSG00000048047
YKL222C	FBgn0086680		AT5G06839	ENSG00000115966	ENSMUSG00000040929
YNL309W	FBgn0020379		AT3G12250	ENSG00000111704	ENSMUSG00000015522
YNL314W	FBgn0004636		AT5G09410	ENSG00000177485	ENSMUSG00000016477
YDR259C	FBgn0004862		AT2G47070	ENSG00000143355	ENSMUSG00000026815
YJR094C	FBgn0005386		AT4G27900	ENSG00000168610	ENSMUSG00000024206
YKR034W	FBgn0033749		AT2G30590	ENSG00000122877	ENSMUSG00000004040
YMR037C	FBgn0004914		AT1G29280	ENSG00000158773	ENSMUSG00000037868
YGL166W	FBgn0002521		AT1G08320	ENSG00000134852	ENSMUSG00000024215
YKL109W	FBgn0000233		AT5G46350	ENSG00000166949	ENSMUSG00000052534
YML081W	FBgn0030408		AT1G69690	ENSG00000269404	ENSMUSG00000005774
YER040W	FBgn0035625		AT3G61250	ENSG00000185630	ENSMUSG00000034673
YOR028C	FBgn0003460		AT4G01250	ENSG00000132005	ENSMUSG00000012396
YGL096W	FBgn0260632		AT4G05100	ENSG00000187098	ENSMUSG00000022346
YNL199C	FBgn0259211		AT1G52880	ENSG00000066336	ENSMUSG00000019982
YPL128C	FBgn0003002		AT5G52830	ENSG00000134107	ENSMUSG00000020185
YFL031W	FBgn0011648		AT2G17950	ENSG00000170653	ENSMUSG00000037169
YGR249W	FBgn0003169		AT5G02030	ENSG00000107187	ENSMUSG00000059436
YHL027W	FBgn0054027		AT1G75080	ENSG00000123095	ENSMUSG00000026934
YMR016C	FBgn0260642		AT5G06950	ENSG00000169016	ENSMUSG00000038418
YIL131C	FBgn0005694		AT1G53230	ENSG00000204644	ENSMUSG00000057469
YLR266C	FBgn0261930		AT3G15270	ENSG00000105698	ENSMUSG00000041515
YHR084W	FBgn0001320		AT2G24570	ENSG00000016082	ENSMUSG00000003051
YER088C	FBgn0011655		AT3G57920	ENSG00000101412	ENSMUSG00000017861
YGL131C	FBgn0003145		AT2G42200	ENSG00000140968	ENSMUSG00000070495
YBR089C-A	FBgn0020912		AT1G30650	ENSG00000143437	ENSMUSG00000021109
YPL202C	FBgn0034970		AT2G46130	ENSG00000136997	ENSMUSG00000008193
YDR123C	FBgn0000659		AT4G26640	ENSG00000105610	ENSMUSG00000025912
YFL021W	FBgn0001222		AT5G03150	ENSG00000165891	ENSMUSG00000032015
YDR216W	FBgn0011758		AT2G38470	ENSG00000120738	ENSMUSG00000005698
YDR169C	FBgn0261434		AT3G13810	ENSG00000204304	ENSMUSG00000062939
YPR065W	FBgn0014143		AT5G03790	ENSG00000128710	ENSMUSG00000031706
YHR056C	FBgn0000527		AT1G72010	ENSG00000253293	ENSMUSG00000008496
YOL089C	FBgn0024250		AT2G22430	ENSG00000198914	ENSMUSG00000027490

YGL035C	FBgn0085424	AT5G65210	ENSG00000125952	ENSMUSG00000026104
YMR021C	FBgn0019650	AT2G46680	ENSG00000134323	ENSMUSG00000000282
YCR097W	FBgn0045759	AT1G23380	ENSG00000028277	ENSMUSG00000055116
YKR099W	FBgn0038805	AT1G80840	ENSG00000137709	ENSMUSG00000045515
YGL073W	FBgn0015602	AT4G31800	ENSG00000204531	ENSMUSG00000071637
YJL110C	FBgn0001291	AT5G44160	ENSG00000100644	ENSMUSG00000002111
YDR477W	FBgn0001168	AT5G62470	ENSG00000138378	ENSMUSG00000004043
YBL103C	FBgn0040465	AT1G55110	ENSG00000115415	ENSMUSG00000001988
YML007W	FBgn0014343	AT2G23320	ENSG00000126561	ENSMUSG00000032402
YLR451W	FBgn0267337	AT4G36740	ENSG00000185002	ENSMUSG00000024406
YLR176C	FBgn0003065	AT2G30250	ENSG00000185668	ENSMUSG00000022463
YLR098C	FBgn0019661	AT3G23250	ENSG00000143995	ENSMUSG00000090125
YPL049C		AT5G46830	ENSG00000184937	ENSMUSG00000036036
YLR375W		AT1G69490	ENSG00000070444	ENSMUSG00000014030
YPR070W		AT5G41570	ENSG00000130751	ENSMUSG00000003949
YDL048C		AT3G01970	ENSG00000159216	ENSMUSG00000024140
YMR042W		AT1G29860	ENSG00000162702	ENSMUSG00000068551
YBR083W		AT1G24260	ENSG00000005073	ENSMUSG00000042258
YDL170W		AT5G04390	ENSG00000260596	ENSMUSG00000033837
YCR106W		AT3G50700	ENSG00000175387	ENSMUSG00000042406
YOR337W		AT1G62300	ENSG00000168214	ENSMUSG00000058794
YMR019W		AT2G03340	ENSG00000160973	ENSMUSG00000016458
YCR018C		AT4G23550	ENSG00000164916	ENSMUSG00000015839
YGL071W		AT4G04450	ENSG00000133794	ENSMUSG00000040270
YDR213W		AT4G22070	ENSG00000163623	ENSMUSG00000001819
YDR423C		AT3G04070	ENSG00000182759	ENSMUSG00000029135
YPL021W		AT1G03840	ENSG00000196092	ENSMUSG00000025529
YGL181W		AT4G24240	ENSG00000177426	ENSMUSG00000039191
		AT1G09540	ENSG00000128272	ENSMUSG00000095139
		AT2G40740	ENSG00000115112	ENSMUSG00000000247
		AT4G18170	ENSG00000128713	ENSMUSG00000056216
		AT5G66700	ENSG00000108924	ENSMUSG00000013089
		AT2G46400	ENSG00000112182	ENSMUSG00000028890
		AT3G51470	ENSG00000181444	ENSMUSG00000030796
		AT1G64625	ENSG00000116016	ENSMUSG00000058669
		AT1G20980	ENSG00000114861	ENSMUSG00000003184
		AT5G26170	ENSG00000141646	ENSMUSG00000028901
		AT5G13080	ENSG00000168310	ENSMUSG00000048387
		AT4G31550	ENSG00000123358	ENSMUSG00000005836
		AT1G77920	ENSG00000150907	ENSMUSG00000042589
		AT3G58780	ENSG00000153779	ENSMUSG00000038346
		AT1G75390	ENSG00000119547	ENSMUSG00000024912
		AT4G01720	ENSG00000147180	ENSMUSG00000026628
		AT1G69780	ENSG00000203989	ENSMUSG00000052837

AT1G76110	ENSG00000162419	ENSMUSG00000034266
AT3G42860	ENSG00000124813	ENSMUSG00000052684
AT5G50570	ENSG00000214717	ENSMUSG00000025612
AT4G38620	ENSG00000172273	ENSMUSG00000018143
AT5G22330	ENSG00000007968	ENSMUSG00000022952
AT5G56110	ENSG00000184302	ENSMUSG00000039153
AT3G02940	ENSG00000007866	ENSMUSG00000047591
AT1G49480	ENSG00000159184	ENSMUSG00000025408
AT3G01530	ENSG00000119725	ENSMUSG00000074622
AT1G18570	ENSG00000165588	ENSMUSG00000036139
AT1G20910	ENSG00000165462	ENSMUSG00000055320
AT5G66730	ENSG00000175325	ENSMUSG00000030353
AT4G21030	ENSG00000273706	ENSMUSG00000029644
AT1G19790	ENSG00000109132	ENSMUSG00000024134
AT5G24110	ENSG00000136574	ENSMUSG00000002249
AT1G76870	ENSG00000106689	ENSMUSG00000051367
AT3G30530	ENSG00000134595	ENSMUSG00000020167
AT2G22750	ENSG00000064195	ENSMUSG00000005718
AT2G18550	ENSG00000083307	ENSMUSG00000045680
AT3G01220	ENSG00000105392	ENSMUSG00000027985
AT1G22640	ENSG00000110693	ENSMUSG00000030551
AT1G31020	ENSG00000009709	ENSMUSG00000038692
AT5G10030	ENSG00000162367	ENSMUSG00000034460
AT3G45610	ENSG00000181449	ENSMUSG00000000782
AT4G21080	ENSG00000100146	ENSMUSG00000052271
AT3G21890	ENSG00000104447	ENSMUSG00000022761
AT3G01470	ENSG00000171223	ENSMUSG00000031326
AT4G21040	ENSG00000005889	ENSMUSG00000063659
AT1G51700	ENSG00000164736	ENSMUSG00000053477
AT5G02460	ENSG00000162772	ENSMUSG00000031162
AT5G47370	ENSG00000156127	ENSMUSG00000015053
AT2G28810	ENSG00000170345	ENSMUSG00000019900
AT3G12130	ENSG00000133937	ENSMUSG00000027967
AT1G70920	ENSG00000006194	ENSMUSG00000021944
AT3G55370	ENSG00000124766	ENSMUSG00000001510
AT5G62320	ENSG00000069011	ENSMUSG00000007805
AT3G50410	ENSG00000075426	ENSMUSG00000002393
AT3G12820	ENSG00000175592	ENSMUSG00000073043
AT4G38000	ENSG00000125398	ENSMUSG00000000861
AT5G10280	ENSG00000107485	ENSMUSG00000035187
AT3G52440	ENSG00000129194	ENSMUSG00000015619
AT5G63260	ENSG00000131668	ENSMUSG00000029844
AT1G14687	ENSG00000221818	ENSMUSG00000044167
AT2G46590	ENSG00000179348	ENSMUSG00000029026

AT5G62940	ENSG00000141448	ENSMUSG00000042622
AT1G26960	ENSG00000128714	ENSMUSG00000018698
AT4G26030	ENSG00000102145	ENSMUSG00000038679
AT5G06960	ENSG00000090447	ENSMUSG00000041483
AT1G22070	ENSG00000164330	ENSMUSG00000009471
AT5G65410	ENSG00000122180	ENSMUSG00000034701
AT1G36060	ENSG00000139515	ENSMUSG00000039830
AT1G79180	ENSG00000111249	ENSMUSG00000079509
AT3G28920	ENSG00000138795	ENSMUSG00000000435
AT2G37590	ENSG00000257923	ENSMUSG00000031627
AT3G61150	ENSG00000156925	ENSMUSG00000021506
AT4G25480	ENSG00000130726	ENSMUSG00000048756
AT1G69570	ENSG00000074047	ENSMUSG00000023034
AT5G66940	ENSG00000106031	ENSMUSG00000021356
AT2G18350	ENSG00000169856	ENSMUSG00000032368
AT5G60850	ENSG00000152977	ENSMUSG00000039275
AT1G34670	ENSG00000108001	ENSMUSG00000059552
AT1G16490	ENSG00000083844	ENSMUSG00000056493
AT4G00730	ENSG00000118689	ENSMUSG00000067860
AT1G75240	ENSG00000131061	ENSMUSG00000014704
AT1G77200	ENSG00000087510	ENSMUSG00000037025
AT2G23340	ENSG00000123405	ENSMUSG00000040891
AT4G28140	ENSG00000136535	ENSMUSG00000032228
AT3G10580	ENSG00000131264	ENSMUSG00000057098
AT1G68320	ENSG00000078900	ENSMUSG00000005893
AT3G50260	ENSG00000180806	ENSMUSG00000015846
AT4G25490	ENSG00000071564	ENSMUSG00000024563
AT1G64620	ENSG00000186350	ENSMUSG00000000938
AT3G12720	ENSG00000139352	ENSMUSG00000030067
AT3G47500	ENSG00000147789	ENSMUSG00000022508
AT1G71450	ENSG00000140262	ENSMUSG00000042499
AT1G46768	ENSG00000141510	ENSMUSG00000024515
AT3G30210	ENSG00000156273	ENSMUSG00000028640
AT3G16280	ENSG00000181315	ENSMUSG00000031965
AT4G16750	ENSG00000137203	ENSMUSG00000037992
AT5G67190	ENSG00000141956	ENSMUSG00000020538
AT1G06180	ENSG00000170954	ENSMUSG00000029646
AT5G14340	ENSG00000106571	ENSMUSG00000018899
AT4G01680	ENSG00000170577	ENSMUSG00000057551
AT1G22810	ENSG00000198740	ENSMUSG00000054715
AT4G17460	ENSG00000164107	ENSMUSG00000038210
AT1G29160	ENSG00000125798	ENSMUSG00000031870
AT1G68550	ENSG00000169957	ENSMUSG00000049604
AT4G17785	ENSG00000162992	ENSMUSG00000000567

AT4G25470	ENSG00000148737	ENSMUSG00000002147
AT4G32800	ENSG00000113916	ENSMUSG00000001419
AT2G31220	ENSG00000141568	ENSMUSG00000021359
AT1G25340	ENSG00000118495	ENSMUSG00000022529
AT5G25810	ENSG00000116833	ENSMUSG00000026459
AT1G19210	ENSG00000118526	ENSMUSG00000062175
AT1G67260	ENSG00000111049	ENSMUSG00000057982
AT2G35700	ENSG00000182742	ENSMUSG00000033006
AT3G06490	ENSG00000196628	ENSMUSG00000075595
AT2G44940	ENSG00000168267	ENSMUSG00000022053
AT5G07310	ENSG00000126778	ENSMUSG00000047407
AT3G28910	ENSG00000081059	ENSMUSG00000032238
AT3G60490	ENSG00000233608	ENSMUSG00000045179
AT1G52150	ENSG00000163508	ENSMUSG00000017950
AT1G12610	ENSG00000105991	ENSMUSG00000026380
AT1G14580	ENSG00000136870	ENSMUSG00000022383
AT1G12630	ENSG00000147596	ENSMUSG00000050368
AT4G36900	ENSG00000197905	ENSMUSG00000052942
AT3G53200	ENSG00000170325	ENSMUSG00000026976
AT1G28160	ENSG00000187079	ENSMUSG00000042812
AT4G31060	ENSG00000186951	ENSMUSG00000024955
AT5G19790	ENSG00000132170	ENSMUSG00000021779
AT5G54230	ENSG00000064835	ENSMUSG00000022510
AT5G51990	ENSG00000172238	ENSMUSG00000028150
AT4G06746	ENSG00000180535	ENSMUSG00000007946
AT2G02080	ENSG00000205927	ENSMUSG00000033669
AT3G47600	ENSG00000178403	ENSMUSG00000041578
AT1G53170	ENSG00000116044	ENSMUSG00000052435
AT4G28110	ENSG00000073282	ENSMUSG00000035799
AT4G24020	ENSG00000106261	ENSMUSG00000071661
AT1G47655	ENSG00000082175	ENSMUSG00000038255
AT3G15210	ENSG00000198517	ENSMUSG00000042477
AT5G64750	ENSG00000143006	ENSMUSG00000022484
AT1G74650	ENSG00000007372	ENSMUSG00000067261
AT2G45680	ENSG00000129152	ENSMUSG00000020160
AT1G43160	ENSG00000117395	ENSMUSG00000043602
AT2G33710	ENSG00000182318	ENSMUSG00000106723
AT5G18560	ENSG00000111424	ENSMUSG00000034538
AT1G30490	ENSG00000105996	ENSMUSG00000027434
AT5G61600	ENSG00000282757	ENSMUSG00000047371
AT5G25190	ENSG00000074219	ENSMUSG00000047638
AT1G53910	ENSG00000196482	ENSMUSG00000056501
AT2G42280	ENSG00000137090	ENSMUSG00000025958
AT3G20310	ENSG00000101076	ENSMUSG00000026222

AT1G51140	ENSG00000180787	ENSMUSG00000051038
AT2G02070	ENSG00000204366	ENSMUSG00000040632
AT1G77640	ENSG00000170485	ENSMUSG00000001823
AT5G16560	ENSG00000085274	ENSMUSG00000030199
AT5G51190	ENSG00000183770	ENSMUSG00000033016
AT1G04880	ENSG00000119715	ENSMUSG00000057156
AT1G21910	ENSG00000100625	ENSMUSG00000015605
AT1G44830	ENSG00000197372	ENSMUSG00000051817
AT1G35460	ENSG00000167555	ENSMUSG00000038151
AT1G28370	ENSG00000170608	ENSMUSG00000025902
AT2G43000	ENSG00000122691	ENSMUSG00000041287
AT2G44840	ENSG00000129514	ENSMUSG00000074637
AT3G18990	ENSG00000187140	ENSMUSG00000051910
AT5G07680	ENSG00000057657	ENSMUSG00000076431
AT4G34410	ENSG00000137265	ENSMUSG00000020679
AT3G60580	ENSG00000275410	ENSMUSG00000029556
AT3G11020	ENSG00000103241	ENSMUSG00000035451
AT1G27730	ENSG00000184486	ENSMUSG00000001517
AT2G38340	ENSG00000162599	ENSMUSG00000050397
AT1G50640	ENSG00000114439	ENSMUSG00000021095
AT3G08500	ENSG00000143842	ENSMUSG00000048502
AT5G39820	ENSG00000170561	ENSMUSG00000021848
AT1G74480	ENSG00000106536	ENSMUSG00000024837
AT5G22990	ENSG00000180532	ENSMUSG00000074220
AT5G13910	ENSG00000167034	ENSMUSG00000028610
AT2G02820	ENSG00000176887	ENSMUSG00000043013
AT4G27950	ENSG00000148516	ENSMUSG00000042496
AT1G01250	ENSG00000184895	ENSMUSG00000015627
AT4G18880	ENSG00000130700	ENSMUSG00000070643
AT2G41690	ENSG00000064218	ENSMUSG00000027684
AT5G44210	ENSG00000151090	ENSMUSG00000021972
AT3G02150	ENSG00000168066	ENSMUSG00000079033
AT5G62000	ENSG00000164256	ENSMUSG00000002996
AT3G45170	ENSG00000164532	ENSMUSG00000025959
AT1G72050	ENSG00000164458	ENSMUSG00000014039
AT5G47230	ENSG00000170631	ENSMUSG00000029705
AT1G06850	ENSG00000130803	ENSMUSG00000019564
AT2G06200	ENSG00000165556	ENSMUSG00000078302
AT2G01940	ENSG00000166888	ENSMUSG00000042372
AT5G65130	ENSG00000177463	ENSMUSG00000037674
AT4G28520	ENSG00000125618	ENSMUSG00000028042
At3g12730	ENSG00000135100	ENSMUSG00000022286
AT4G18450	ENSG00000182968	ENSMUSG00000018263
AT1G03800	ENSG00000039600	ENSMUSG00000047036

AT5G17800	ENSG00000160685	ENSMUSG00000026610
AT3G23220	ENSG00000196767	ENSMUSG00000035033
AT5G62380	ENSG00000012504	ENSMUSG00000091736
AT3G11280	ENSG00000204103	ENSMUSG00000049823
AT1G43700	ENSG00000143032	ENSMUSG00000029563
AT5G05790	ENSG00000161940	ENSMUSG00000034384
AT1G68670	ENSG00000083817	ENSMUSG00000037730
AT1G06070	ENSG00000162924	ENSMUSG00000001815
AT1G13300	ENSG00000141905	ENSMUSG00000062327
AT3G20770	ENSG00000107249	ENSMUSG00000030699
AT3G60530	ENSG00000109705	ENSMUSG00000026735
AT1G75490	ENSG00000068305	ENSMUSG00000009248
AT5G10120	ENSG00000089225	ENSMUSG00000055210
AT4G20440	ENSG00000185551	ENSMUSG00000048385
AT2G20400	ENSG00000081189	ENSMUSG00000015579
AT1G24250	ENSG00000160113	ENSMUSG00000096014
AT5G52170	ENSG00000025434	ENSMUSG00000026398
AT5G08070	ENSG00000170178	ENSMUSG00000020052
AT2G22540	ENSG00000213999	ENSMUSG00000026751
AT5G25830	ENSG00000126351	ENSMUSG00000059842
AT3G23240	ENSG00000185022	ENSMUSG00000039634
AT1G32240	ENSG00000119866	ENSMUSG00000022676
AT5G47220	ENSG00000131759	ENSMUSG00000022330
AT5G29000	ENSG00000196646	ENSMUSG00000054310
AT2G40620	ENSG00000126368	ENSMUSG00000042414
AT1G08010	ENSG00000118263	ENSMUSG00000046470
AT4G36160	ENSG00000258873	ENSMUSG00000063889
AT5G58900	ENSG00000136327	ENSMUSG00000009733
AT1G72360	ENSG00000198911	ENSMUSG00000005583
AT2G45660	ENSG00000185122	ENSMUSG00000042063
AT1G65620	ENSG00000008196	ENSMUSG00000030557
AT2G33810	ENSG00000175197	ENSMUSG00000038253
AT3G46590	ENSG00000197279	ENSMUSG00000027168
AT1G28470	ENSG00000171163	ENSMUSG00000039377
AT1G15750	ENSG00000101883	ENSMUSG00000027544
AT1G76490	ENSG00000025156	ENSMUSG00000005917
AT5G67450	ENSG00000121075	ENSMUSG00000022061
AT1G13450	ENSG00000179456	ENSMUSG00000044220
AT1G14510	ENSG00000143365	ENSMUSG00000038203
AT1G42990	ENSG00000069667	ENSMUSG00000058756
AT3G21270	ENSG00000161298	ENSMUSG00000046351
AT5G18830	ENSG00000164463	ENSMUSG00000044542
AT4G01500	ENSG00000129535	ENSMUSG00000019817
AT2G45480	ENSG00000102908	ENSMUSG00000012520

AT1G31140	ENSG00000173153	ENSMUSG00000021381
AT3G50060	ENSG00000085276	ENSMUSG00000034957
AT5G25475	ENSG00000105856	ENSMUSG00000031902
AT1G51220	ENSG00000147421	ENSMUSG00000008398
AT4G30080	ENSG00000177374	ENSMUSG00000026826
AT3G26790	ENSG00000278570	ENSMUSG00000029729
AT1G76880	ENSG00000198081	ENSMUSG00000037447
AT5G08750	ENSG00000172216	ENSMUSG00000001496
AT4G01280	ENSG00000116017	ENSMUSG00000025323
AT2G41835	ENSG00000125347	ENSMUSG00000055053
AT2G03500	ENSG00000213928	ENSMUSG00000028707
AT1G27360	ENSG00000126456	ENSMUSG00000044807
AT2G46830	ENSG00000181827	ENSMUSG00000009739
AT1G13260	ENSG00000119919	ENSMUSG00000040148
AT4G18770	ENSG00000126746	ENSMUSG00000028736
AT3G52170	ENSG00000183072	ENSMUSG00000043099
AT3G06740	ENSG00000245848	ENSMUSG00000051977
AT1G51600	ENSG00000099326	ENSMUSG00000032292
AT5G11510	ENSG00000196843	ENSMUSG00000043969
AT4G32730	ENSG00000165702	ENSMUSG00000018983
AT4G00250	ENSG00000142599	ENSMUSG00000021318
AT2G28920	ENSG00000215271	ENSMUSG00000040726
AT3G10113	ENSG00000164011	ENSMUSG00000028565
AT1G63030	ENSG00000125820	ENSMUSG00000029275
AT5G56840	ENSG00000101216	ENSMUSG00000026872
AT5G52660	ENSG00000112333	ENSMUSG00000022479
AT2G27380	ENSG00000125817	ENSMUSG00000036602
AT1G01060	ENSG00000185670	ENSMUSG00000024238
AT5G17300	ENSG00000116604	ENSMUSG00000004842
AT5G47390	ENSG00000149922	ENSMUSG00000030380
AT1G74840	ENSG00000117595	ENSMUSG00000026468
AT3G09600	ENSG00000134138	ENSMUSG00000054272
AT5G61620	ENSG00000008441	ENSMUSG00000045268
AT2G32460	ENSG00000189308	ENSMUSG00000035923
AT5G08520	ENSG00000116819	ENSMUSG00000021362
AT3G10030	ENSG00000073861	ENSMUSG00000038193
AT1G19000	ENSG00000140044	ENSMUSG00000001444
AT1G60920	ENSG00000175322	ENSMUSG00000037243
AT5G58850	ENSG00000049768	ENSMUSG00000032446
AT3G24050	ENSG00000176678	ENSMUSG00000001288
AT1G49010	ENSG00000198176	ENSMUSG00000002983
AT3G04030	ENSG00000183734	ENSMUSG00000021255
AT2G01060	ENSG00000129071	ENSMUSG00000026077
AT3G11440	ENSG00000198815	ENSMUSG00000049691

AT2G31230	ENSG00000067066	ENSMUSG00000048402
AT4G36540	ENSG00000173039	ENSMUSG00000024497
AT3G60820	ENSG00000128604	ENSMUSG00000021685
AT2G15890	ENSG00000137270	ENSMUSG00000020037
AT2G33550	ENSG00000123407	ENSMUSG00000063632
AT5G67580	ENSG00000020633	ENSMUSG00000042472
AT4G37180	ENSG00000135638	ENSMUSG00000049672
AT5G13180	ENSG00000117707	ENSMUSG00000032119
AT2G24430	ENSG00000278129	ENSMUSG00000029249
AT3G57600	ENSG00000116035	
AT4G14465	ENSG00000153234	
AT1G28300	ENSG00000188295	
	ENSG00000178860	
	ENSG00000072736	
	ENSG00000163064	
	ENSG00000174279	
	ENSG00000108788	
	ENSG00000215612	
	ENSG00000230797	
	ENSG00000124827	
	ENSG00000164379	
	ENSG00000105866	
	ENSG00000134532	
	ENSG00000111046	
	ENSG00000084093	

Supplementary Table S4. Homology analysis of cell-fate determining TFs involved in early embryogenesis/cell cycle

**hsa**	**mmu**	e.val	**dme**	e.val	**cel**	e.val	**ath**	e.val	**sce**	e.val
POU5F1	*Pou5f1*	0	*vvl*	4e-54	*unc-86*	1e-34	*HDG11*	6e-04	*YOX1*	8e-04
TCF3	*Tcf3*	0	da	1e-41	*hlh-2*	1e-24				
RBPJ	*Rbpj*	0	*Su(H)*	0	*lag-1*	6e-166				
SMAD2	*Smad2*	0	*Mad*	0	*sma-2*	5e-123				
SOX13	*Sox13*	0	*pan*	2e-10	*Sox102F*	9e-51	*HMGB6*	6e-06	*ROX1*	3e-08
GSC	*Gsc*	0	*Gsc*	8e-27	*ceh-45*	9e-23	*GL2*	2e-08	*YOX1*	3e-06
PITX1	*Pitx1*	0	*Ptx1*	1e-53	*unc-30*	2e-34	*HB-7*	9e-08	*PHO2*	4e-09
NR2F2	*Nr2f2*	0	*svp*	0	*unc-55*	1e-67				
ISL1	*Isl1*	0	*tup*	1e-135	*lim-7*	1e-65	*REV*	4e-07	*PXL1*	2e-07
MITF	*Mitf*	0	*Mitf*	1e-36	*hlh-30*	1e-29	*SPT*	1e-05	*RTG3*	2e-07
GATA6	*Gata6*	0	*pnr*	3e-59	*elt-1*	2e-50	*GATA17*	5e-05	*GAT1*	4e-17
KLF4	*Klf4*	0	*luna*	4e-43	*klf-1*	7e-44	*YY1*	4e-14	*AZF1*	9e-19

PRDM14	*Prdm14*	0	*su(Hw)*	2e-28	F47E1.3	1e-78	*JKD*	1e-04	*ZAP1*	1e-27
NANOG	*Nanog*	2e-102	*vnd*	2e-14	*tab-1*	2e-14	*HB-1*	2e-04	*YOX1*	5e-04
SOX2	*Sox2*	0	*Sox21a*	2e-49	*sox-2*	6e-52	*HMGB2*	2e-06	*ROX1*	4e-08
STAT3	*Stat3*	0	*Stat92E*	1e-39	*sta-1*	6e-36	*SHA*	1e-04		
TCF7L2	*Tcf7l2*	0	*pan*	3e-75	*pop-1*	3e-26			*ROX1*	2e-05
RARA	*Rara*	0	*Hr38*	6e-54	*nhr-69*	7e-30				
SMAD4	*Smad4*	0	*Med*	2e-114	*sma-4*	5e-50				
RUNX2	*Runx2*	0	*RunxA*	3e-64	*rnt-1*	8e-35				
SOX1	*Sox1*	0	*Sox21a*	6e-48	*sox-2*	1e-53	*HMGB2*	6e-05	*ROX1*	9e-07
ETV2	*Etv2*	0	*Ets65A*	2e-31	*ets-5*	3e-32				
PAX6	*Pax6*	0	*toy*	4e-134	*vab-3*	2e-123	*HB40*	8e-06	*PHO2*	2e-06
EOMES	*Eomes*	0	*byn*	2e-45	*mls-1*	5e-50				
ASCL1	*Ascl1*	0	*ac*	7e-17	*hlh-3*	4e-15	*bHLH38*	0.003		
FOXA1	*Foxa1*	0	*fkh*	1e-70	*pha-4*	2e-60			*HCM1*	5e-23
PAX7	*Pax7*	0	*prd*	3e-107	*vab-3*	2e-79	*HDG1*	6e-07	*YOX1*	6e-06
ELF5	*Elf5*	0	Eip74EF	5e-19	svh-5	4e-29				
POU1F1	*Pou1f1*	0	vvl	9e-59	*ceh-6*	3e-53	*HB6*	1e-05	*YHP1*	0.002
GATA2	*Gata2*	0	*grn*	4e-70	*elt-1*	2e-52	*GATA19*	1e-04	*GAT1*	2e-13
GATA3	*Gata3*	0	*grn*	7e-72	*elt-1*	3e-53	*GATA19*	5e-05	*GAT1*	2e-13
SOX17	*Sox17*	0	*Sox15*	1e-40	*sox-2*	2e-31	*HMGB2*	3e-07	*ROX1*	2e-06
GCM1	*Gcm1*	0	*gcm*	1e-56						
FOXA2	*Foxa2*	0	*fkh*	8e-76	*pha-4*	8e-59			*HCM1*	2e-22

Note: TFs that are identified in this study are in green.

Supplementary Figures

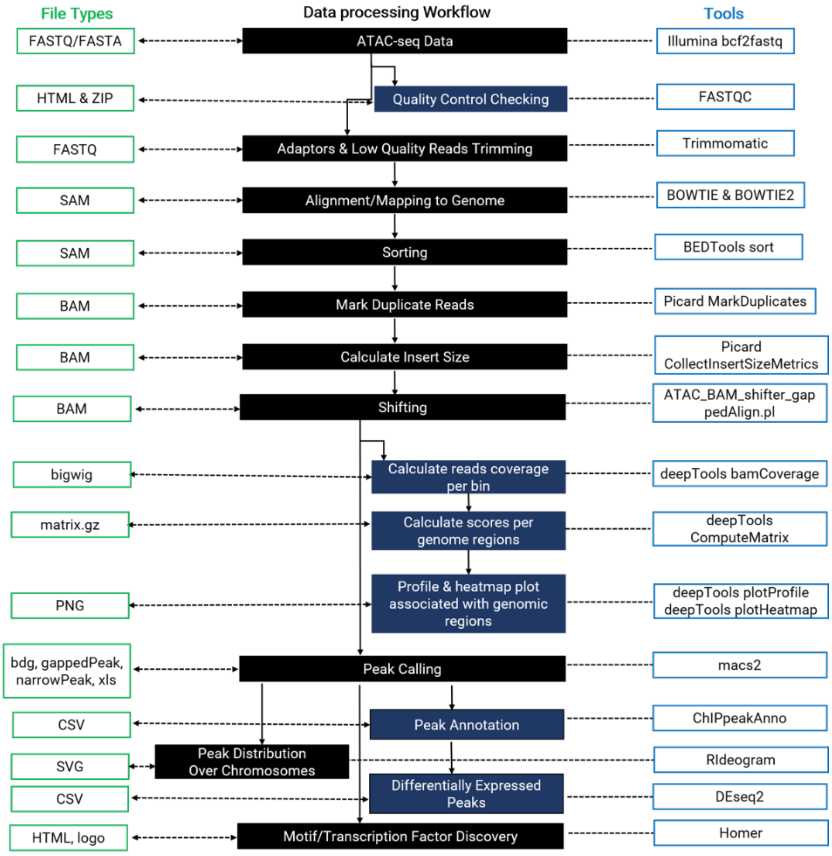

Figure S1. Unified pipeline for ATAC-seq data analysis.

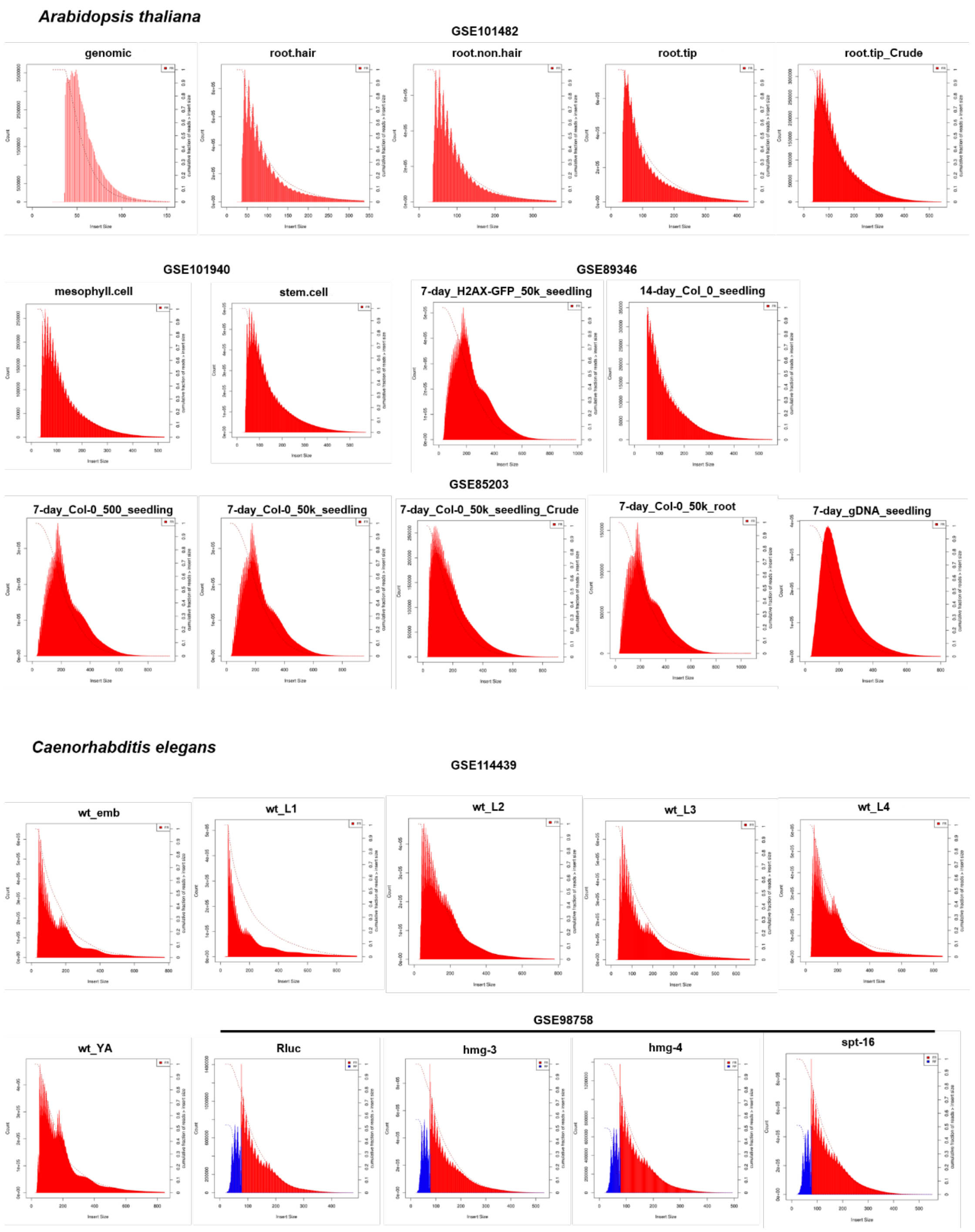

Arabidopsis thaliana
GSE101482
genomic
root.hair
root.non.hair
root.tip
root.tip_Crude
GSE101940
mesophyll.cell
stem.cell
GSE89346
7-day_H2AX-GFP_50k_seedling
14-day_Col_0_seedling
GSE85203
7-day_Col-0_500_seedling
7-day_Col-0_50k_seedling
7-day_Col-0_50k_seedling_Crude
7-day_Col-0_50k_root
7-day_gDNA_seedling
Caenorhabditis elegans
GSE114439
wt_emb
wt_L1
wt_L2
wt_L3
wt_L4
GSE98758
wt_YA
Rluc
hmg-3
hmg-4
spt-16

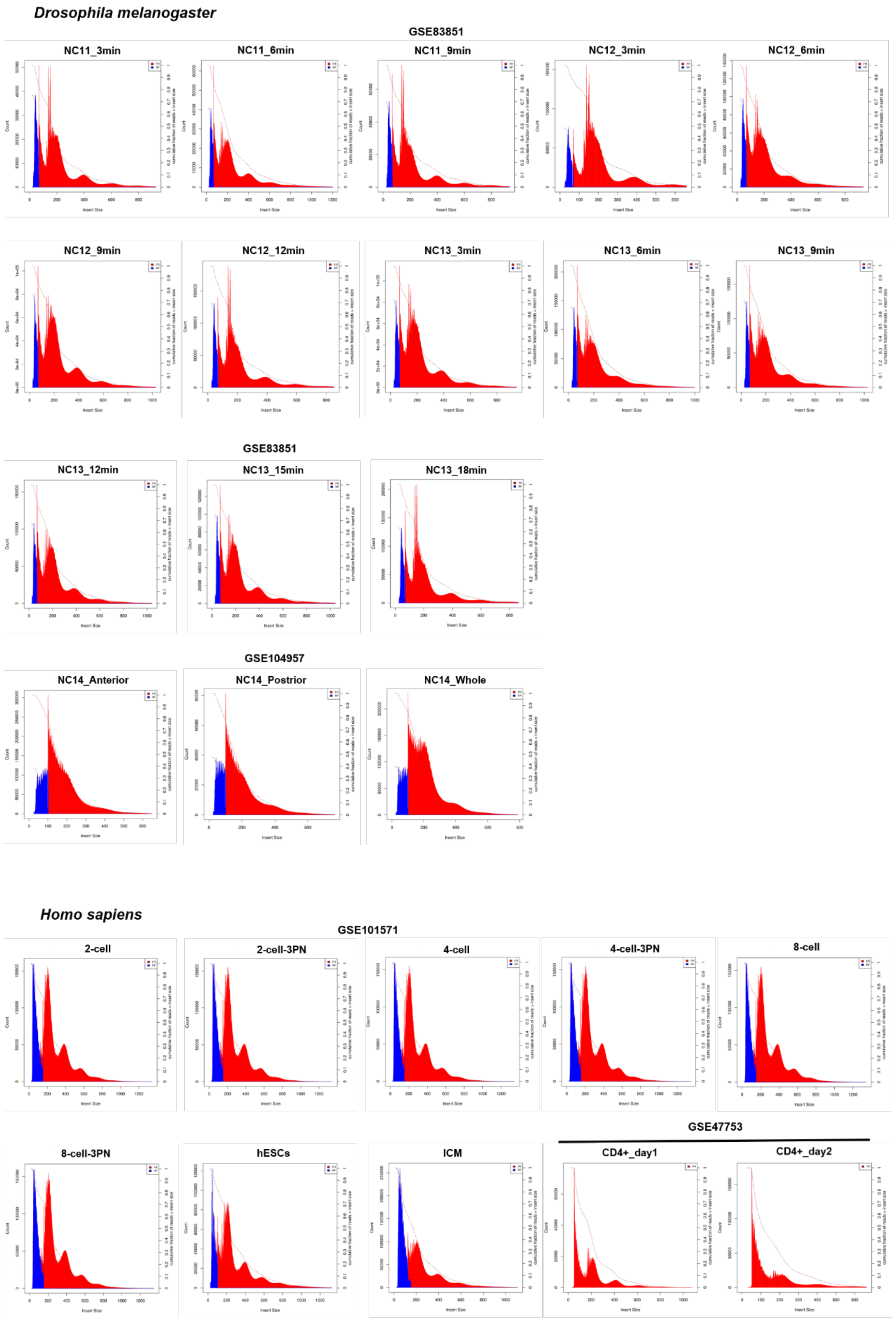
Drosophila melanogaster
GSE83851
NC11_3min
NC11_6min
NC11_9min
NC12_3min
NC12_6min
NC12_9min
NC12_12min
NC13_3min
NC13_6min
NC13_9min
GSE83851
NC13_12min
NC13_15min
NC13_18min
GSE104957
NC14_Anterior
NC14_Postrior
NC14_Whole
Homo sapiens
GSE101571
2-cell
2-cell-3PN
4-cell
4-cell-3PN
8-cell
8-cell-3PN
hESCs
ICM
GSE47753
CD4+_day1
CD4+_day2

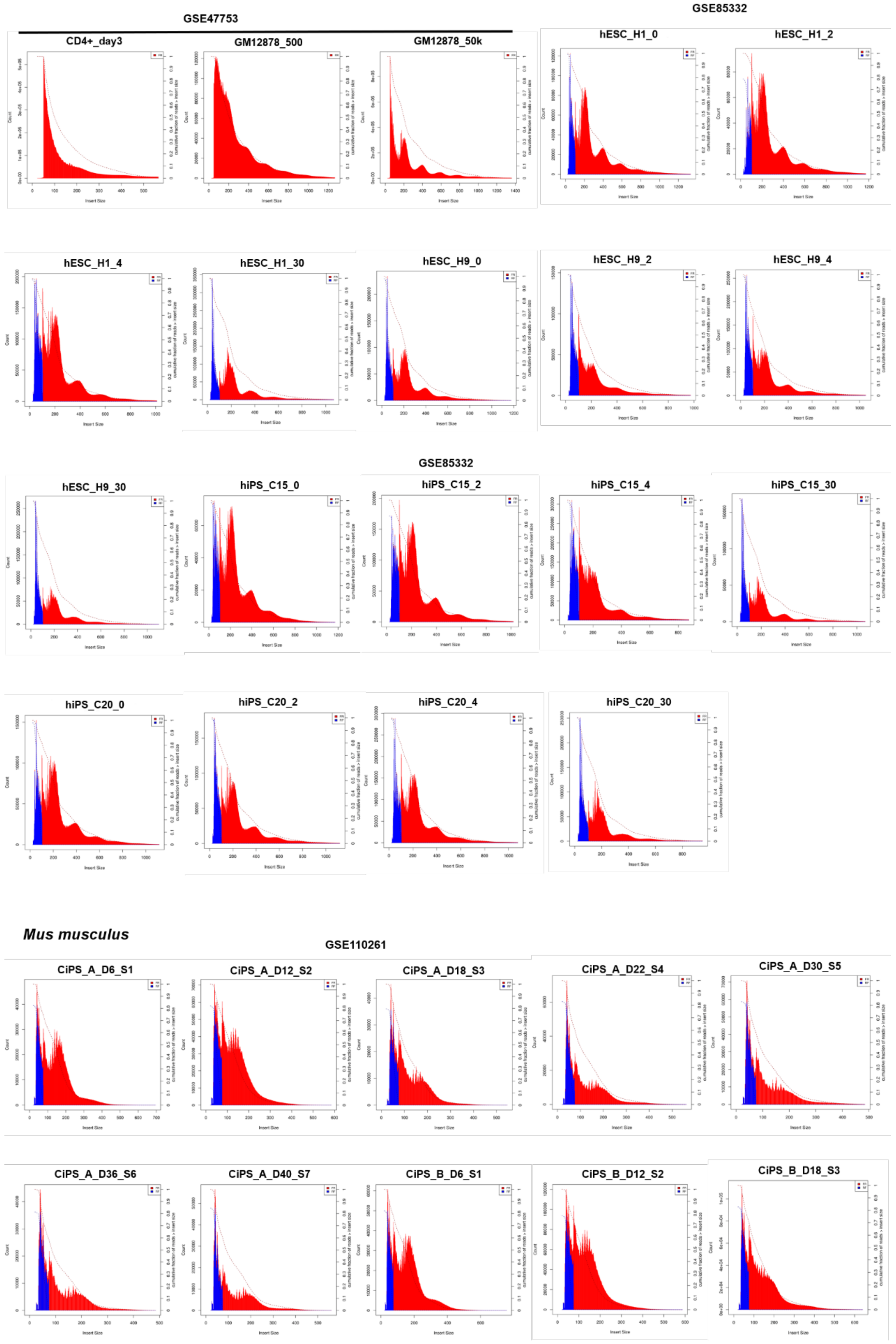
GSE47753
CD4+_day3
GM12878_500
GM12878_50k
GSE85332
hESC_H1_0
hESC_H1_2
hESC_H1_4
hESC_H1_30
hESC_H9_0
hESC_H9_2
hESC_H9_4
GSE85332
hESC_H9_30
hiPS_C15_0
hiPS_C15_2
hiPS_C15_4
hiPS_C15_30
hiPS_C20_0
hiPS_C20_2
hiPS_C20_4
hiPS_C20_30
Mus musculus
GSE110261
CiPS_A_D6_S1
CiPS_A_D12_S2
CiPS_A_D18_S3
CiPS_A_D22_S4
CiPS_A_D30_S5
CiPS_A_D36_S6
CiPS_A_D40_S7
CiPS_B_D6_S1
CiPS_B_D12_S2
CiPS_B_D18_S3
Insert Size
Count

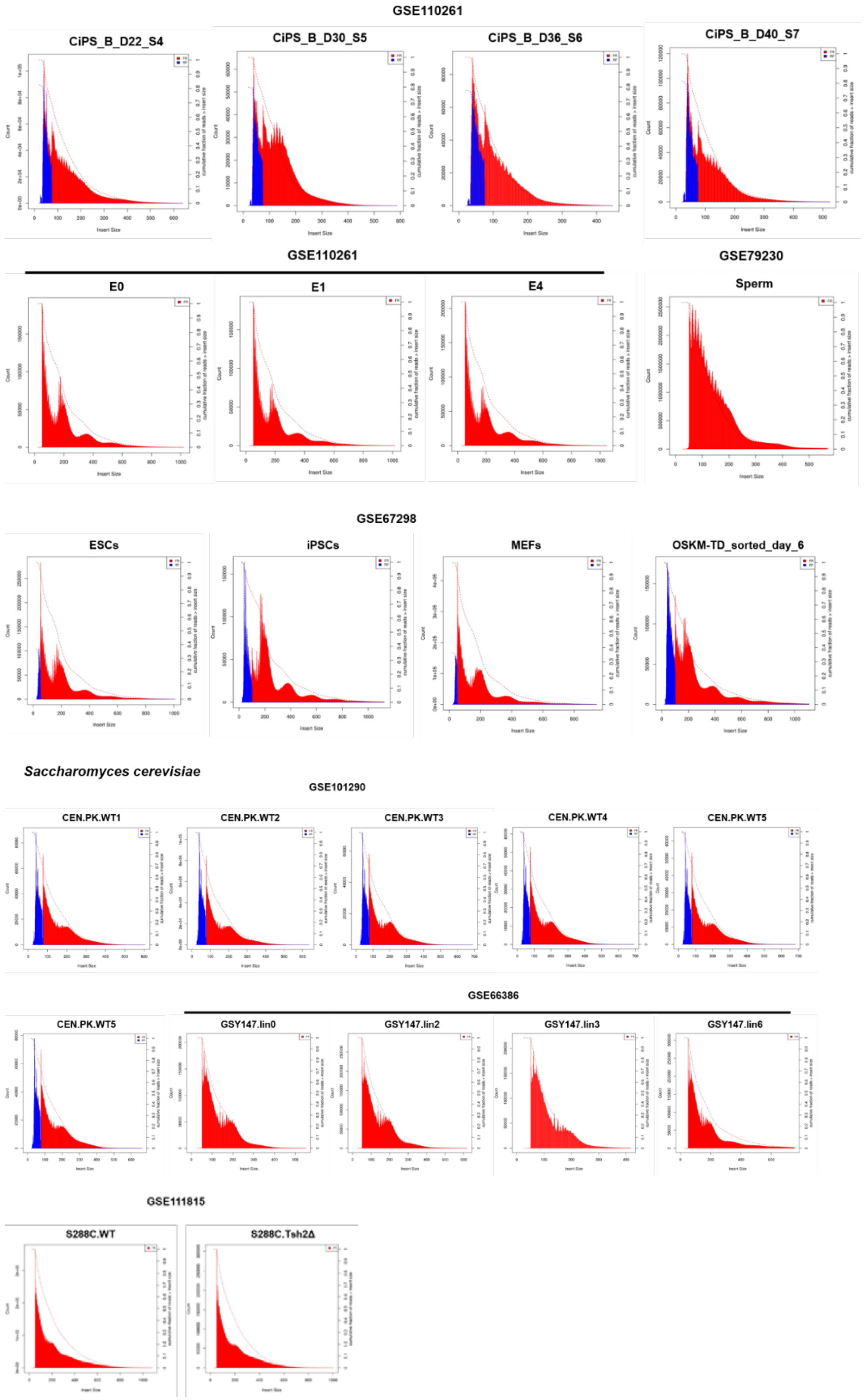

Figure S2. The insert size of ATAC-seq paired-end and single-end reads in the sample prepared from different developmental stages or different strains of six species. The dotted line indicates the trendline. The raw data were obtained from NCBI GEO accession.

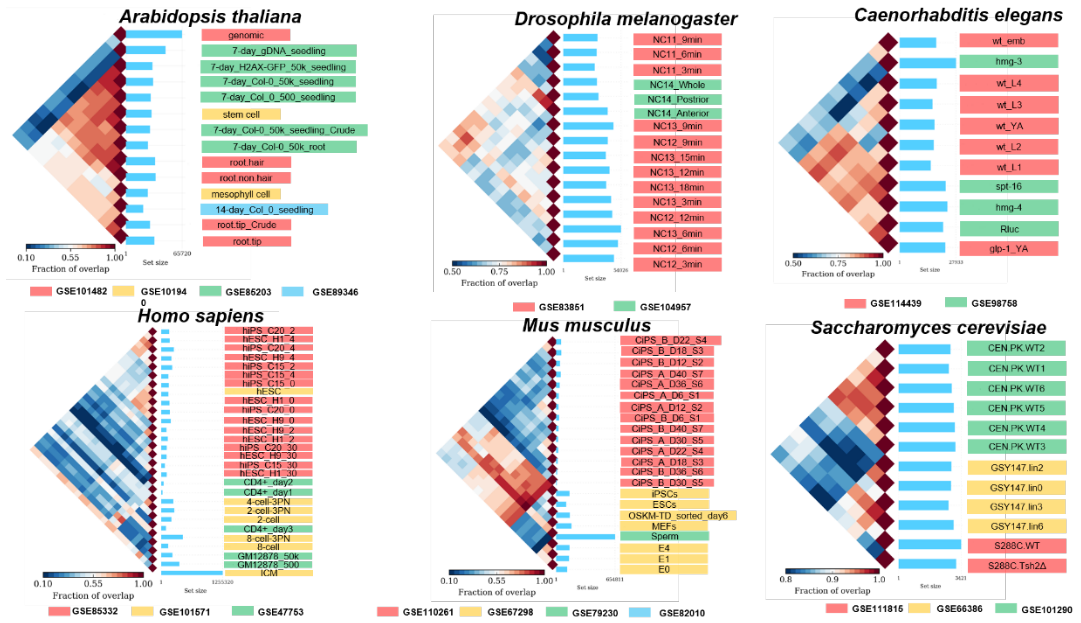

Figure S3. Pairwise analysis of peaks in each species. The left part of each figure demonstrates the overlapping rate between peaks in each sample, and the right part shows the peaks identified in all samples of each species.

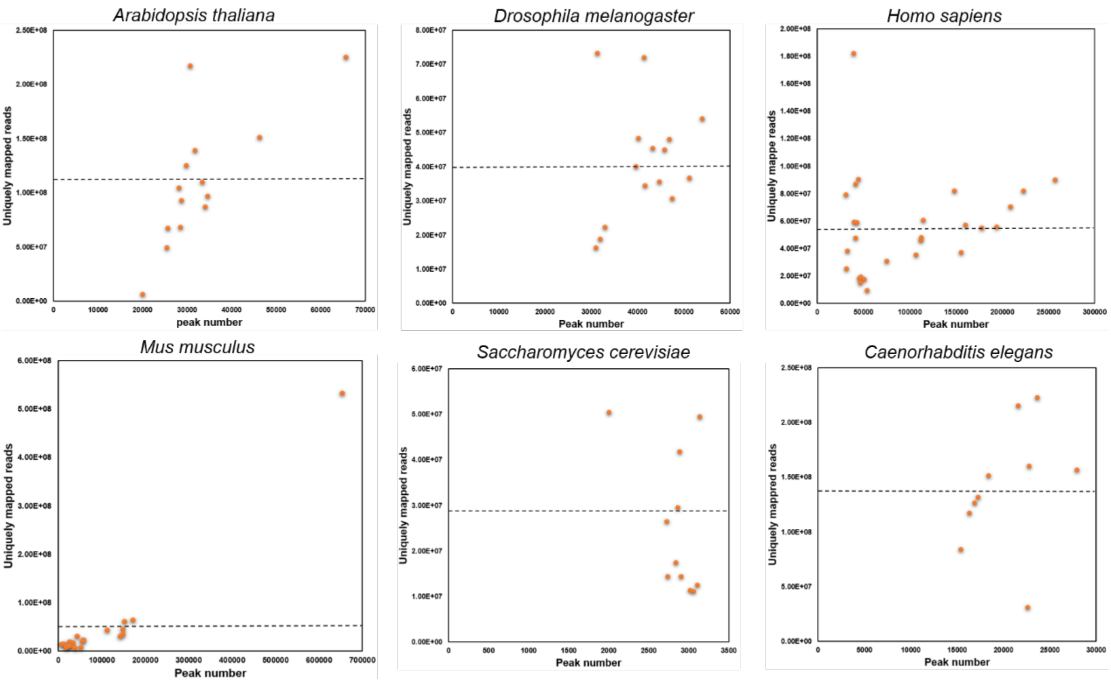

Figure S4. Point diagrams show the correlation of uniquely mapped reads with peak number. Peak number (x-axis) and uniquely mapped reads (y-axis) per ATAC-seq sample in six species (*Arabidopsis*, *Drosophila*, human, mouse, yeast, *C. elegans*). The dashed line indicates the mean reads number.

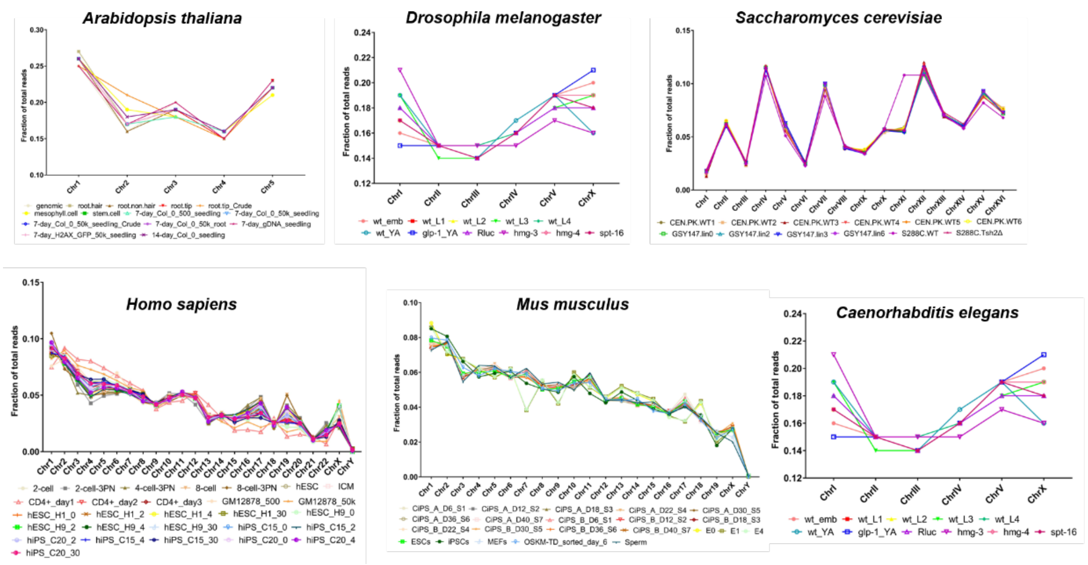

Figure S5. Reads distribution patterns over chromosomes. Chromosome (x-axis) and Fraction of total reads (y-axis) per sample in six species.

A. thaliana

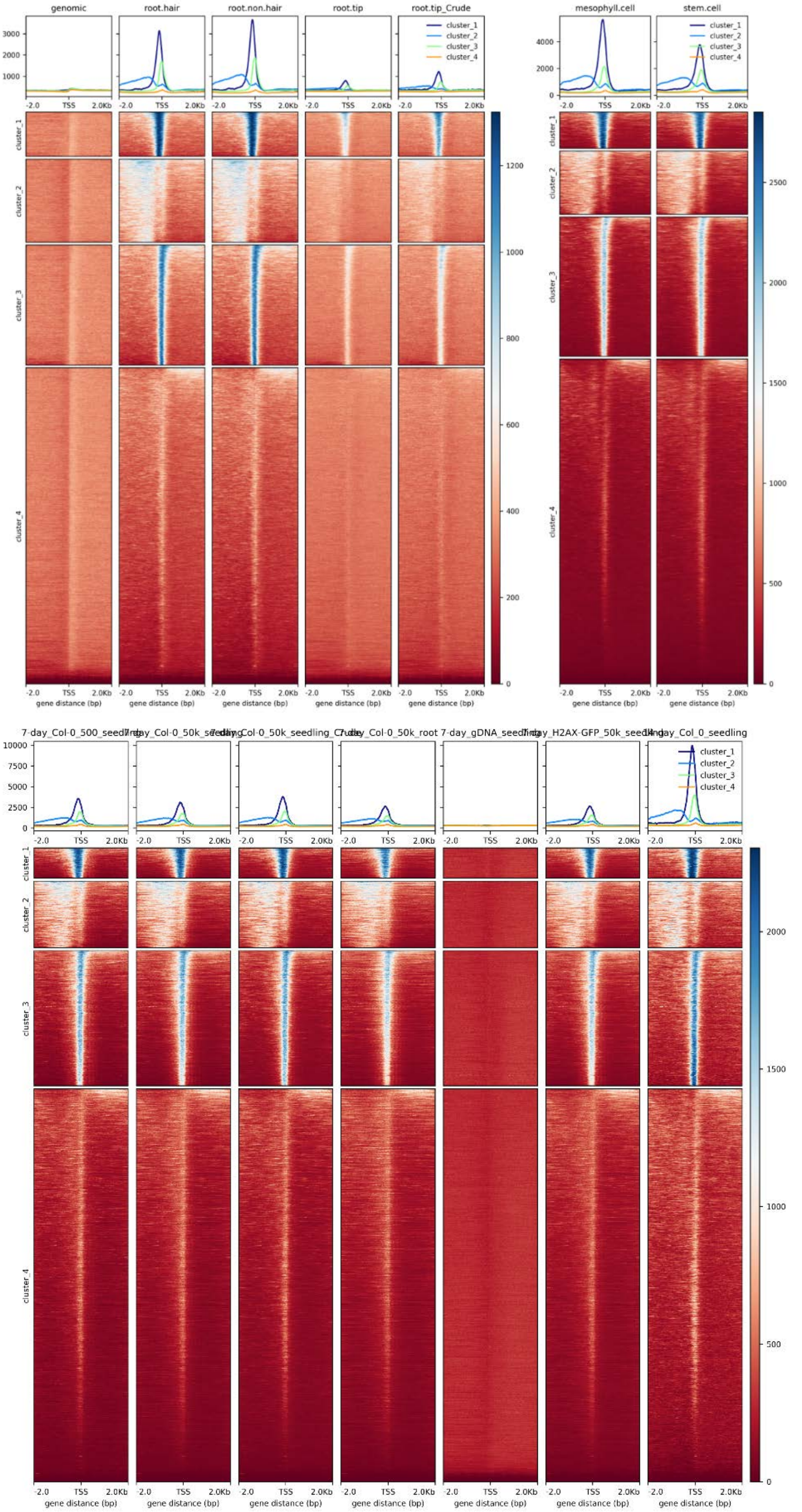
genomic
root.hair
root.non.hair
root.tip
root.tip_Crude
mesophyll.cell
stem.cell
cluster_1
cluster_2
cluster_3
cluster_4
-2.0
TSS
2.0Kb
gene distance (bp)
7-day_Col-0_50k_root
14-day_Col-0_seedling

C. elegans

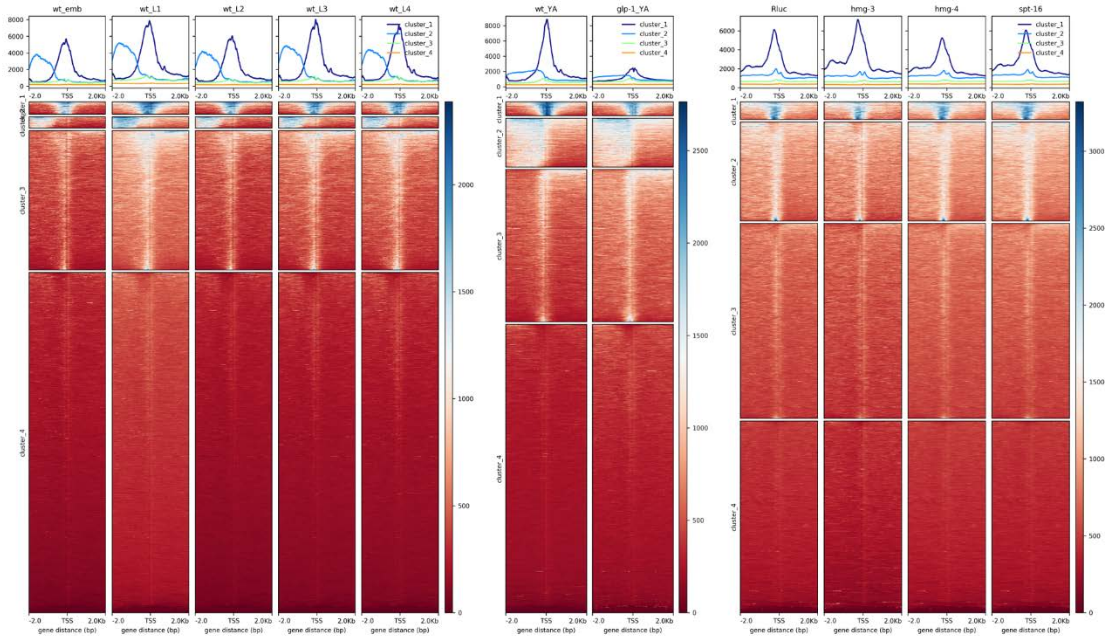

D. melanogaster

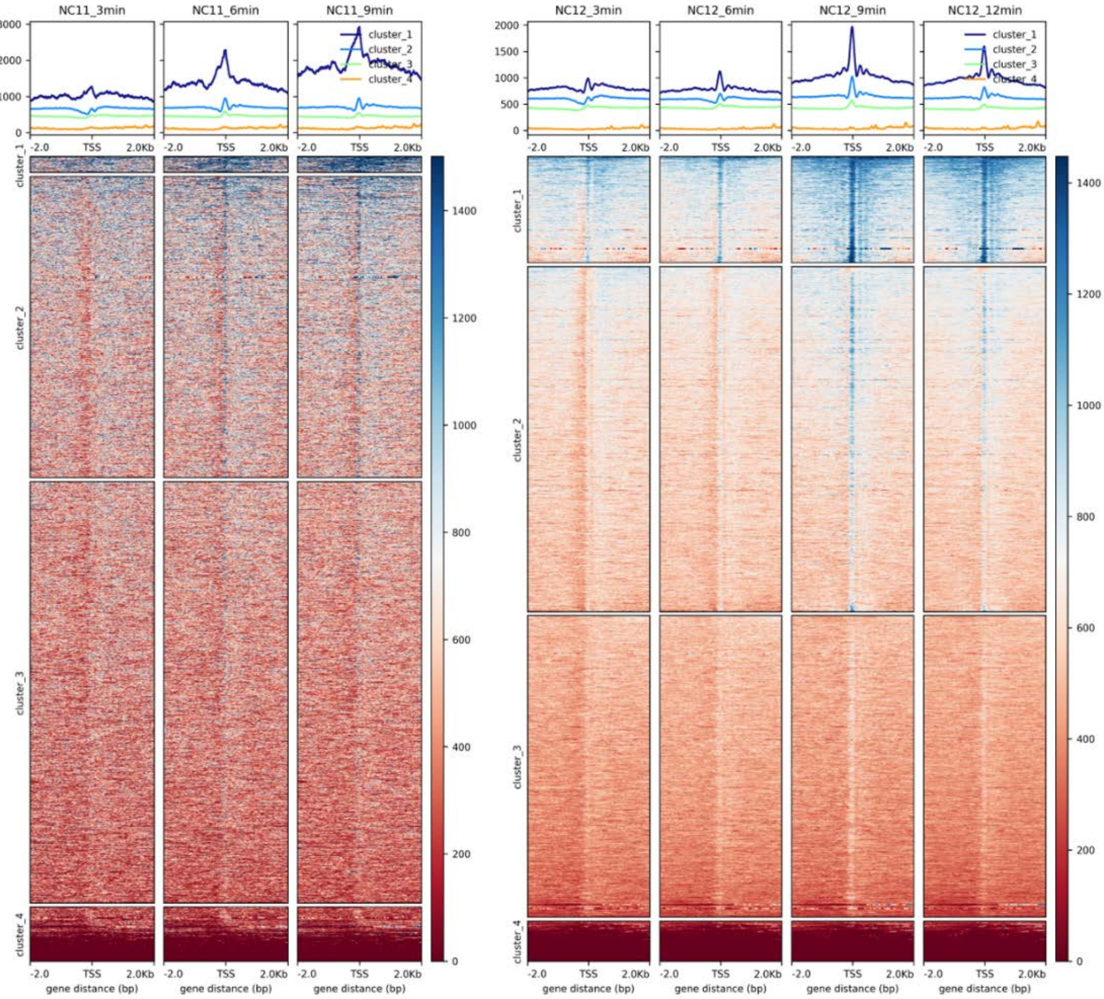

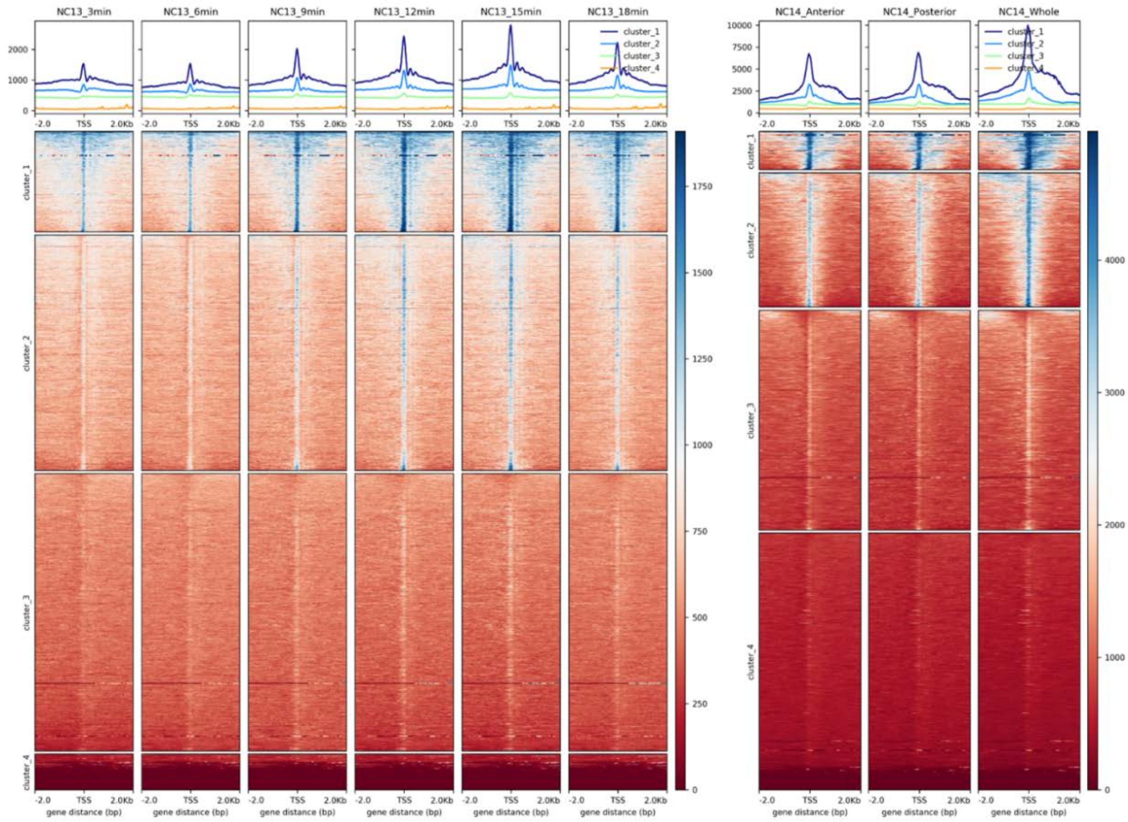

S. cerevisiae

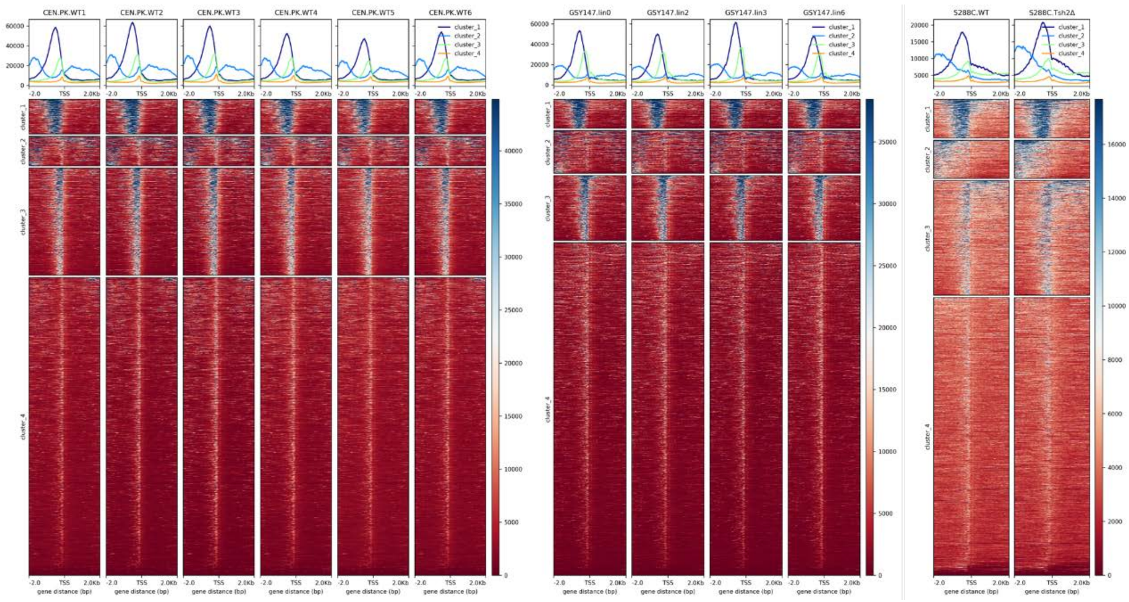

H. sapiens

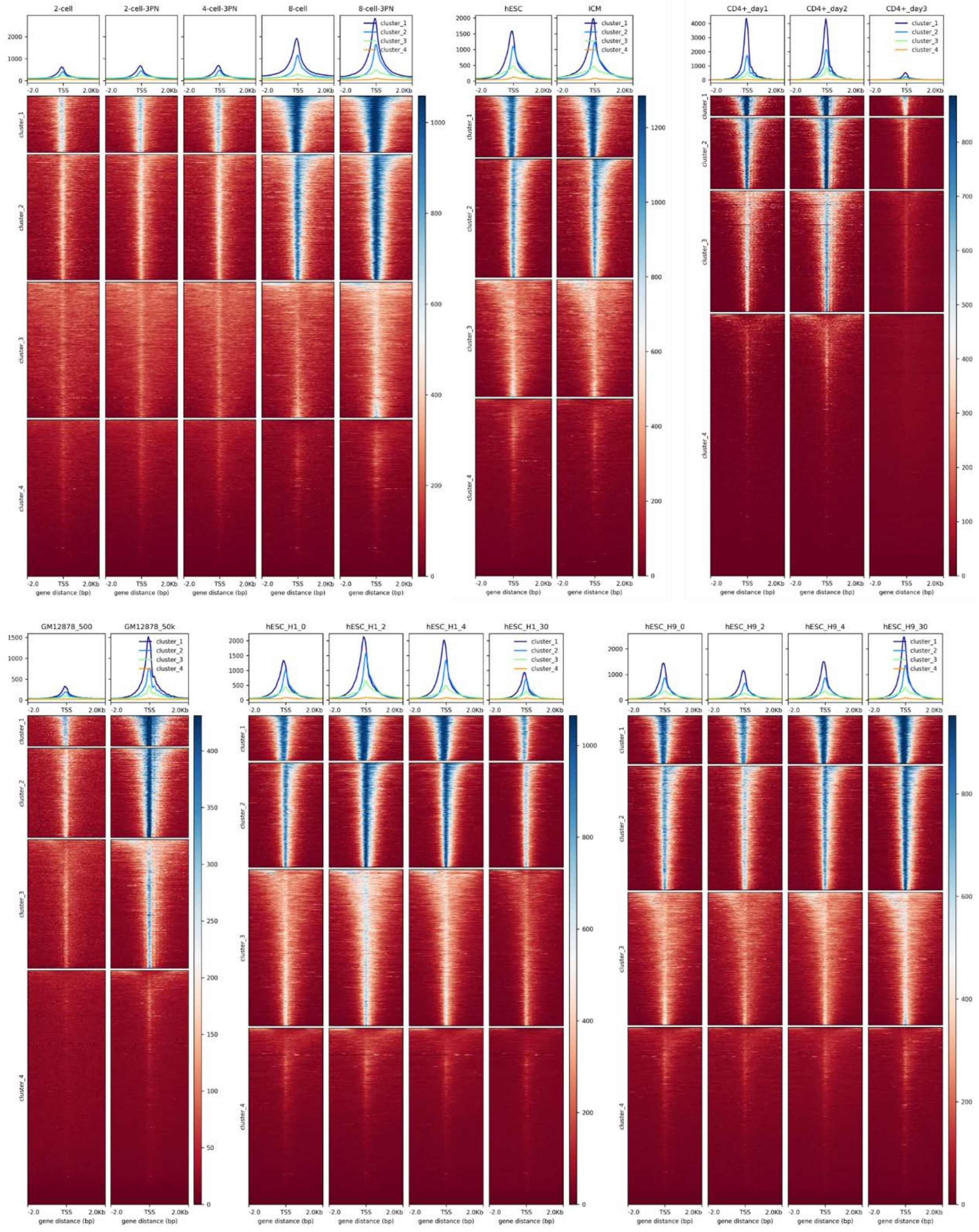

2-cell
2-cell-3PN
4-cell-3PN
8-cell
8-cell-3PN
hESC
ICM
CD4+_day1
CD4+_day2
CD4+_day3
GM12878_500
GM12878_50k
hESC_H1_0
hESC_H1_2
hESC_H1_4
hESC_H1_30
hESC_H9_0
hESC_H9_2
hESC_H9_4
hESC_H9_30
cluster_1
cluster_2
cluster_3
cluster_4
-2.0
TSS
2.0Kb
gene distance (bp)

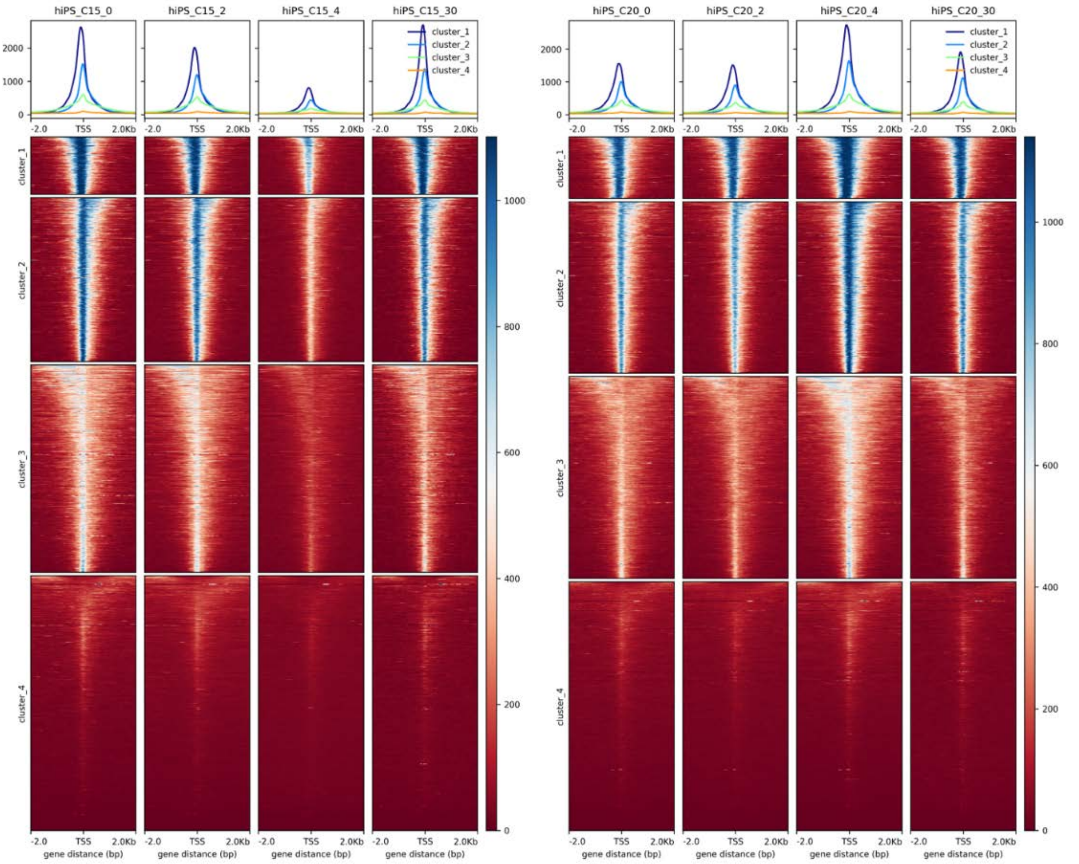
hiPS_C15_0
hiPS_C15_2
hiPS_C15_4
hiPS_C15_30
hiPS_C20_0
hiPS_C20_2
hiPS_C20_4
hiPS_C20_30
cluster_1
cluster_2
cluster_3
cluster_4
-2.0
TSS
2.0Kb
gene distance (bp)

M. musculus

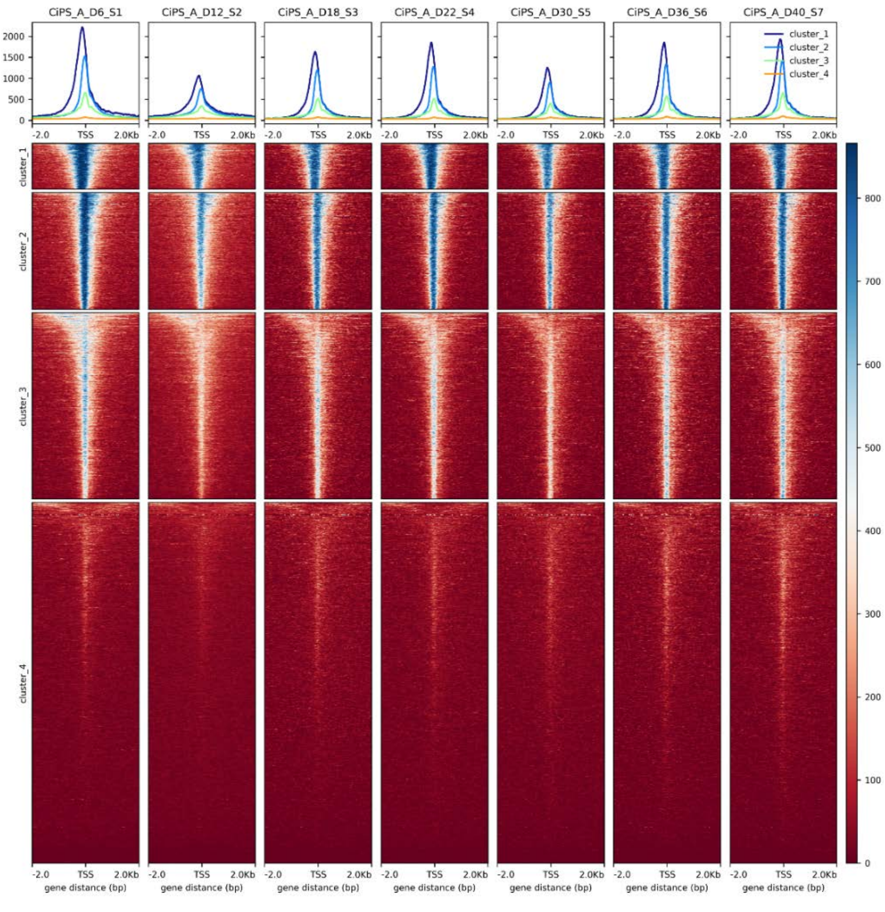
CiPS_A_D6_S1
CiPS_A_D12_S2
CiPS_A_D18_S3
CiPS_A_D22_S4
CiPS_A_D30_S5
CiPS_A_D36_S6
CiPS_A_D40_S7
cluster_1
cluster_2
cluster_3
cluster_4
-2.0
TSS
2.0Kb
gene distance (bp)

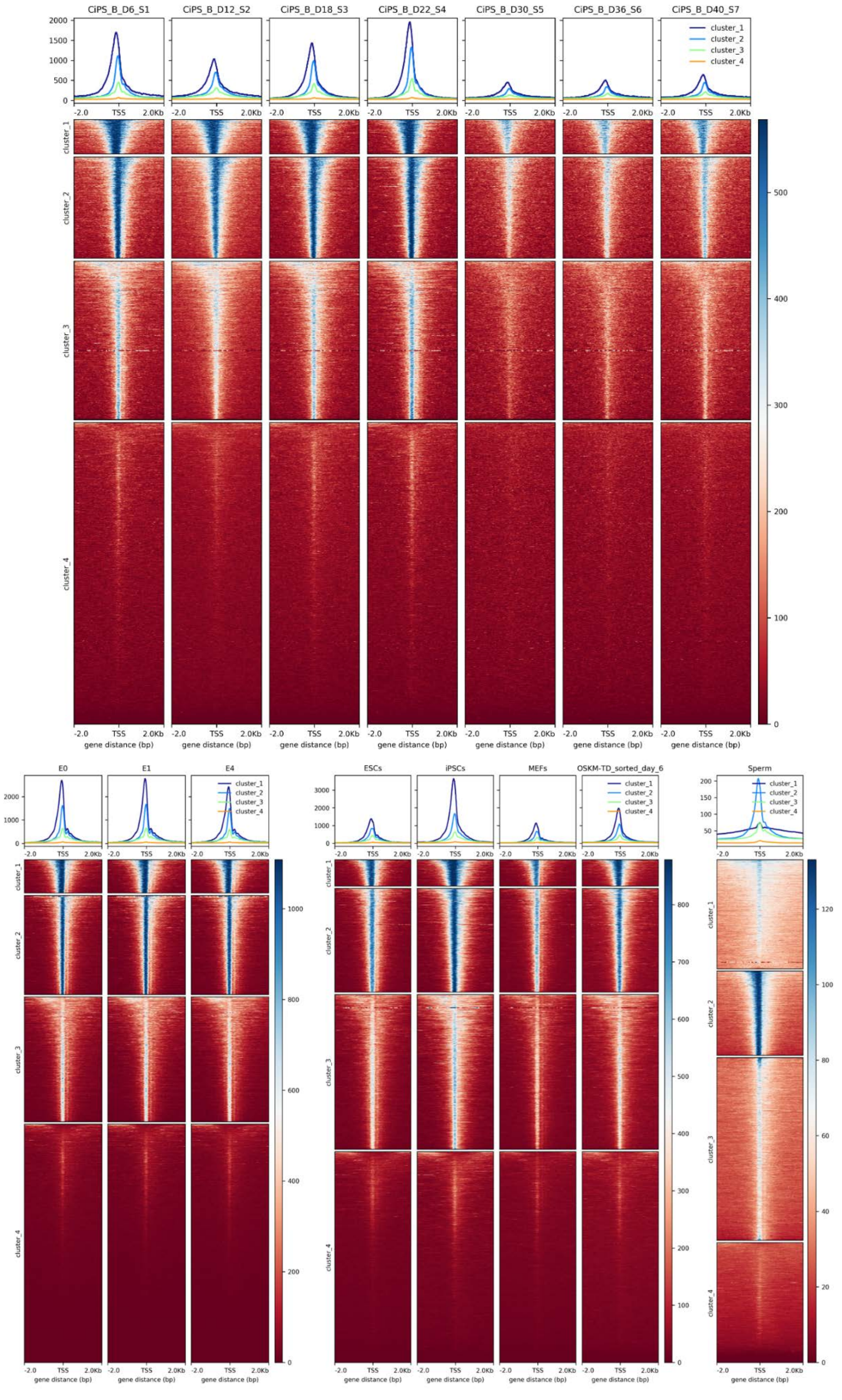
CiPS_B_D6_S1
CiPS_B_D12_S2
CiPS_B_D18_S3
CiPS_B_D22_S4
CiPS_B_D30_S5
CiPS_B_D36_S6
CiPS_B_D40_S7
cluster_1
cluster_2
cluster_3
cluster_4
-2.0
TSS
2.0Kb
gene distance (bp)
E0
E1
E4
ESCs
iPSCs
MEFs
OSKM-TD_sorted_day_6
Sperm

Figure S6. Average plots and heatmaps of ATAC-seq signals at ATAC-seq transposase hypersensitive sites (THSs) in all ATAC-seq datasets of six species. The regions in the heatmaps are ranked from highest ATAC-seq signal (top) to lowest (bottom). The cluster manually set to 4.

A. thaliana

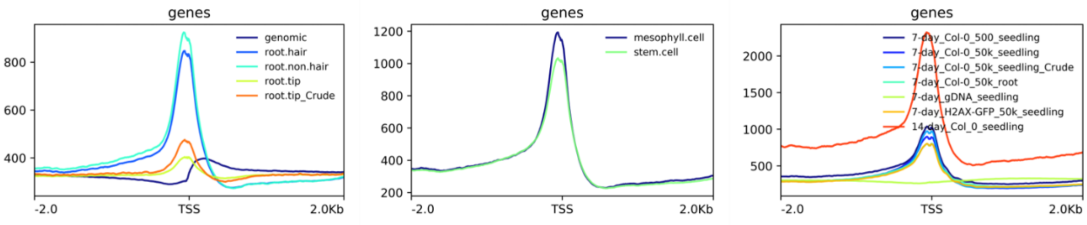

C. elegans

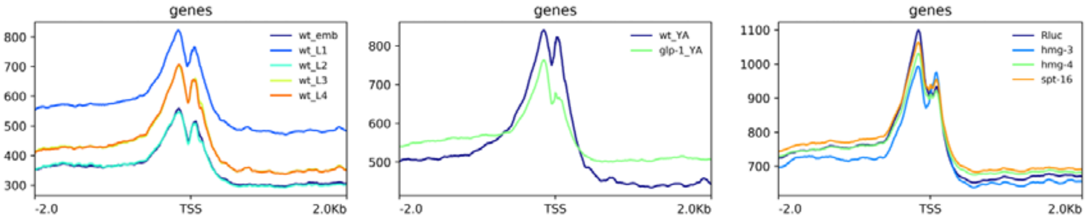

D. melanogaster

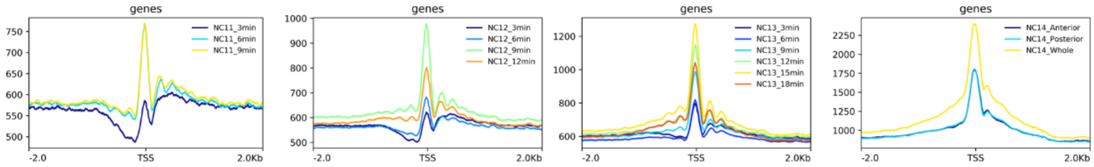

S. cerevisiae

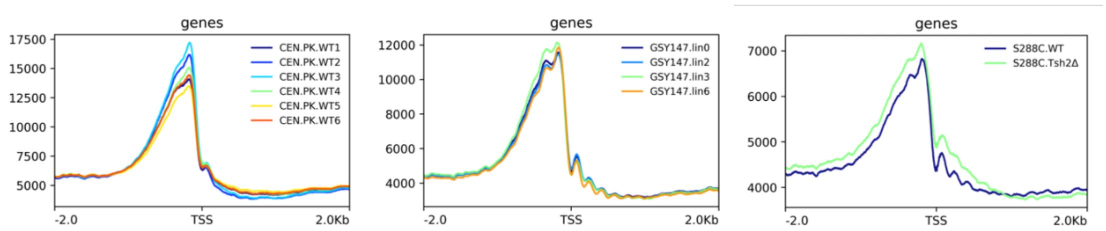

H. sapiens

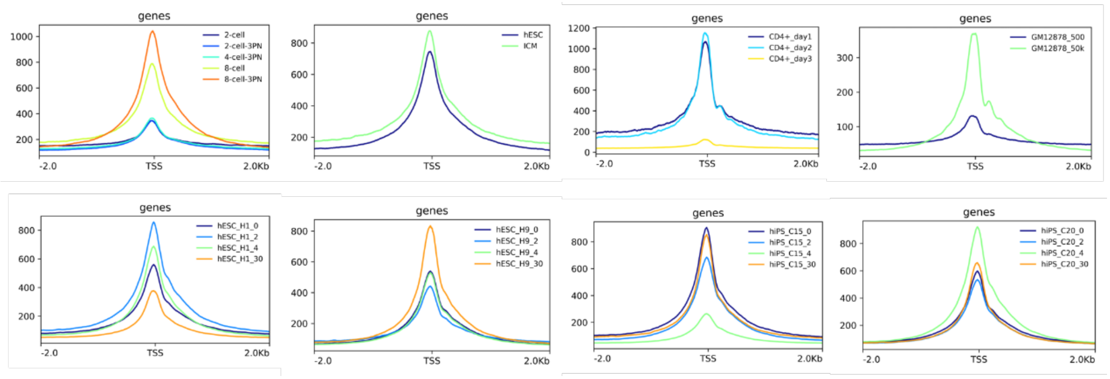

M. musculus

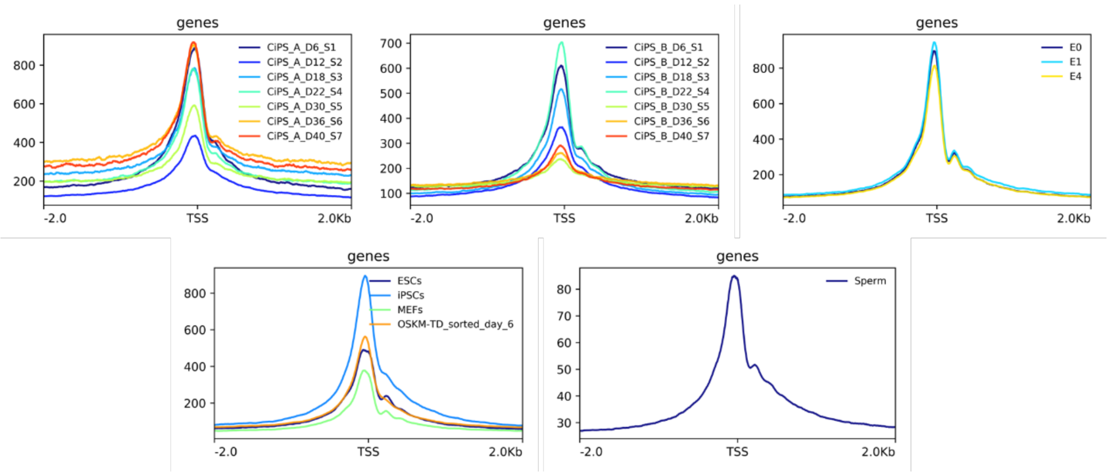

Figure S7. Distribution of accessible regions around the TSS identified from ATAC-seq samples. The center of accessible regions was used to produce the distribution plots. The upstream and downstream regions (2 kb) of TSS are mappable.

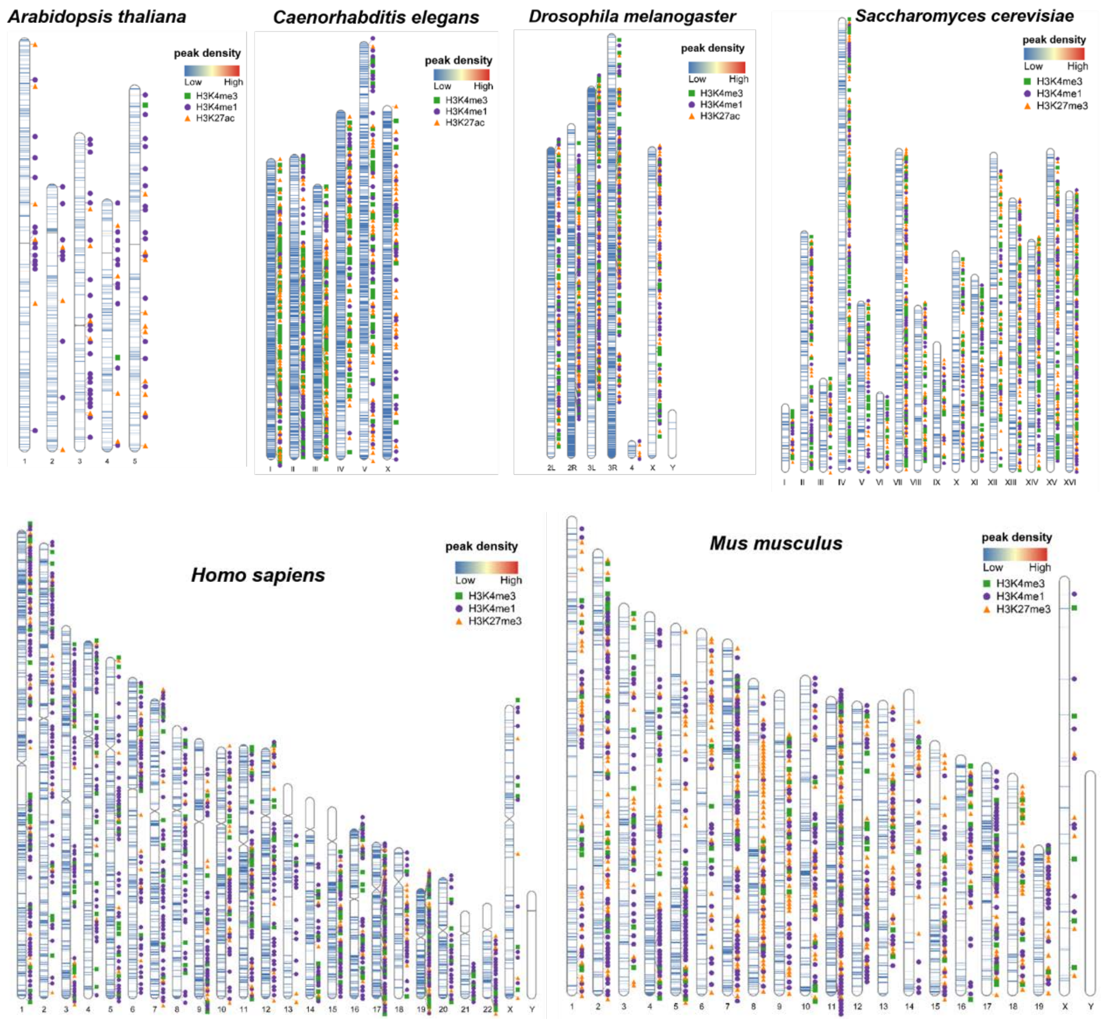

Figure S8. Peak distributions over chromosomes. Peak density was calculated by average peak counts divided by peak length (kb). Number of randomly selected histone modification sites (n): human, 1000; mouse, 1000; *Drosophila*, 500; nematode, 500; yeast, 1000; *Arabidopsis*, 100.

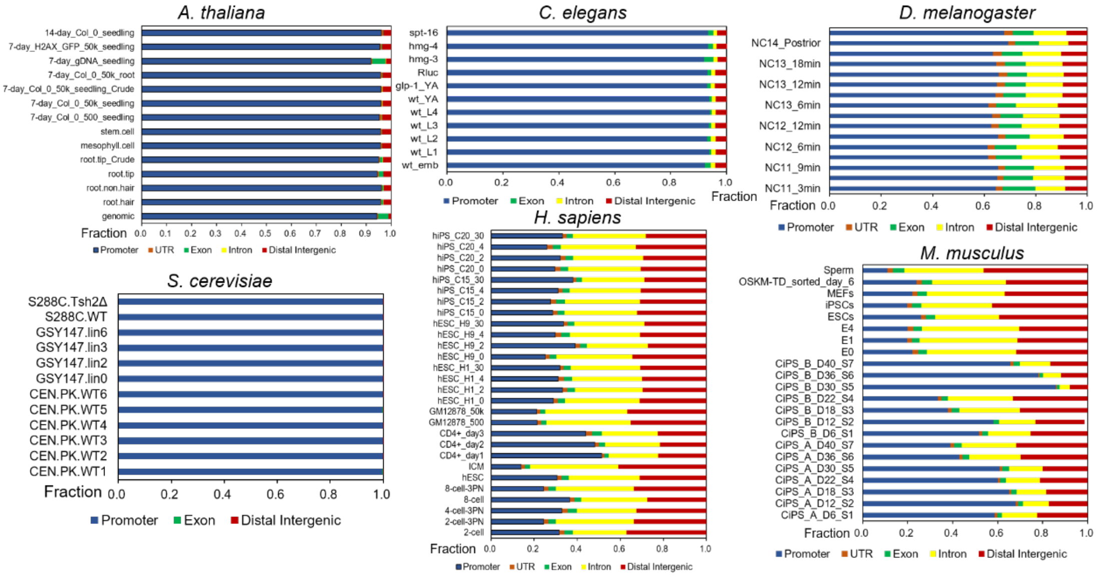

Figure S9. Genomic distributions of enriched peaks identified in all ATAC-seq datasets. Bar graph showing the genomic distribution of the different THS categories. THS peaks within TSS ± 3 kb are considered as promoter THS, and those not located in promoters, exons, introns, or UTRs are labeled as distal intergenic. The x-axis represents percentage.

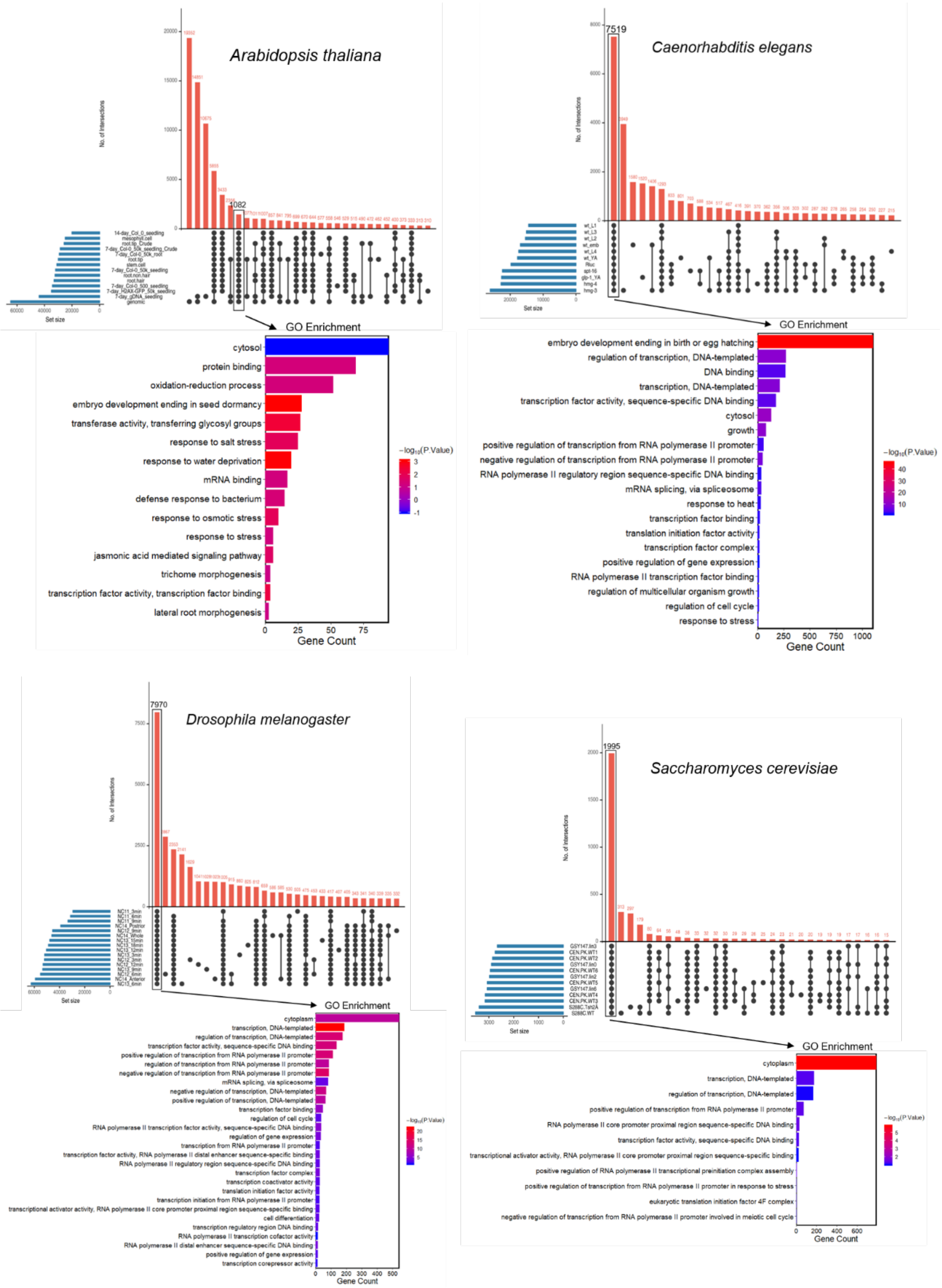
Arabidopsis thaliana
1082
GO Enrichment
cytosol
protein binding
oxidation-reduction process
embryo development ending in seed dormancy
transferase activity, transferring glycosyl groups
response to salt stress
response to water deprivation
mRNA binding
defense response to bacterium
response to osmotic stress
response to stress
jasmonic acid mediated signaling pathway
trichome morphogenesis
transcription factor activity, transcription factor binding
lateral root morphogenesis
Gene Count
Caenorhabditis elegans
7519
GO Enrichment
embryo development ending in birth or egg hatching
regulation of transcription, DNA-templated
DNA binding
transcription, DNA-templated
transcription factor activity, sequence-specific DNA binding
cytosol
growth
positive regulation of transcription from RNA polymerase II promoter
negative regulation of transcription from RNA polymerase II promoter
RNA polymerase II regulatory region sequence-specific DNA binding
mRNA splicing, via spliceosome
response to heat
transcription factor binding
translation initiation factor activity
transcription factor complex
positive regulation of gene expression
RNA polymerase II transcription factor binding
regulation of multicellular organism growth
regulation of cell cycle
response to stress
Gene Count
Drosophila melanogaster
7970
GO Enrichment
cytoplasm
transcription, DNA-templated
regulation of transcription, DNA-templated
transcription factor activity, sequence-specific DNA binding
positive regulation of transcription from RNA polymerase II promoter
regulation of transcription from RNA polymerase II promoter
negative regulation of transcription from RNA polymerase II promoter
mRNA splicing, via spliceosome
negative regulation of transcription, DNA-templated
positive regulation of transcription, DNA-templated
transcription factor binding
regulation of cell cycle
RNA polymerase II transcription factor activity, sequence-specific DNA binding
regulation of gene expression
transcription from RNA polymerase II promoter
transcription factor activity, RNA polymerase II distal enhancer sequence-specific binding
RNA polymerase II regulatory region sequence-specific DNA binding
transcription factor complex
transcription coactivator activity
translation initiation factor activity
transcription initiation from RNA polymerase II promoter
transcriptional activator activity, RNA polymerase II core promoter proximal region sequence-specific binding
cell differentiation
transcription regulatory region DNA binding
RNA polymerase II transcription cofactor activity
RNA polymerase II distal enhancer sequence-specific DNA binding
positive regulation of gene expression
transcription corepressor activity
Gene Count
Saccharomyces cerevisiae
1995
GO Enrichment
cytoplasm
transcription, DNA-templated
regulation of transcription, DNA-templated
positive regulation of transcription from RNA polymerase II promoter
RNA polymerase II core promoter proximal region sequence-specific DNA binding
transcription factor activity, sequence-specific DNA binding
transcriptional activator activity, RNA polymerase II core promoter proximal region sequence-specific binding
positive regulation of RNA polymerase II transcriptional preinitiation complex assembly
positive regulation of transcription from RNA polymerase II promoter in response to stress
eukaryotic translation initiation factor 4F complex
negative regulation of transcription from RNA polymerase II promoter involved in meiotic cell cycle
Gene Count

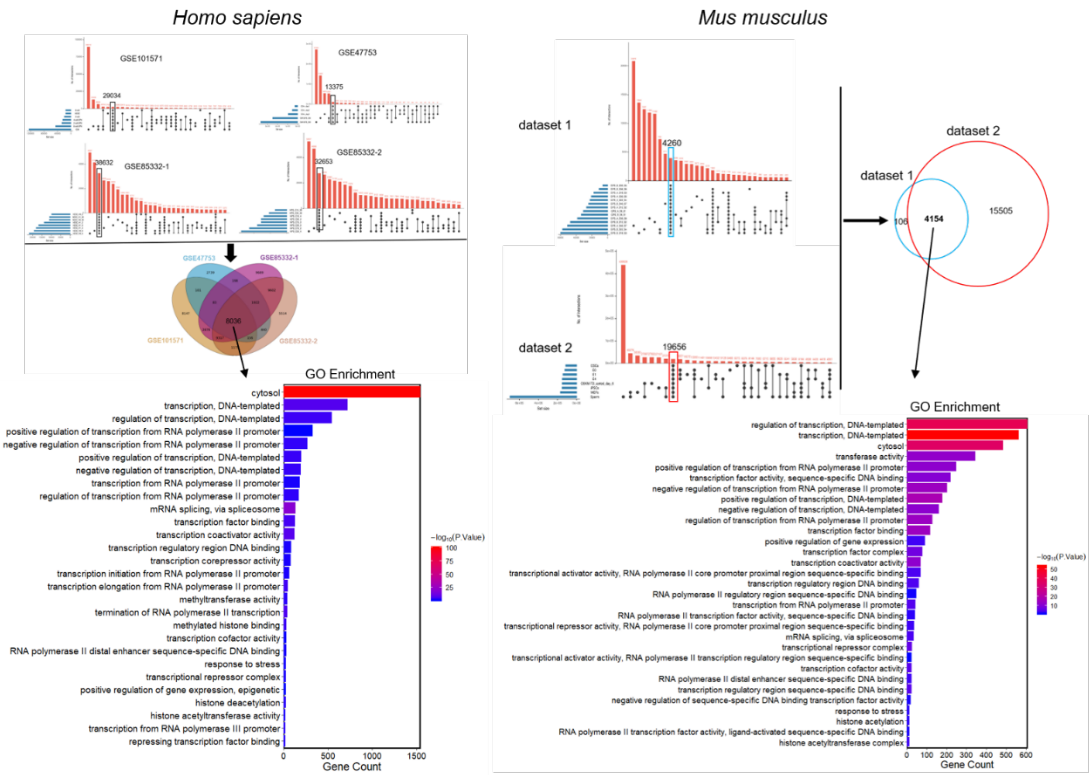

Figure S10. GO enrichment analysis of overlapped peaks in each species.

Arabidopsis thaliana

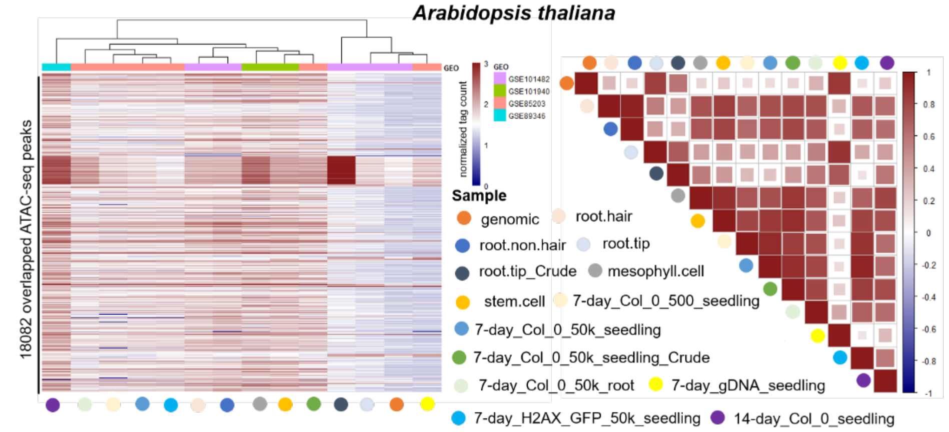

Caenorhabditis elegans

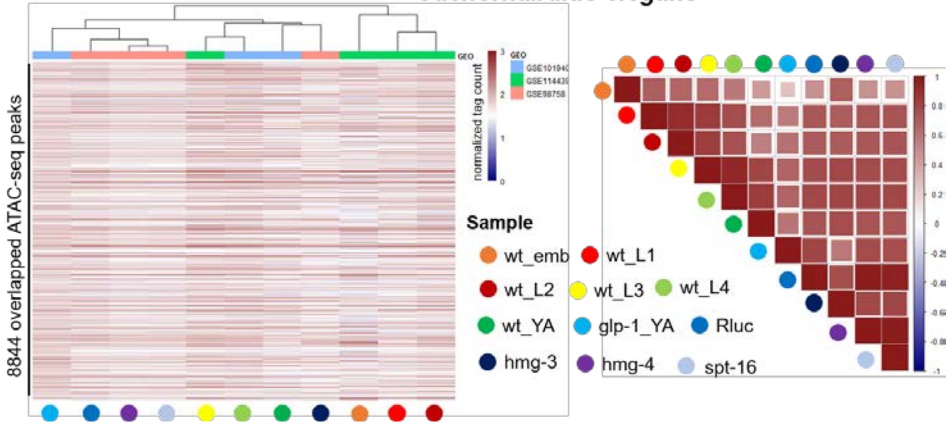

Drosophila melanogaster

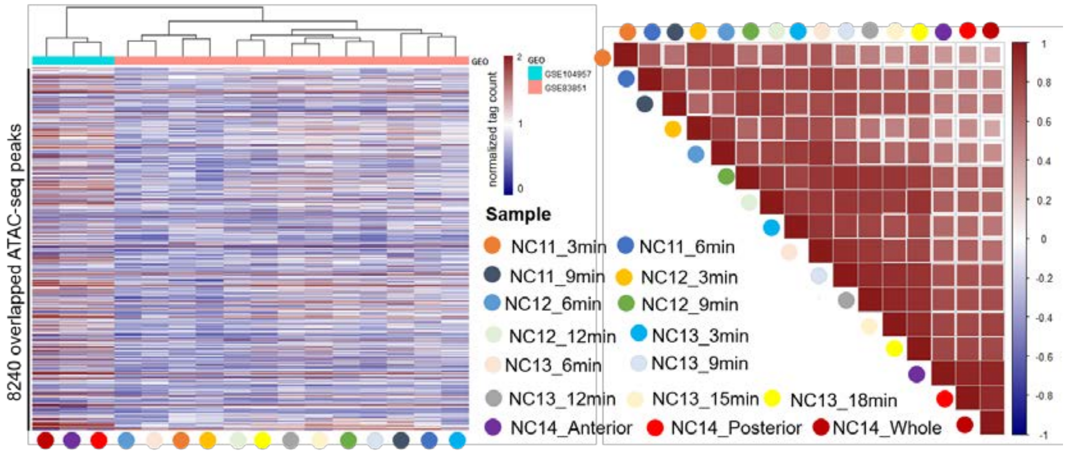

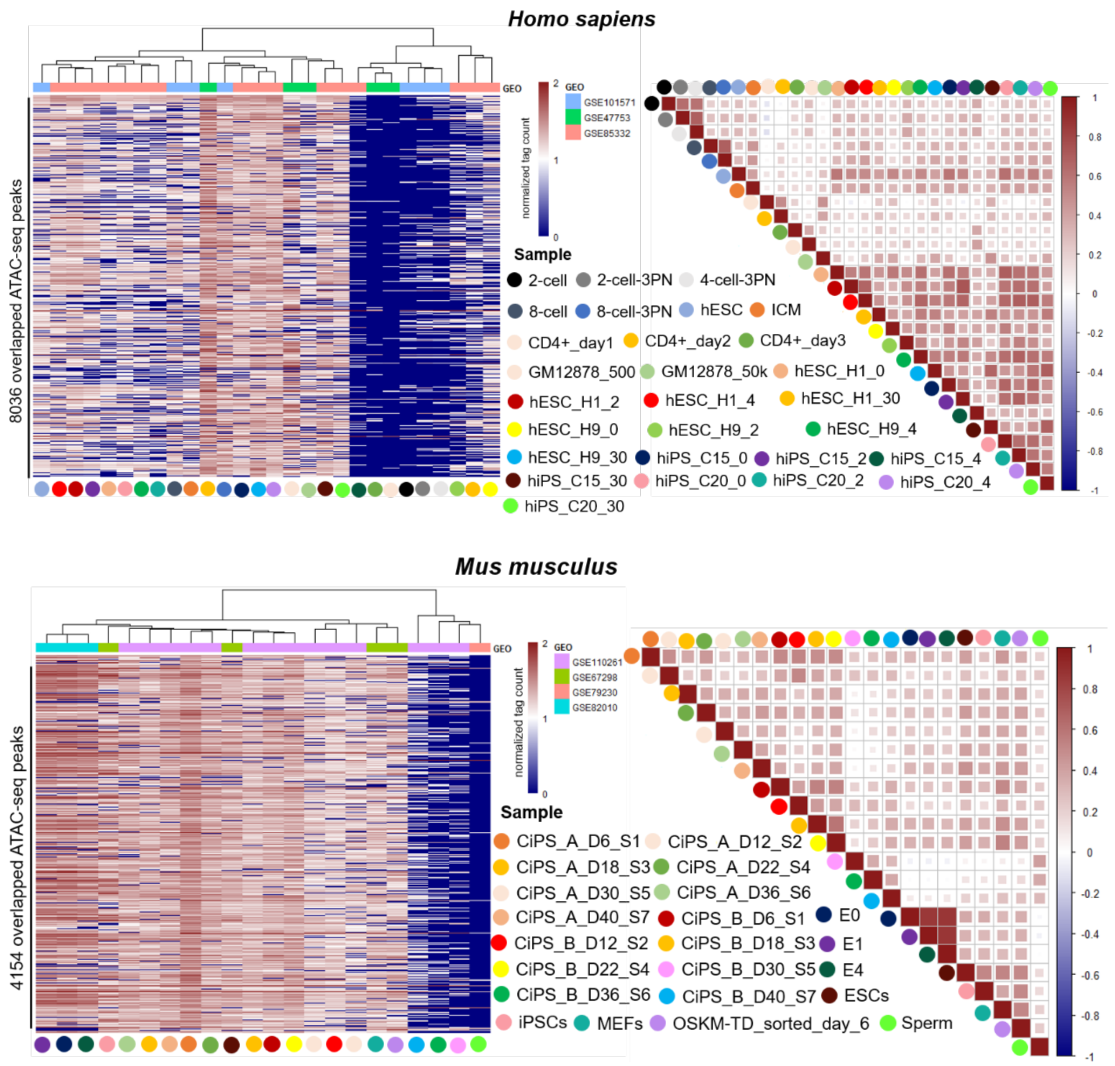

Figure S11. Heat maps show the ATAC-seq enrichment (RPKM) (left) and the comparison of ATAC-seq signal within consensus ATAC-seq peaks by Pearson's Coefficient Correlation algorithm. The colored bubbles represent different samples.

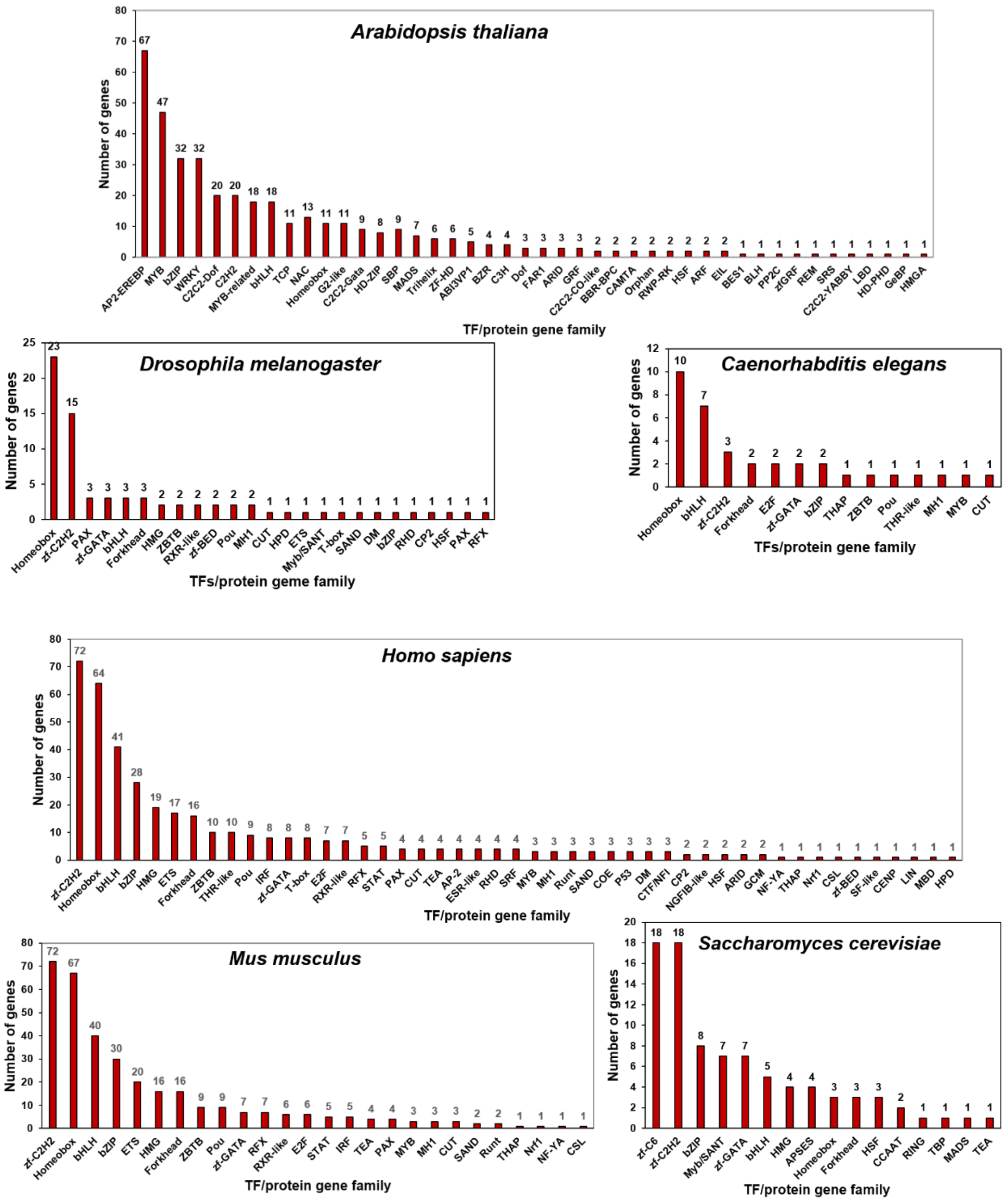

Figure S12. Gene family classification of the identified TFs. x-axis represents TF gene family; y-axis represents the enriched number of TFs. The gene family information were collected from JASPAR http://jaspar.genereg.net/.

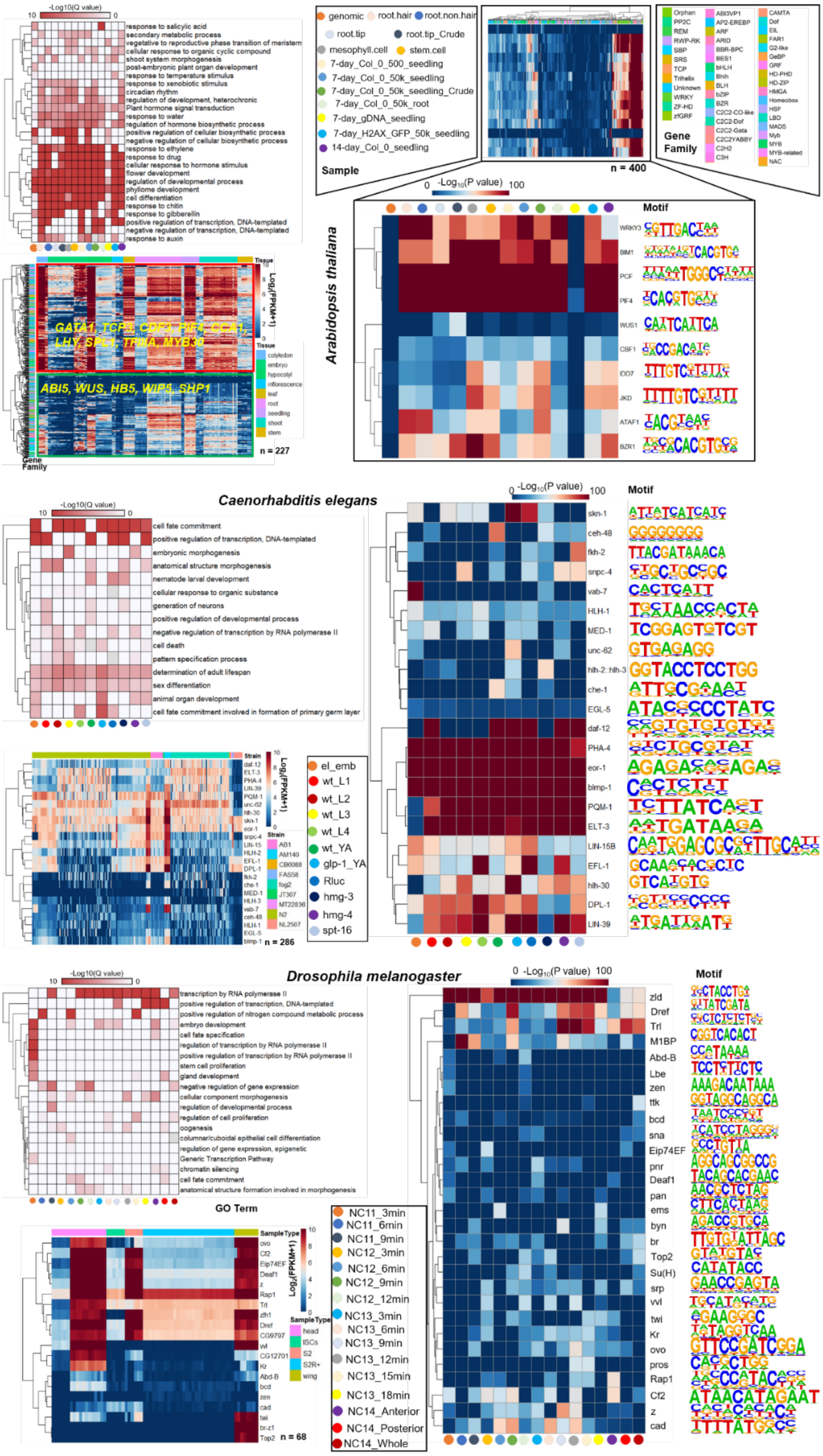

Arabidopsis thaliana
Sample
Gene Family
n = 400
n = 227
-Log10(Q value)
Log2(FPKM+1)
Tissue
Motif
GATA1, TCP3, CDF3, PIF4, CCA1, LHY, SPL1, TRIHA, MYB30
ABI5, WUS, HB5, WIP5, SHP1
genomic
root.hair
root.non.hair
root.tip
root.tip_Crude
mesophyll.cell
stem.cell
7-day_Col_0_500_seedling
7-day_Col_0_50k_seedling
7-day_Col_0_50k_seedling_Crude
7-day_Col_0_50k_root
7-day_gDNA_seedling
7-day_H2AX_GFP_50k_seedling
14-day_Col_0_seedling
WRKY3
BIM1
PCF
PIF4
WUS1
CBF1
IDD7
JKD
ATAF1
BZR1
Caenorhabditis elegans
cell fate commitment
positive regulation of transcription, DNA-templated
embryonic morphogenesis
anatomical structure morphogenesis
nematode larval development
cellular response to organic substance
generation of neurons
positive regulation of developmental process
negative regulation of transcription by RNA polymerase II
cell death
pattern specification process
determination of adult lifespan
sex differentiation
animal organ development
cell fate commitment involved in formation of primary germ layer
Strain
n = 286
el_emb
wt_L1
wt_L2
wt_L3
wt_L4
wt_YA
glp-1_YA
Rluc
hmg-3
hmg-4
spt-16
skn-1
ceh-48
fkh-2
snpc-4
vab-7
HLH-1
MED-1
unc-62
hlh-2::hlh-3
che-1
EGL-5
daf-12
PHA-4
eor-1
blmp-1
PQM-1
ELT-3
LIN-15B
EFL-1
hlh-30
DPL-1
LIN-39
Drosophila melanogaster
transcription by RNA polymerase II
positive regulation of transcription, DNA-templated
positive regulation of nitrogen compound metabolic process
embryo development
cell fate specification
regulation of transcription by RNA polymerase II
positive regulation of transcription by RNA polymerase II
stem cell proliferation
gland development
negative regulation of gene expression
cellular component morphogenesis
regulation of developmental process
regulation of cell proliferation
oogenesis
columnar/cuboidal epithelial cell differentiation
regulation of gene expression, epigenetic
Generic Transcription Pathway
chromatin silencing
cell fate commitment
anatomical structure formation involved in morphogenesis
GO Term
SampleType
n = 68
NC11_3min
NC11_6min
NC11_9min
NC12_3min
NC12_6min
NC12_9min
NC12_12min
NC13_3min
NC13_6min
NC13_9min
NC13_12min
NC13_15min
NC13_18min
NC14_Anterior
NC14_Posterior
NC14_Whole
zld
Dref
Trl
M1BP
Abd-B
Lbe
zen
ttk
bcd
sna
Eip74EF
pnr
Deaf1
pan
ems
byn
br
Top2
Su(H)
srp
vvl
twi
Kr
ovo
pros
Rap1
Cf2
z
cad

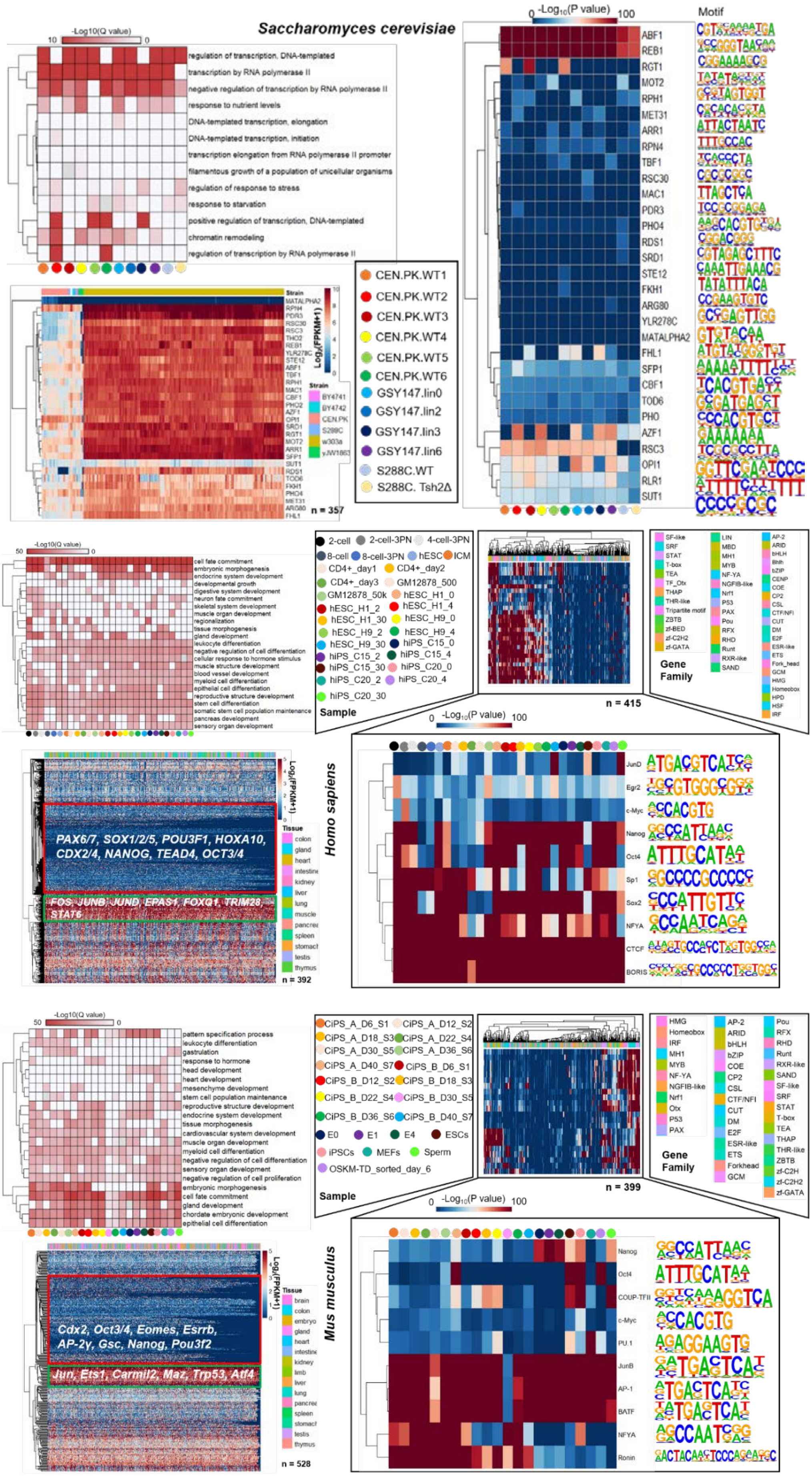
Saccharomyces cerevisiae
-Log10(Q value)
regulation of transcription, DNA-templated
transcription by RNA polymerase II
negative regulation of transcription by RNA polymerase II
response to nutrient levels
DNA-templated transcription, elongation
DNA-templated transcription, initiation
transcription elongation from RNA polymerase II promoter
filamentous growth of a population of unicellular organisms
regulation of response to stress
response to starvation
positive regulation of transcription, DNA-templated
chromatin remodeling
regulation of transcription by RNA polymerase II
CEN.PK.WT1
CEN.PK.WT2
CEN.PK.WT3
CEN.PK.WT4
CEN.PK.WT5
CEN.PK.WT6
GSY147.lin0
GSY147.lin2
GSY147.lin3
GSY147.lin6
S288C.WT
S288C. Tsh2Δ
Strain
BY4741
BY4742
CEN.PK
S288C
w303a
y.JW1863
n = 357
-Log10(P value)
Motif
ABF1
REB1
RGT1
MOT2
RPH1
MET31
ARR1
RPN4
TBF1
RSC30
MAC1
PDR3
PHO4
RDS1
SRD1
STE12
FKH1
ARG80
YLR278C
MATALPHA2
FHL1
SFP1
CBF1
TOD6
PHO
AZF1
RSC3
OPI1
RLR1
SUT1
cell fate commitment
embryonic morphogenesis
endocrine system development
developmental growth
digestive system development
neuron fate commitment
skeletal system development
muscle organ development
regionalization
tissue morphogenesis
gland development
leukocyte differentiation
negative regulation of cell differentiation
cellular response to hormone stimulus
muscle structure development
blood vessel development
myeloid cell differentiation
epithelial cell differentiation
reproductive structure development
stem cell differentiation
somatic stem cell population maintenance
pancreas development
sensory organ development
2-cell
2-cell-3PN
4-cell-3PN
8-cell
8-cell-3PN
hESC
ICM
CD4+_day1
CD4+_day2
CD4+_day3
GM12878_500
GM12878_50k
hESC_H1_0
hESC_H1_2
hESC_H1_4
hESC_H1_30
hESC_H9_0
hESC_H9_2
hESC_H9_4
hESC_H9_30
hiPS_C15_0
hiPS_C15_2
hiPS_C15_4
hiPS_C15_30
hiPS_C20_0
hiPS_C20_2
hiPS_C20_4
hiPS_C20_30
Sample
n = 415
Gene Family
Homo sapiens
PAX6/7, SOX1/2/5, POU3F1, HOXA10, CDX2/4, NANOG, TEAD4, OCT3/4
FOS, JUNB, JUND, EPAS1, FOXQ1, TRIM28, STAT6
Tissue
n = 392
JunD
Egr2
c-Myc
Nanog
Oct4
Sp1
Sox2
NFYA
CTCF
BORIS
pattern specification process
leukocyte differentiation
gastrulation
response to hormone
head development
heart development
mesenchyme development
stem cell population maintenance
reproductive structure development
endocrine system development
tissue morphogenesis
cardiovascular system development
muscle organ development
myeloid cell differentiation
negative regulation of cell differentiation
sensory organ development
negative regulation of cell proliferation
embryonic morphogenesis
cell fate commitment
gland development
chordate embryonic development
epithelial cell differentiation
CiPS_A_D6_S1
CiPS_A_D12_S2
CiPS_A_D18_S3
CiPS_A_D22_S4
CiPS_A_D30_S5
CiPS_A_D36_S6
CiPS_A_D40_S7
CiPS_B_D6_S1
CiPS_B_D12_S2
CiPS_B_D18_S3
CiPS_B_D22_S4
CiPS_B_D30_S5
CiPS_B_D36_S6
CiPS_B_D40_S7
E0
E1
E4
ESCs
iPSCs
MEFs
Sperm
OSKM-TD_sorted_day_6
Sample
n = 399
Gene Family
Mus musculus
Cdx2, Oct3/4, Eomes, Esrrb, AP-2γ, Gsc, Nanog, Pou3f2
Jun, Ets1, Carmil2, Maz, Trp53, Atf4
Tissue
n = 528
Nanog
Oct4
COUP-TFII
c-Myc
PU.1
JunB
AP-1
BATF
NFYA
Ronin

Figure S13. Functional and expression pattern analysis of all TFs identified in six species. The right part, annotation of all TFs and randomly selected 10 for motif analysis. The top left, heatmaps showing the top 20 enriched GO terms of all TFs using Metascape enrichment. The bottom left, expression patterns of all TFs across different tissues or strains. For *Arabidopsis*, *H. sapiens*, and *M. musculus*, the TFs in red box were hardly expressed, and in green box were highly expressed. The transcriptomic expression profiles were obtained from public available databases and previously published researches, including human and mouse https://www.encodeproject.org/., fruit fly GSE99665, GSE110240, GSE125385, GSE118726, GSE129292, and GSE124254 [1-6], nematode GSE103776, GSE126585, GSE108283, GSE117471, GSE112053, GSE108283, GSE98574, GSE115095, GSE117700, GSE124178, GSE113266 [7-13], *Arabidopsis* GSE81202, GSE115584, GSE121003, GSE123010, GSE101422, GSE110500, GSE117416, GSE117296, GSE122770, GSE126782, GSE127759, GSE120672, GSE130729, GSE83573, GSE61291, GSE121407, and GSE126624. [14-29], and yeast GSE125038, GSE126435, GSE101290, GSE111815, and GSE115556. [30-34]. All raw expression matrices were normalized via $\log_2$FPKM+1.

Homo sapiens

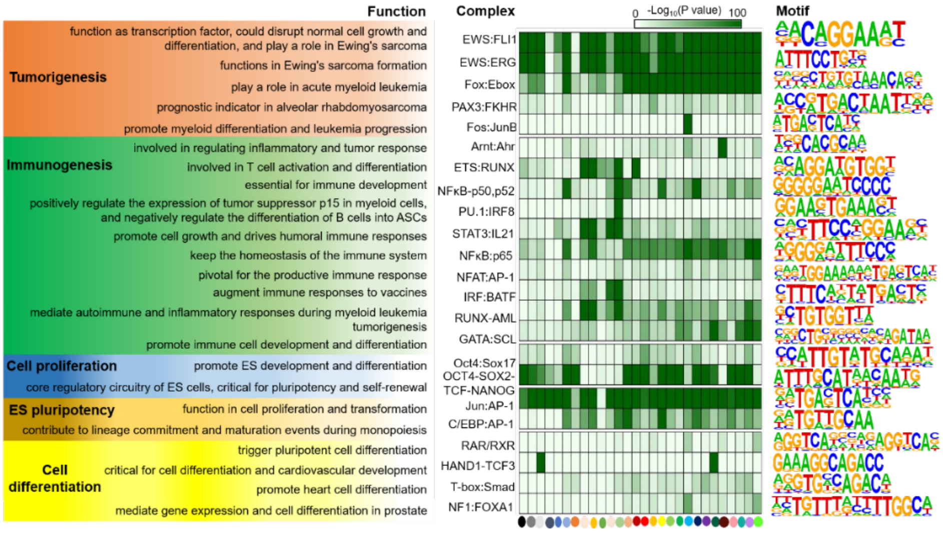

Mus musculus

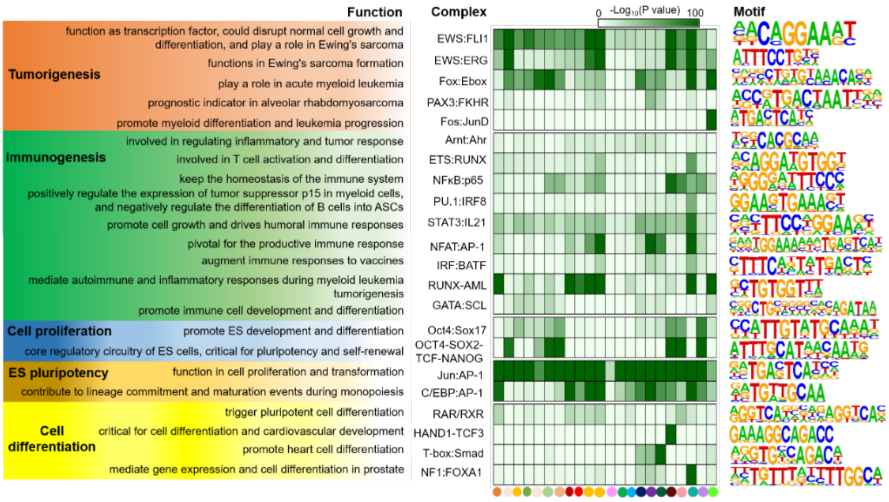

Figure S14. TF complexes/fusions play multiple functions during life span. The colored bubbles indicate different samples which was annotated in Figs. S11 and S13.

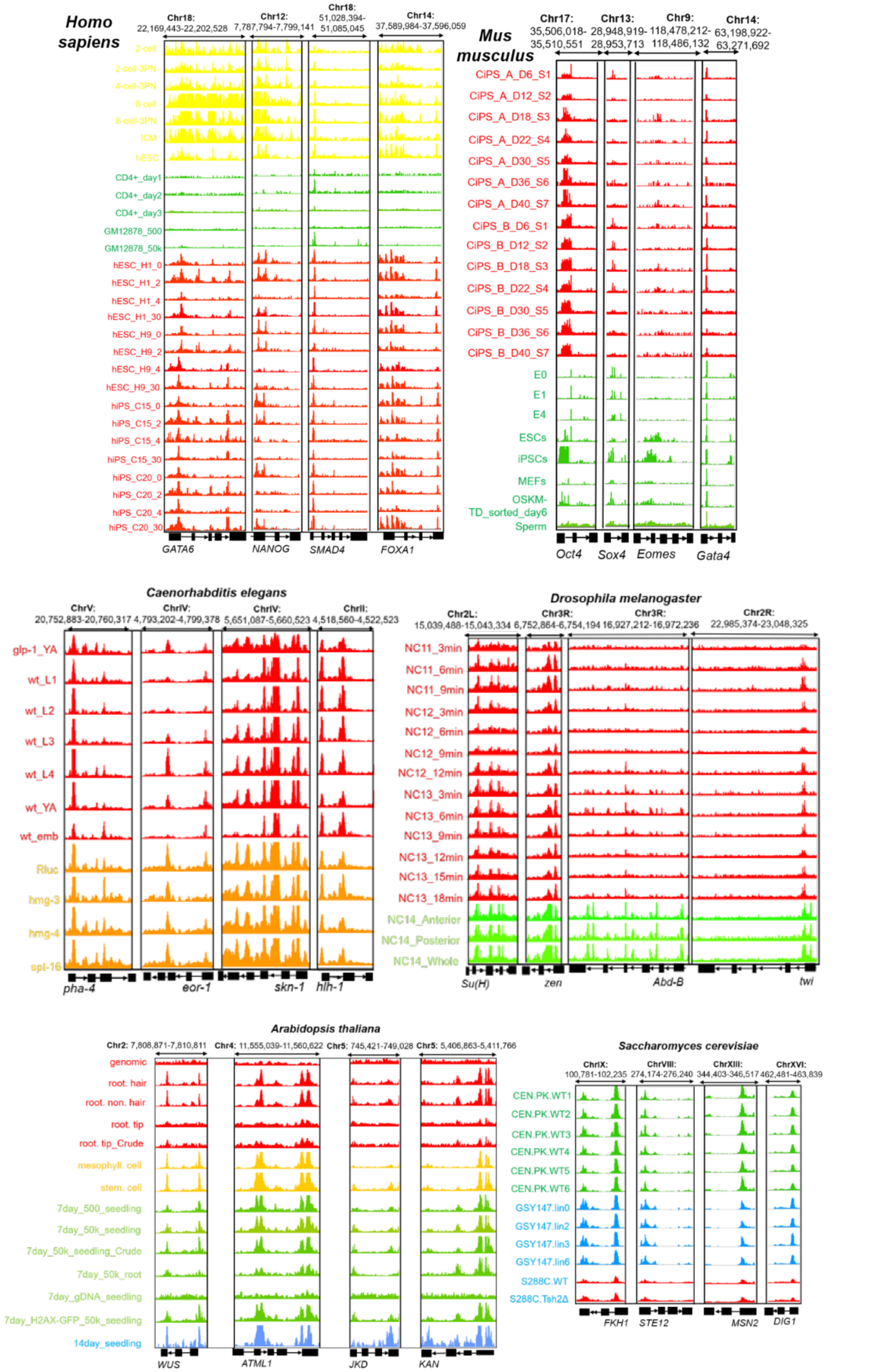

Figure S15. The IGV views showing the ATAC-seq enrichment near key cell-fate-determined TFs during early embryogenesis.

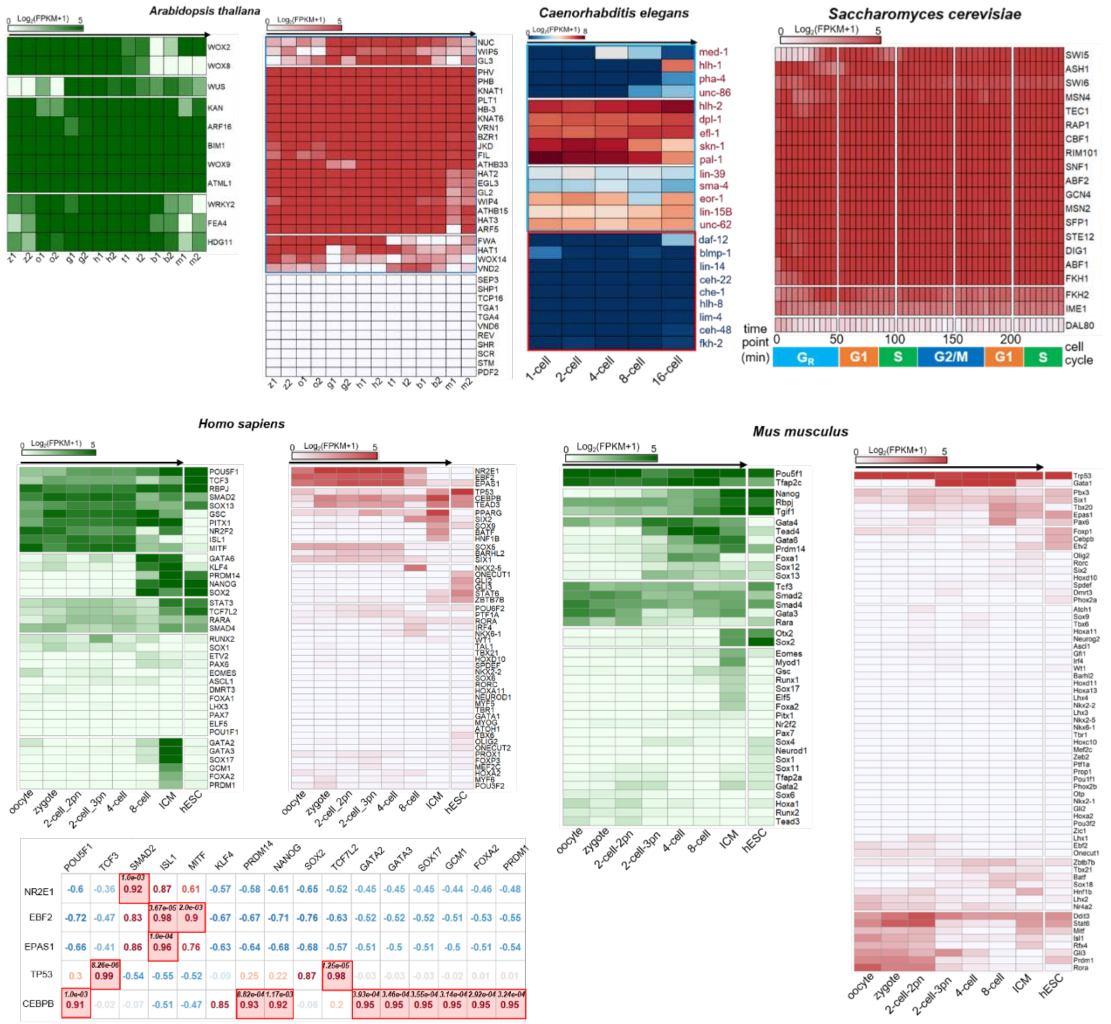

	POU5F1	TCF3	SMAD2	ISL1	MITF	KLF4	PRDM14	NANOG	SOX2	TCF7L2	GATA2	GATA3	SOX17	GCM1	FOXA2	PRDM1
NR2E1	-0.6	-0.36	1.0e-03 0.92	0.87	0.61	-0.57	-0.58	-0.61	-0.65	-0.52	-0.45	-0.45	-0.45	-0.44	-0.46	-0.48
EBF2	-0.72	-0.47	0.83	3.67e-05 0.98	2.0e-03 0.9	-0.67	-0.67	-0.71	-0.76	-0.63	-0.52	-0.52	-0.52	-0.51	-0.53	-0.55
EPAS1	-0.66	-0.41	0.86	1.0e-04 0.96	0.76	-0.63	-0.64	-0.68	-0.68	-0.57	-0.51	-0.5	-0.51	-0.5	-0.51	-0.54
TP53	0.3	8.26e-06 0.99	-0.54	-0.55	-0.52	-0.09	0.25	0.22	0.87	1.25e-05 0.98	-0.03	-0.03	-0.03	-0.02	0.01	0.01
CEBPB	1.0e-03 0.91	-0.02	-0.07	-0.51	-0.47	0.85	8.82e-04 0.93	1.17e-03 0.92	-0.06	0.2	3.93e-04 0.95	3.46e-04 0.95	3.55e-04 0.95	3.14e-04 0.95	2.92e-04 0.95	3.24e-04 0.95

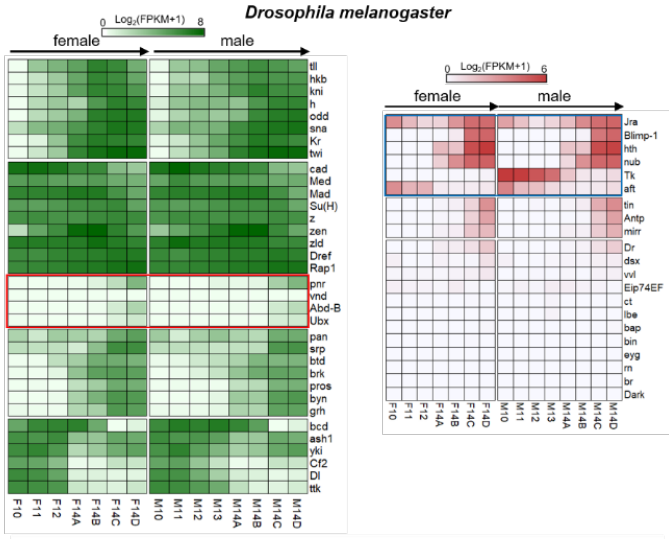

	h	odd	sna	twi	Mad	Rap1	pnr	Abd-B	Ubx	pan	srp	btd	brk	pros	byn	grh	bcd
Jra	7.9e-07 0.93	5.45e-06 0.9	0.88	0.85	-0.84	0.82	0.81	0.81	0.91	0.85	9.78e-07 0.92	0.78	0.83	4.71e-07 0.93	2.6e-07 0.94	1.3e-06 0.92	2.53e-06 -0.91
Blimp-1	3.18e-06 0.91	0.85	0.82	0.8	4.83e-06 -0.9	0.86	0.89	2.67e-06 0.91	7.17e-10 0.97	5.73e-08 0.95	1.92e-11 0.99	0.71	0.77	7.65e-09 0.96	4.13e-09 0.97	3.86e-09 0.97	-0.75
hth	0.85	0.82	0.78	0.79	-0.87	0.88	1.18e-06 0.92	4.53e-09 0.97	9.12e-12 0.99	3.78e-10 0.98	4.48e-08 0.95	0.68	0.75	1.17e-06 0.92	1.41e-06 0.92	1.21e-06 0.92	-0.72
nub	5.18e-10 0.98	1.51e-08 0.96	2.76e-07 0.94	7.47e-07 0.93	-0.79	5.67e-07 0.93	0.83	0.86	2.57e-07 0.94	4.93e-06 0.9	1.24e-08 0.96	0.87	3.55e-06 0.9	2.49e-10 0.98	9.3e-11 0.98	1.44e-08 0.96	-0.84
Tk	-0.4	-0.45	-0.5	-0.52	0.12	-0.47	-0.28	-0.29	-0.32	-0.3	-0.33	-0.51	-0.49	-0.4	-0.4	-0.37	0.51
aft	-0.51	-0.53	-0.61	-0.61	0	-0.64	-0.3	-0.31	-0.34	-0.31	-0.37	-0.59	-0.57	-0.45	-0.45	-0.42	0.14

Figure S16. Expression profiles of TFs involved in early cell fate determination. Left figures show the expression patterns of stat-of-the-art early cell fate determined TFs during early embryonic development. Right figures exhibit the expression profiles of cell-fate TFs during early embryogenesis. For *Drosophila* and human, the bottom figures display the expression correlations between previously certificated early embryonic cell-fate TFs and some cell-fate TFs highly expressed during early embryogenesis in this study. Note: For *Arabidopsis*, z1/2, zygote stage; o1/2, octant stage; g1/2, globular stage; h1/2, heart stage; t1/2. torpedo stage; b1/2, bent stage; m1/2, mature stage. For *Drosophila*, F, female; M, male; 10-14, nuclear cycle 10-14. TFs in red box, early embryonic TFs lowly expressed during early development, TFs in blue box, cell fate determined TFs highly expressed during early development. The expression profiles were all obtained from NCBI, including human GSE101571 [35], mouse GSE66582 [36], fruit fly GSE25180 [37], nematode GSE77944 [38], *Arabidopsis* GSE123010 [17], and yeast GSE104904 and GSE80474 [39,40]. All expression matrices were normalized via log2FPKM+1.

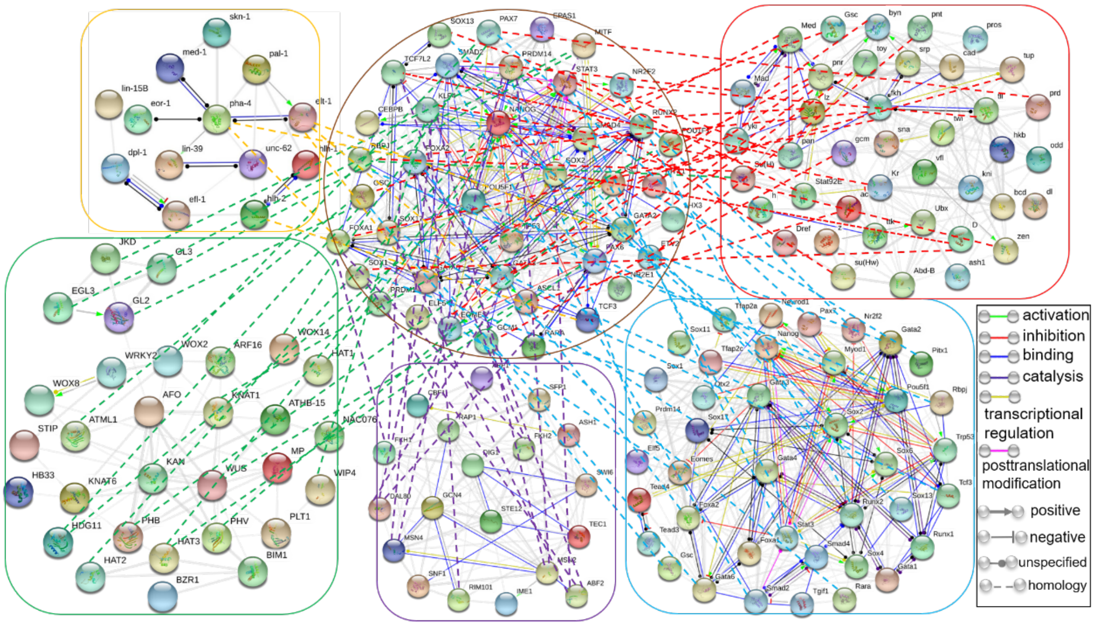

Figure S17. Transcriptional regulatory circuit of cell fate decisions during early embryogenesis. The colored dashed lines represent for TFs that homolog to human TFs, in which blue represents for mouse, red for Drosophila, orange for C. elegans, purple yeast, green for Arabidopsis. The brown circled circuits represent human, blue for mouse, red for Drosophila, orange for C. elegans, and green for Arabidopsis. The regulatory relationships were all predicted by STRING database.

Reference

1. Lehmann M, Hebbar S, Brandl H *et al*. Mutations in the *Drosophila* splicing regulator *Prp31* as a model for Retinitis pigmentosa 11. *bioRxiv* 147918 (2017). Available from: https://doi.org/10.1101/147918
2. Riahi H, Brekelmans C, Foriel S *et al*. The histone methyltransferase G9a regulates tolerance to oxidative stress-induced energy consumption. *PLoS Biol.* 17(3), e2006146 (2019).
3. Rodriguez-Fernandez IA, Qi Y, Jasper H. Loss of a proteostatic checkpoint in intestinal stem cells contributes to age-related epithelial dysfunction. *Nat. Commun.* 10(1), 1050 (2019).
4. Lam KC, Chung HR, Semplicio G *et al*. The NSL complex-mediated nucleosome landscape is required to maintain transcription fidelity and suppression of transcription noise. *Genes Dev.* 33(7-8), 452-465 (2019).
5. Zirin J, Ni X, Sack LM *et al*. Interspecies analysis of MYC targets identifies tRNA synthetases as mediators of growth and survival in MYC-overexpressing cells. *Proc. Natl. Acad. Sci. USA* 116(29), 14614-14619 (2019).
6. Uyehara CM, McKay DJ. Direct and widespread role for the nuclear receptor EcR in mediating the response to ecdysone in Drosophila. *Proc. Natl. Acad. Sci. USA* 116(20), 9893-9902 (2019).
7. Çelen I, Doh JH, Sabanayagam CR. Effects of liquid cultivation on gene expression and phenotype of C. elegans. *BMC Genomics* 19(1), 562 (2018).
8. Levine A, Grushko D, Cohen E. Gene expression modulation by the linker of nucleoskeleton and cytoskeleton complex contributes to proteostasis. *Aging Cell* 18(6), e13047 (2019).
9. Pender CL, Horvitz HR. Hypoxia-inducible factor cell non-autonomously regulates C. elegans stress responses and behavior via a nuclear receptor. *Elife* 7, e36828 (2018).
10. Zhou L, He B, Deng J, Pang S, Tang H. Histone acetylation promotes long-lasting defense responses and longevity following early life heat stress. *PLoS Genet.* 15(4), e1008122 (2019).
11. Peterson ND, Cheesman HK, Liu P *et al*. The nuclear hormone receptor NHR-86 controls anti-pathogen responses in C. elegans. *PLoS Genet*. 15(1), e1007935 (2019).
12. Finger F, Ottens F, Springhorn A *et al*. Olfaction regulates organismal proteostasis and longevity via microRNA-dependent signaling. *Nat. Metab.* 1(3), 350-359 (2019).
13. Camacho J, Truong L, Kurt Z *et al*. The Memory of Environmental Chemical Exposure in C. elegans Is Dependent on the Jumonji Demethylases jmjd-2 and jmjd-3/utx-1. *Cell Rep*. 23(8), 2392-2404 (2018).
14. Kohnen MV, Schmid-Siegert E, Trevisan M *et al*. Neighbor Detection Induces Organ-Specific Transcriptomes, Revealing Patterns Underlying Hypocotyl-Specific Growth. *Plant Cell* 28(12), 2889-2904 (2016).
15. Dong J, Sun N, Yang J *et al*. The Transcription Factors TCP4 and PIF3 Antagonistically Regulate Organ-Specific Light Induction of *SAUR* Genes to Modulate Cotyledon Opening during De-Etiolation in Arabidopsis. *Plant Cell* 31(5), 1155-1170 (2019).
16. Zhao P, Zhou X, Shen K *et al*. Two-Step Maternal-to-Zygotic Transition with Two-Phase Parental

Genome Contributions. *Dev. Cell* 49(6), 882-893 (2019).

17. Gao P, Xiang D, Quilichini TD *et al*. Gene expression atlas of embryo development in Arabidopsis. *Plant Reprod*. 32(1), 93-104 (2019).
18. Ikeuchi M, Iwase A, Rymen B *et al*. Wounding Triggers Callus Formation via Dynamic Hormonal and Transcriptional Changes. *Plant Physiol*. 175(3), 1158-1174 (2017).
19. Chen D, Yan WH, Fu LY, Kaufmann K. Architecture of gene regulatory networks controlling flower development in Arabidopsis thaliana. *Nat. Commun*. 9, 4543 (2018).
20. Zhang Y, Yin B, Zhang J *et al*. Histone Deacetylase HDT1 is Involved in Stem Vascular Development in Arabidopsis. *Int. J. Mol. Sci.* 20(14), 3452 (2019).
21. Zandalinas SI, Sengupta S, Burks D, Azad RK, Mittler R. Identification and characterization of a core set of ROS wave-associated transcripts involved in the systemic acquired acclimation response of Arabidopsis to excess light. *Plant J.* 98(1), 126-141 (2019).
22. Tannenbaum M, Sarusi-Portuguez A, Krispil R *et al*. Regulatory chromatin landscape in *Arabidopsis thaliana* roots uncovered by coupling INTACT and ATAC-seq. *Plant Methods* 14, 113 (2018).
23. Park EY, Tsuyuki KM, Hu F, Lee J, Jeong J. PRC2-Mediated H3K27me3 Contributes to Transcriptional Regulation of FIT-Dependent Iron Deficiency Response. *Front. Plant Sci*. 10, 627 (2019).
24. Diaz-Tielas C, Graña E, Sánchez-Moreiras AM *et al*. Transcriptome responses to the natural phytotoxin t-chalcone in Arabidopsis thaliana L. *Pest Manag. Sci*. 75(9), 2490-2504 (2019).
25. Bao Y, Bassham DC, Howell SH. A Functional Unfolded Protein Response Is Required for Normal Vegetative Development. *Plant Physiol.* 179(4), 1834-1843 (2019).
26. Jia Y, Ding Y, Shi Y et al. The cbfs triple mutants reveal the essential functions of CBFs in cold acclimation and allow the definition of CBF regulons in Arabidopsis. *New Phytol*. 212(2), 345-353 (2016).
27. Zhang F, Wang L, Lim JY *et al*. Phosphorylation of CBP20 Links MicroRNA to Root Growth in the Ethylene Response. *PLoS Genet.* 12(11), e1006437 (2016).
28. Gursanscky NR, Jouannet V, Grünwald K *et al*. MOL1 is required for cambium homeostasis in Arabidopsis. *Plant J*. 86(3), 210-220 (2016).
29. Zhang X, Zhang Y, Wang T *et al*. A Comprehensive Map of Intron Branchpoints and Lariat RNAs in Plants. *Plant Cell* 31(5), 956-973 (2019).
30. Santos DA, Shi L, Tu BP, Weissman JS. Cycloheximide can distort measurements of mRNA levels and translation efficiency. *Nucleic Acids Res.* 47(10), 4974-4985 (2019).
31. McFarland MR, Keller CD, Childers BM *et al*. The molecular aetiology of tRNA synthetase depletion: induction of a GCN4 amino acid starvation response despite homeostatic maintenance of charged tRNA levels. *Nucleic Acids Res*. 48(6), 3071-3088 (2020).
32. Gowans GJ, Schep AN, Wong KM *et al*. INO80 Chromatin Remodeling Coordinates Metabolic Homeostasis with Cell Division. *Cell Rep.* 22(3), 611-623 (2018).
33. Dronamraju R, Hepperla AJ, Shibata Y *et al*. Spt6 Association with RNA Polymerase II Directs mRNA Turnover During Transcription. *Mol. Cell* 70(6), 1054-1066 (2018).

34. Mace K, Krakowiak J, El-Samad H, Pincus D. Multi-kinase control of environmental stress responsive transcription. *PLoS One* 15(3), e0230246 (2020).
35. Wu J, Xu J, Liu B *et al*. Chromatin analysis in human early development reveals epigenetic transition during ZGA. *Nature* 557(7704), 256-260 (2018).
36. Wu J, Huang B, Chen H *et al*. The landscape of accessible chromatin in mammalian preimplantation embryos. *Nature* 534(7609), 652-657 (2016).
37. Lott SE, Villalta JE, Schroth GP *et al*. Noncanonical compensation of zygotic X transcription in early Drosophila melanogaster development revealed through single-embryo RNA-seq. *PLoS Biol.* 9(2), e1000590 (2011).
38. Tintori SC, Osborne Nishimura E, Golden P, Lieb JD, Goldstein B. A Transcriptional Lineage of the Early C. elegans Embryo. *Dev. Cell* 38(4), 430-444 (2016).
39. Kelliher CM, Foster MW, Motta FC *et al*. Layers of regulation of cell-cycle gene expression in the budding yeast Saccharomyces cerevisiae. *Mol. Biol. Cell* 29(22), 2644-2655 (2018).
40. Kelliher CM, Leman AR, Sierra CS, Haase SB. Investigating Conservation of the Cell-Cycle-Regulated Transcriptional Program in the Fungal Pathogen, Cryptococcus neoformans. *PLoS Genet.* 12(12), e1006453 (2016).
